# Gut microbiota regulate maturation and mitochondrial function of the nutrient-sensing enteroendocrine cell

**DOI:** 10.1242/dev.202544

**Published:** 2024-04-30

**Authors:** Alfahdah Alsudayri, Shane Perelman, Melissa Brewer, Annika Chura, Madelyn McDevitt, Catherine Drerup, Lihua Ye

**Affiliations:** ^1^ Department of Neuroscience, The Ohio State University Wexner Medical Center, Columbus, OH 43210, USA; ^2^ Department of Integrative Biology, University of Wisconsin-Madison, Madison, WI 53706, USA

**Keywords:** Enteroendocrine cells, Gut microbiota, Mitochondria, *In vivo* imaging, Zebrafish

## Abstract

Enteroendocrine cells (EECs) are crucial for sensing ingested nutrients and regulating feeding behavior. How gut microbiota regulate the nutrient-sensing EEC activity is unclear. Our transcriptomic analysis demonstrates that commensal microbiota colonization significantly increases the expression of many genes associated with mitochondrial function. Using new methods to image EEC cytoplasmic and mitochondrial Ca^2+^ activity in live zebrafish, our data revealed that it is dynamically regulated during the EEC development process. Mature EECs display an increased mitochondrial-to-cytoplasmic Ca^2+^ ratio. Mitochondria are evenly distributed in the cytoplasm of immature EECs. As EECs mature, their mitochondria are highly localized at the basal membrane where EEC vesicle secretion occurs. Conventionalized (CV) EECs, but not germ-free (GF) EECs, exhibit spontaneous low-amplitude Ca^2+^ fluctuation. The mitochondrial-to-cytoplasmic Ca^2+^ ratio is significantly higher in CV EECs. Nutrient stimulants, such as fatty acid, increase cytoplasmic Ca^2+^ in a subset of EECs and promote a sustained mitochondrial Ca^2+^ and ATP increase. However, the nutrient-induced EEC mitochondrial activation is nearly abolished in GF zebrafish. Together, our study reveals that commensal microbiota are crucial in supporting EEC mitochondrial function and maturation.

## Teaser

**Highlighted Article:** Tracking nutrient-sensing enteroendocrine cells cellular and mitochondrial Ca^2+^ activity in live zebrafish during development reveals that gut microbiota play crucial roles in promoting EEC maturation and mitochondrial function.

## INTRODUCTION

Feeding behavior is conserved among all organisms. Within the intestinal epithelium, a group of specialized sensory cells, known as enteroendocrine cells (EECs), sense the ingested nutrient information and secrete hormone molecules to regulate physiological homeostasis (Furness et al., 2013; Sanchez et al., 2022). The nutrient-sensing function of EECs is highly conserved among organisms, including zebrafish (Furness et al., 2013; Ye et al., 2019). The EECs are dispersed along the digestive tract and make up less than 1% of the intestinal epithelium cells (IECs). However, collectively, the EECs form the largest endocrine organ in the body (Furness et al., 2013). Most of the previous studies assessing EECs have been focused on adults. It is well-known that ingested nutrients, such as fatty acids or glucose, directly stimulate the EECs by triggering a cascade of membrane depolarization, action potential firing, voltage-dependent Ca^2+^ entry and hormone-containing vesicle release (Furness et al., 2013). Many of these EEC-secreting hormones, such as cholecystokinin (CCK) or glucagon-like peptide 1 (GLP-1), are essential in regulating satiation response and metabolism (Duca et al., 2021; Furness et al., 2013). In addition to the classic hormone secretion function, recent research has also demonstrated that the EECs form a basal membrane process called a ‘neuropod’ that directly synapses with the vagal sensory neurons (Bohorquez et al., 2015; Kaelberer et al., 2018). Through the EEC-vagal neuronal pathway, ingested nutrient information in the gut lumen can be transmitted to the brain (Kaelberer et al., 2018). Further studies have demonstrated that this nutrient-sensing EEC-vagal pathway is essential in driving the animal's food preference toward sugar and fat (Li et al., 2022; Tan et al., 2020; Buchanan et al., 2022). It is well known that the intestinal epithelium cells undergo significant remodeling during the postnatal period to adapt to the need for nutrient absorption and sensation (Ye and Rawls, 2021). Despite the importance of EECs in nutrient monitoring, gut-brain nutrient sensing, feeding behavior and systemic metabolic regulation, little is known about how environmental factors regulate EEC maturation and function during the postnatal developmental period.

Following birth, newborn babies are rapidly colonized by microbial organisms (Yatsunenko et al., 2012). These microbial organisms start to assemble the functional microbial community that plays important roles in the development of the infant (Yatsunenko et al., 2012). Previous studies have revealed that microbiota colonization during the early postnatal phase is crucial in promoting intestinal epithelium maturation and remodeling (Sprockett et al., 2018; Henning, 1981; Sommer and Backhed, 2013). Numerous pieces of evidence also suggest that gut microbiota are important in regulating nutrient absorption, metabolism and infant growth (Martinez-Guryn et al., 2018; Semova et al., 2012; Yang et al., 2016). Research from animal models and clinical studies suggest that gut microbiota are crucial in modulating feeding behavior, including appetite and food choice (Yu and Hsiao, 2021). However, little is known about how gut microbiota interact with EECs and regulate EEC function during development.

A major challenge in studying how environmental factors, such as gut microbiota, regulate EEC physiology has been the lack of *in vivo* techniques to study this rare intestinal epithelium population in the intact animal setting. Historically, these cells have been studied by measuring the circulating EEC-secreted hormones (Furness et al., 2013). However, many EEC hormones have very short half-lives, and the plasma hormone level does not mirror the EEC function nor the real-time EEC activity (Skibicka and Dickson, 2013). EEC activity has also been studied via cell culture or organoid culture systems. However, a cell or organoid culture is not able to mimic the dynamic and complex intestinal luminal factors that shape the EECs. It is also difficult to study how EECs communicate with neighboring cells or distant organs, such as the brain, using the *in vitro* culture system.

In this study, we used the zebrafish model to examine how commensal microbiota affect EEC maturation and function. Using an innovative approach to direct images and track the EEC cellular and mitochondrial Ca^2+^ activity in live zebrafish during development, our results revealed that EEC morphology, and cellular and mitochondrial activity is dynamically regulated during the EEC maturation process. Importantly, our results revealed that gut microbiota play crucial roles in promoting EEC maturation and mitochondrial function.

## RESULTS

### Immature EECs contain active filopodia structures at the basal lateral membrane

**Fig. 1.**
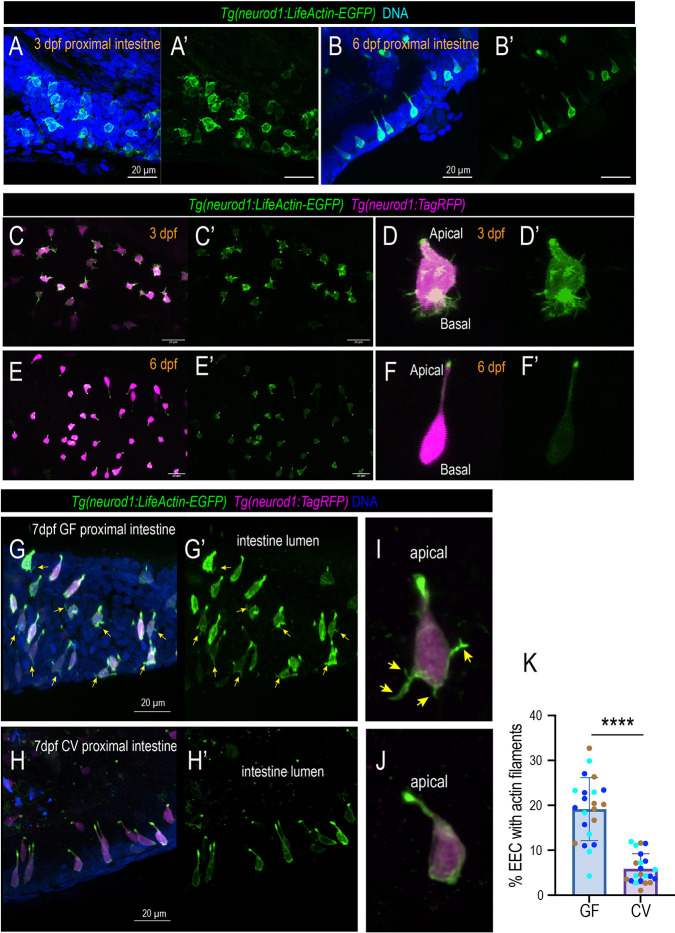
**EECs change morphology during development in a microbial-dependent manner.** (A-B′) Confocal projections of the *Tg(neurod1:lifeActin-EGFP)* 3 dpf and 6 dpf zebrafish proximal intestine. The EECs in the 3 dpf but not 6 dpf intestine exhibit thin actin filaments in the basal lateral membrane. (C,C′,E,E′) Live imaging traces EECs of the same zebrafish at 3 dpf (C) and 6 dpf (E). The EEC actin filaments are labeled via the *Tg(neurod1:lifeActin-EGFP)*. (D,D′,F,F′) Magnified view showing a typical 3 dpf EEC and 6 dpf EEC. Note that at 3 dpf, active actin filaments are observed at the basal lateral membrane. At 6 dpf, the actin filaments are only enriched in the apical brush border. (G-H′) Confocal projection of the 7 dpf *Tg(neurod1:lifeActin-EGFP); Tg(neurod1:TagRFP)* GF and CV zebrafish proximal intestine. Yellow arrows indicate the EECs that contain actin filaments labeled by *neurod1:lifeActin-EGFP*. (I,J) Representative EECs in 7 dpf GF and CV zebrafish proximal intestine. Yellow arrows indicate the presence of actin filament protrusions at the GF EEC base that are labeled by *lifeActin-EGFP* but not by *TagRFP*. (K) Quantification of the percentage of EECs with actin filaments in GF and CV conditions. Each dot represents an individual zebrafish. Zebrafish samples were pooled from three independent derivation experiments (samples from one derivation experiment are labeled by the same color): 2597 EECs were analyzed in CV and 2043 EECs were analyzed in GF. Data are mean±s.d. ****P<0.0001 (unpaired, two-tailed Student's *t*-test). Scale bars: 20 μm.

The zebrafish EECs start to form at ∼60 h post fertilization (hpf). At ∼3 days post fertilization (dpf), the zebrafish hatch from their chorion. At this point, the gut lumen opens and gut microbiota start to colonize the intestine. Similar to mammalian system, the zebrafish proximal intestine is the major site that is responsible for nutrient sensing and absorption (Ye et al., 2019). Using a *Tg(neurod1:lifeActin-EGFP)* transgenic zebrafish model developed in our previous study (Ye et al., 2019), we examined the EEC actin filament dynamics in 3 dpf and 6 dpf zebrafish proximal intestine. Previous studies have demonstrated that the intestinal epithelium cells, including EECs, are highly polarized and contain a dense actin network in the microvilli at the apical brush border (Ye et al., 2019). To our surprise, our data showed that at 3 dpf, almost all of the zebrafish EECs exhibit complex actin filament protrusions at the base (Fig. 1A,A′). Interestingly, we did not detect active basal actin filaments in other IECs at 3 dpf zebrafish (Movie 1). This indicates that the formation of the basal actin filaments is not associated with the general intestinal epithelium development process. It is a unique phenomenon that involves immature EECs. By 6 dpf, the EEC basal actin filaments disappeared. Most EECs exhibited typical spindle-type morphology with a flat base (Fig. 1B,B′). To further examine the EEC actin filament dynamic change, we performed live imaging of the EECs at 3 dpf *Tg(neurod1:lifeActin-EGFP)* in zebrafish and traced the same fish to 6 dpf. Consistently, we observed that at 3 dpf, almost all the EECs have complex actin filopodium filaments in the basal lateral portion, and the EECs extend and retract their filopodium filaments constantly (Fig. 1C-D′; Movie 2). However, in the same zebrafish, by 6 dpf the EECs do not have actin filaments at the base but contain a high LifeActin-EGFP signal at the apical brush border (Fig. 1E-F′; Movie 3). Like the proximal intestine, EECs in the distal intestine also exhibit high actin filaments at 3 dpf, and the EEC actin filaments disappeared by 6 dpf (Fig. S1A-B′). To examine whether EEC maturation displayed a proximal-to-distal progression pattern, the percentage of EECs that displayed active actin filaments in the proximal and distal intestine in 4 dpf zebrafish was compared. Our data revealed that at 4 dpf, a similar percentage of EECs displayed active actin filaments between proximal and distal intestine (Fig. S1C-F), suggesting that the proximal and distal intestinal EECs may mature simultaneously. Together, our data revealed for the first time that immature EECs have an active filopodia process at the basal lateral membrane. When the EECs start to develop and mature, they become more polarized and lose their basal filopodia process. In general, filopodia are antennae for cells to probe their environment (Mattila and Lappalainen, 2008). The function of the immature EEC filopodia and the molecular mechanisms that regulate the EEC filopodia formation require further investigation.

We investigated whether gut microbiota regulate the EEC filopodia process and maturation via the zebrafish gnotobiotic approach (Pham et al., 2008). *Tg(neurod1:lifeActin-EGFP)* zebrafish were derived as germ-free (GF) at 0 dpf. At 3 dpf, the zebrafish were maintained as GF or colonized with commensal microbiota (Conventionalized, CV). The gnotobiotic zebrafish were fed from 3 dpf to 7 dpf, and the zebrafish were fixed at 7 dpf. The GF and CV zebrafish proximal intestines were imaged. Our results demonstrated that ∼20% of 7 dpf GF zebrafish EECs in the proximal intestine still have filopodia actin filaments at the base (Fig. 1G,G′,I,K; Fig. S2A-C). The percentage of EECs with actin filaments is significantly reduced in CV zebrafish (Fig. 1H,H′,J,K; Fig. S2D-E). This suggests that certain microbial cues promote EEC maturation and facilitate EEC actin remodeling. To further confirm our findings, GF and CV *Tg(neurod1:lifeActin-EGFP)* zebrafish were generated, and live confocal imaging was performed on these samples at 7 dpf. Consistently, our results suggest that a higher percentage of GF zebrafish display an active filopodia-like actin filament process (Fig. S2F-J; Movie 4). The percentage of EECs with actin filaments remained significantly higher in 9 dpf GF zebrafish (Fig. S3). These data suggest that gut microbiota are required to promote EEC actin remodeling and maturation.

Previous mice studies have suggested that EECs form an extended membrane process called a ‘neuropod’ to connect with the nervous system (Bohorquez et al., 2015; Reinshagen, 2019). Interestingly, we observed that some EECs in 7 dpf or 9 dpf zebrafish intestines formed an extended membrane process in the basal membrane that morphologically resembles the mammalian neuropod of EECs (Figs S3D, S4C-D′). The extended membrane process is distinct from the thin actin filopodia filaments, which are only labeled by LifeActin-EGFP but not by RFP (Fig. 1C-D′,G,I; Figs S2B,C,F-H and S3D-D″. The neuropods are not detected in 3 dpf zebrafish EECs. The neuropod-like EECs are rare in the 7 dpf GF zebrafish intestine (Fig. S4A-B′). The CV zebrafish exhibit a significantly higher percentage of neuropod-like EECs in the intestine (Fig. S4E). Our data suggest that the formation of the neuropod-like structures in mature EECs may require certain microbial cues.

### Gut microbiota alter EEC subtypes

Previous studies, including ours, have demonstrated that, similar to mammals, the zebrafish EECs have diverse subtypes (Ye et al., 2019; Lavergne et al., 2020). A recent zebrafish intestine epithelium single-cell (sc) RNA-sequencing (RNA-seq) dataset further revealed the five EEC subtypes in the zebrafish larvae characterized by their distinct hormone expression profiles (Fig. S5A,B) (Wen et al., 2021). EEC1 is characterized by the expression of the hormonal genes *peptide YY* (*pyyb*), *somatostatin 2* (*sst2*), and *ghrelin* (*ghrl*) (Fig. S5A,B). EEC2 expresses the hormonal genes *preproglucagon a* (*gcga*), the gene that encodes Glucagon and Glucagon-like peptide 1 (GLP-1), *vasoactive intestinal polypeptide b* (*vipb*) and *insulin-like 5a* (*insl5a*) (Fig. S5A,B). EEC3 expresses the hormonal genes *calcitonin related polypeptide alpha* (*calca*) and *neuromedin Bb* (*nmbb*) (Fig. S5A,B). EEC4 expresses the hormonal gene *cholecystokinin* a (*ccka*) (Fig. S5A,B). EEC5 uniquely expresses the following hormonal genes: *brain-derived neurotrophic factor* (*bdnf*), *adenylate cyclase-activating peptide-1a* (*adcyap1a*), *preproenkephalin a* (*penka*) and *tryptophan hydroxylase 1b* (*tph1b*), the enzyme that synthesizes serotonin (Fig. S5A,B). The EEC5 also highly and uniquely expresses *transient receptor potential ankyrin 1b* (*trpa1b*). The Trpa1^+^ EECs that are characterized in our previous studies sense microbial stimulants and are crucial in regulating gut motility and intestinal homeostasis (Ye et al., 2021). Some of these EEC markers were labeled via immunofluorescence staining and transgenic approaches (Fig. S5C-K). Our results confirmed the hormonal expression profiles in different EEC subtypes that were revealed in the scRNA-seq above (Fig. S5C-K). Moreover, consistent with previous studies (Ye et al., 2019), our data revealed that the distribution of the EEC subtypes exhibits regional specificity (Fig. S5C-K). For example, the PYY^+^ EECs were exclusively in the proximal intestine, whereas the Trpa1^+^ EECs were distributed along the whole digestive tract. Interestingly, the Trpa1^+^ EECs (EEC5) appeared to have heterogeneity, as the proximal intestinal Trpa1^+^ EECs expressed both Enk and Serotonin (Fig. S5C-K); however, the middle and distal intestinal Trpa1^+^ EECs did not express ENK, only express serotonin (Fig. S5C-K).

**Fig. 2.**
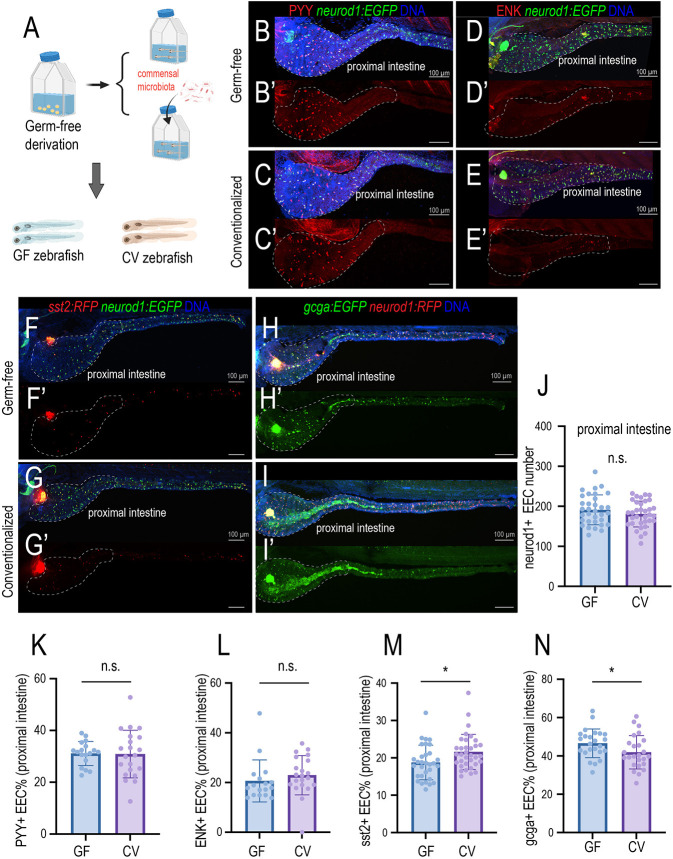
**Gut microbiota modulate EEC subtypes.** (A) Gnotobiotic zebrafish experimental procedure to examine the effects of gut microbiota on EEC subtype formation. (B-C′) Confocal projection of representative 7 dpf GF and CV zebrafish showing the PYY^+^ EECs (red). The total EECs were labeled by the *Tg(neurod1:EGFP)* transgene (green). (D-E′) Confocal projection of representative GF and CV zebrafish intestine at 7 dpf showing the ENK^+^ EECs (red). (F-G′) Confocal projection of representative GF and CV zebrafish intestine showing the *sst2:RFP*^+^ EECs in the intestine. (H-I′) Confocal projection of representative GF and CV zebrafish intestine showing the *gcga:EGFP*^+^ EECs in the intestine. Dashed white outlines in B-I′ indicate the proximal zebrafish intestine (intestinal bulb). (J-N) Quantification of the total number of EECs (J), percentage of PYY^+^ EECs (K), percentage of ENK^+^ EECs (L), percentage of *sst2*^+^ EECs (M) and percentage of *gcga*^+^ EECs in 7 dpf GF and CV zebrafish proximal intestine. Each dot represents an individual zebrafish. Samples were pooled from three derivation experiments (J,M,N) and one derivation experiment (K,L). Data are mean±s.d. *P<0.05 (unpaired, two-tailed Student's *t*-test). n.s., not significant. Scale bars: 100 μm.

Next, we investigated how commensal gut microbiota affect EEC subtype specification (Fig. 2A). We focused on the EEC subtypes that are present in the proximal intestine. Previous studies have demonstrated that both immature and mature EECs express *Neurod1* (Gehart et al., 2019). Consistently, our results showed that the *neurod1:EGFP* fluorescence level does not change between 3 dpf EECs and 6 dpf EECs, suggesting that the expression of *neurod1* is not altered by the EEC maturation state (Fig. S6A-C). Our results revealed that commensal microbiota colonization did not alter the total Neurod1^+^ EEC cell number in the proximal intestine (Fig. 2B,J). The percentage of PYY^+^ EECs and ENK^+^ EECs in the proximal intestine also did not alter upon gut microbiota colonization (Fig. 2B-E′,K,L). However, commensal microbiota colonization increased the percentage of Sst2^+^ EECs but decreased the percentage of Gcga^+^ EECs in the proximal intestine (Fig. 2F-I′,M,N). In the middle and distal intestine, the total number of EECs labeled by *Tg(neurod1:EGFP)* was not changed between GF and CV zebrafish (Fig. S6D). However, GF zebrafish displayed a higher number of Sst2^+^ EECs, Gcga^+^ EECs, and Trpa1^+^ EECs in the middle and distal intestine (Fig. S6E-G). Together, our data suggest that commensal microbiota colonization may alter EEC subtype specification.

### Gut microbiota promote EEC maturation and mitochondrial function

**Fig. 3.**
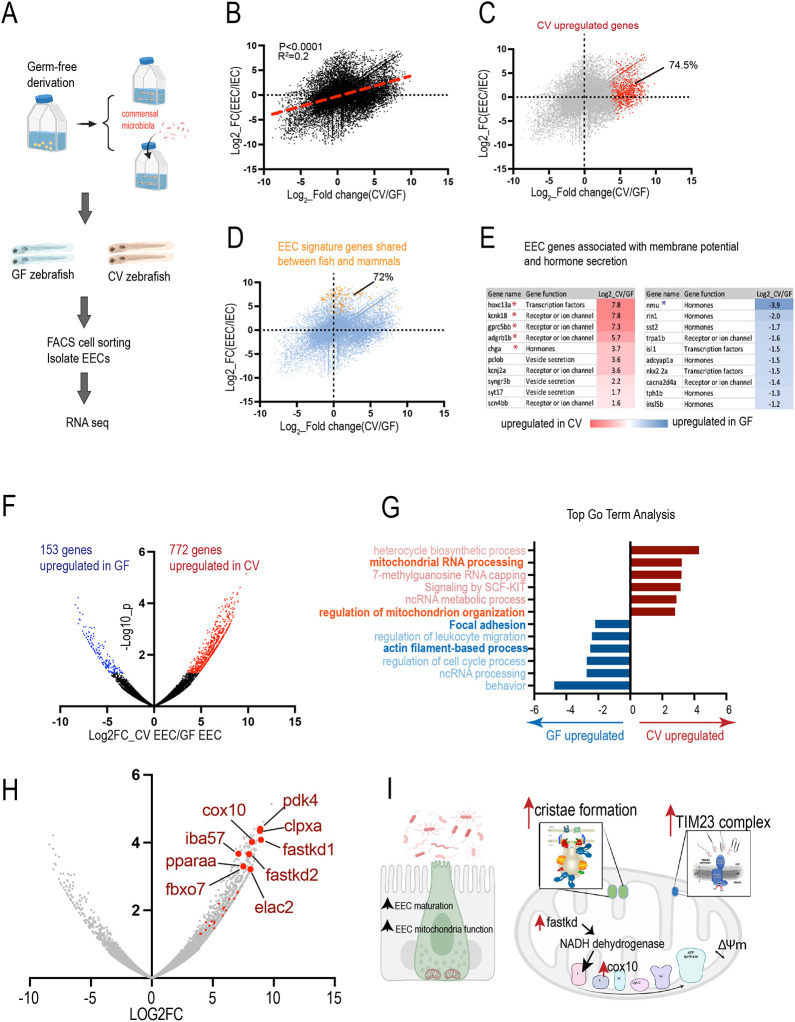
**Gut microbiota promote EEC maturation and mitochondrial function.** (A) Experimental overview for transcriptomic analysis of the FACS-sorted EECs from 8 dpf *Tg(neurod1:RFP); Tg(cldn15la:EGFP)* GF and CV zebrafish. *Tg(cldn15la:EGFP)* labels the intestine epithelium and *Tg(neurod1:RFP)* labels the EECs in the intestinal epithelium. Cells with both GFP and RFP fluorescence were sorted. (B) Positive correlation between the genes that are upregulated in CV (*x*-axis) and the genes that are enriched in EECs (*y*-axis). (C) Among the genes that are significantly upregulated in CV (red), 74.5% are enriched in the EECs. (D) Of the EECs signature genes shared between zebrafish and mammals, 72% are upregulated in CV. (E) Differential expression of the EEC signature genes that encode hormone peptides or are involved in membrane potential in GF and CV conditions. Asterisks indicate that the genes are significantly upregulated in the GF or CV conditions. (F) Volcano plot showing the genes that are significantly upregulated in CV or GF. (G) GO term analysis of the CV or GF upregulated genes. (H) Volcano plot showing the genes that are involved in mitochondrial function. Many of the genes that are associated with mitochondrial regulation are among the most significantly upregulated genes in CV EECs. (I) Model figure showing that commensal microbiota colonization promotes EEC maturation and mitochondrial function.

To further understand how commensal microbiota modulate EECs in zebrafish, transcriptomic analysis of the fluorescence-activated cell sorting (FACS)-sorted EECs from 8 dpf *Tg(neurod1:RFP); Tg(cldn15la:EGFP)* GF and CV zebrafish was conducted (Fig. 3A). *Tg(cldn15la:EGFP)* labels the intestine epithelium and *Tg(neurod1:RFP)* labels the EECs in the intestinal epithelium. The cells with GFP and RFP double fluorescence were sorted and confirmed as EECs (Ye et al., 2021). For each gene, the fold change in response to gut microbial status (CV versus GF) and fold change in response to the cell fate (EEC versus other IEC) was plotted (Fig. 3B). Our results demonstrated that there is a weak but significant positive correlation between genes that are enriched in EECs and the genes that are upregulated in the CV condition (Fig. 3B). Within the genes that are significantly upregulated in CV, ∼74.5% of them were enriched in the EECs (Fig. 3C). We then plotted the conserved EEC signature genes that are shared among zebrafish, mice and humans (Ye et al., 2021; Table S1). Our results indicate that ∼72% of those conserved EEC signature genes are upregulated in CV (Fig. 3D; Table S1). Within those conserved EEC genes, many of them are associated with EEC cell membrane potential regulation and vesicle secretion (Fig. 3E). The CV condition also significantly upregulated the chromogranin A gene (*chga*), which labels the mature EECs (Fig. 3E) (Facer et al., 1985). Therefore, consistent with our findings in Fig. 1, the transcriptomic analysis indicates that gut microbiota may promote EEC cell maturation. Next, we performed Gene Ontology (GO) term analysis of the genes that are significantly upregulated in CV and the genes that are significantly upregulated in GF using the Metascape gene analysis tool (Fig. 3F,G). In the genes that are significantly upregulated in GF EECs, the top GO term included gene functions related to adhesion, migration and actin filament-based processes (Fig. 3G). Consistent with the data presented in Fig. 1, the results from EEC RNA-seq suggest that gut microbiota regulate EEC actin dynamics and maturation.

Interestingly, within the CV upregulated genes, several GO terms associated with mitochondrial function were enriched (Fig. 3G). We then annotated all of the genes associated with different aspects of mitochondrial function (Table S1). We found that almost all of the genes in the FASTK mitochondrial RNA binding family, TIM23 complex, and mitochondrial contact site and cristae organizing system were upregulated in CV (Table S1). The mitochondrial DNA encodes 13 proteins that are crucial for electron transport chain reactions (Taanman, 1999). The FAS-activated serine/threonine kinase family (FASTK) is located in the mitochondrial matrix and plays an important role in processing RNA transcribed from the mitochondrial DNA (Jourdain et al., 2017). The FASTK gene family is essential for synthesizing the components of the electron transport chain (Jourdain et al., 2017). Within the FASTK family, previous studies have shown that FASTK and FASTKD2 increase the NADH dehydrogenase transcripts and promote mitochondrial respiration specifically (Jourdain et al., 2015, 2017). *fastkd2* is one of the most significant genes upregulated in CV EECs (Fig. 3H). Mitochondria acquire most of their protein from the cytosol (Sim et al., 2023). The TIM23 complex is essential for translocating cytosolic preprotein into the mitochondrial matrix across the mitochondrial membrane (Sim et al., 2023). Within the mitochondria, the inner membrane forms invaginations known as cristae. The cristae are very specialized structures that support respiration (Kondadi et al., 2020). Our results indicate that many genes that are associated with cristae organization are upregulated in CV EECs (Table S1), suggesting that commensal microbiota colonization increases EEC mitochondrial respiration function. In addition to the genetic pathways above, our results also indicate that *cox10* (an important component of mitochondrial respiration for complex III) is among the most significantly upregulated CV EEC genes (Fig. 3H). Together, our transcriptomic data indicate that gut microbiota promote EEC maturation and mitochondrial function by increasing different genetic pathways that are involved in mitochondrial respiration (Fig. 3I).

### Commensal microbiota promote the formation of mitochondria hotspots in the EEC basal membrane

**Fig. 4.**
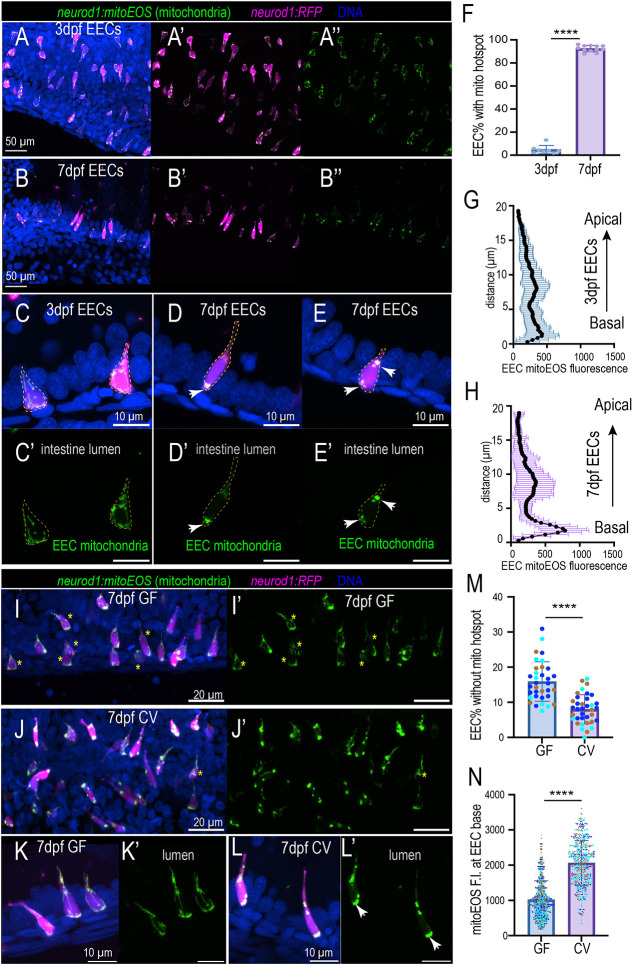
**Commensal microbiota colonization promotes mitochondria accumulation at the EEC basal lateral membrane.** (A-B″) Confocal projections of representative 3 dpf and 7 dpf zebrafish proximal intestine. The EECs were labeled via the *neurod1:RFP* transgene, and the EEC mitochondria were labeled via the *neurod1:mitoEOS* transgene. Mitochondria in 7 dpf EECs but not in 3 dpf EECs display a punctate distribution pattern. (C-E′) Magnification showing representative EECs in 3 dpf and 7 dpf zebrafish proximal intestine. Mitochondria are evenly distributed in 3 dpf but not 7 dpf EEC cytoplasm. White arrows indicate mitochondria hotspots at the EEC base and neck. Dashed yellow outlines indicate individual EECs. (F) Quantification of percentage of EECs displaying mitochondria hotspot pattern in 3 dpf and 7 dpf zebrafish proximal intestine. Each dot represents an individual zebrafish. (G,H) Quantification of mitochondrial distribution profiles in 3 dpf (G) and 7 dpf (H) zebrafish proximal intestinal EECs. Compared with 3 dpf zebrafish EECs, 7 dpf zebrafish EECs display higher mitochondrial contents at the EEC base: 36 EECs from five 3 dpf zebrafish and 36 EECs from five 7 dpf zebrafish were analyzed. (I-J′) Confocal projections of representative 7 dpf *Tg(neurod1:mitoEOS); Tg(neurod1:RFP)* GF and CV zebrafish proximal intestine. Asterisks indicate the EECs that display an even mitochondrial hotspot distribution pattern. (K-L′) Magnification showing representative EECs in 7 dpf GF and CV zebrafish proximal intestine. Mitochondria were evenly distributed within the GF EEC cytoplasm but displayed a hotspot pattern in CV EECs. White arrows indicate the mitochondrial hotspot at the EEC base. (M) Quantification of the percentage of EECs without basal mitochondrial hotspots in 7 dpf GF and CV zebrafish proximal intestine. Each dot represents an individual zebrafish. Zebrafish samples pooled from three independent derivation experiments were analyzed (samples from one derivation experiment are labeled by the same color): 3338 EECs from 36 CV zebrafish and 2697 EECs from 34 GF zebrafish were analyzed. (N) Quantification of the mitochondrial fluorescence intensity at the basal membrane in 7 dpf GF and CV zebrafish proximal intestine: 15 zebrafish from three independent derivation experiments were analyzed in each group, and >120 EECs from each zebrafish were analyzed. Each dot represents an individual EEC. The EECs from the same derivation experiments are labeled with the same color. Data are mean±s.d. ****P<0.0001 (unpaired, two-tailed Student's *t*-test). Scale bars: 50 μm (A,B); 10 μm (C-E′,K-L′); 20 μm (I-J′).

Next, we investigated how gut microbiota regulate EEC mitochondria. We used a *Tg(neurod1:mitoEOS)* transgenic zebrafish model to visualize the EEC mitochondria (Mandal et al., 2018). In this model, the green fluorescent Eos protein contains a mitochondrial tag that is expressed in EECs to label their mitochondria. Using this model, we analyzed the EEC mitochondrial abundance and intracellular mitochondrial distribution. Our results revealed that the mitochondrial abundance in the proximal intestinal EEC did not alter between 3 dpf and 5 dpf (Fig. S7A). However, at 6 dpf, EECs in the proximal intestine exhibited higher mitochondrial abundance compared with 3-5 dpf EECs (Fig. S7A). Interestingly, at 3 dpf, the mitochondria were evenly distributed within the EECs (Fig. 4A-A″,C,C′,G; Fig. S8A,B). At 7 dpf, most EECs exhibited hotspot mitochondrial distribution patterns (Fig. 4B-B″,D-F,H; Fig. S8C-D). High mitochondrial contents are found at the base of EECs, presumably at the sites where EECs secrete vesicles (Fig. 4D-E′,H; Fig. S8C-D). In addition to the EEC base, the mitochondria hotspot is also detected in the EEC neck (Fig. 4E,E′). To further confirm EEC changes in mitochondria distribution during maturation, we traced the same zebrafish from 3 dpf to 6 dpf and imaged the proximal intestinal EEC mitochondria *in vivo*. Consistently, our results demonstrated that EEC mitochondria switch to a ‘hotspot’ distribution pattern at 6 dpf and increase localization at the base (Fig. S9A-G). When we compared GF and CV zebrafish, commensal microbiota colonization did not increase the proximal intestinal EEC mitochondrial abundance (Fig. S7B). However, the commensal microbiota colonization promoted the formation of mitochondrial hotspots at the basal membrane (Fig. 4I-M), and the CV zebrafish had higher mitochondrial contents near the basal membrane (Fig. 4I-L,N). To further confirm our findings, we generated 7 dpf GF and CV zebrafish and performed live imaging. Consistent with our analysis using fixed tissue, *in vivo* imaging results also demonstrated that commensal microbiota colonization promoted EEC mitochondrial hotspot formation and mitochondria localization at the EEC base (Fig. S9H-K).

### Mature EECs increase mitochondrial activity

**Fig. 5.**
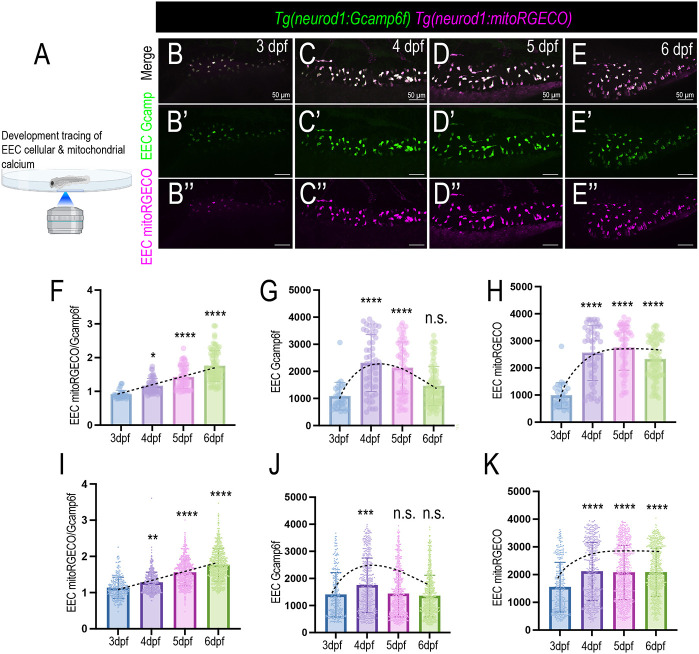
**EEC mitochondrial activity changes during development.** (A) Schematic showing *in vivo* imaging to trace the EEC cytoplasmic and mitochondrial Ca^2+^ in the same zebrafish from 3 dpf to 6 dpf. The commensal microbiota colonizing conventionally raised zebrafish was used. EECs from nine zebrafish were traced and analyzed. (B-E″) Confocal projections of the same *Tg(neurod1:Gcamp6f); Tg(neurod1:mitoRGECO)* zebrafish at 3 dpf, 4 dpf, 5 dpf, and 6 dpf. The EEC cytoplasmic Ca^2+^ level is represented via the Gcamp6f fluorescence (green). The EEC mitochondrial Ca^2+^ level is displayed through mitoRGECO fluorescence (magenta). (F-H) EECs from the same zebrafish were analyzed from 3 dpf to 6 dpf. EEC mitochondria-to-cytoplasmic Ca^2+^ ratio, cytoplasmic Ca^2+^ and mitochondrial Ca^2+^ were quantified. (I-K) Pooled EECs from nine zebrafish were analyzed from 3 dpf to 6 dpf. Mitochondria-to-cytoplasmic Ca^2+^ ratio, cytoplasmic Ca^2+^ and mitochondrial Ca^2+^ were quantified. Each dot in F-K represents an individual EEC. Dashed lines indicate the changes of EEC cellular and mitochondrial calcium activity from 3 dpf to 7 dpf. Data are mean±s.d. *P<0.05, **P<0.01, ***P<0.001, ****P<0.0001 (one-way Anova followed by Tukey's post test). Scale bars: 50 μm.

To analyze the dynamic change of EEC cellular and mitochondrial activity during their development and maturation process, we used the *Tg(neurod1:Gcamp6f); Tg(neurod1:mito-RGECO)* dual transgenic models that were generated and used by previous studies (Mandal et al., 2018; Rupprecht et al., 2016). In this model, the green fluorescent Ca^2+^ indicator protein Gcamp6f is expressed in the EEC cytoplasm. A red fluorescent Ca^2+^ indicator protein RGECO that contains a mitochondrial tag is expressed in the EEC mitochondrial matrix. Therefore, by using this dual transgenic model, we can simultaneously measure EEC cytoplasmic Ca^2+^ levels and mitochondrial Ca^2+^ levels by measuring the change in green and red fluorescence (Movies 5 and 6). To confirm *Tg(neurod1:mitoRGECO)* measures EEC mitochondrial Ca^2+^, carbonyl cyanide-p-trifluoromethoxyphenylhydrazone (FCCP), which disrupts mitochondrial inner membrane potential (Benz and McLaughlin, 1983), was used. Our result shows that a decreased mitoRGECO fluorescence in EECs was recorded in FCCP-treated zebrafish (Fig. S10). We then analyzed how EEC cytoplasmic and mitochondrial Ca^2+^ levels changed during development by tracing the same zebrafish from 3-6 dpf (Fig. 5A-E″). Our results showed that, at 3 dpf, the EECs exhibited low cytoplasmic and mitochondrial Ca^2+^ levels (Fig. 5B-B″,G,H). However, at 4 dpf, there was a significant increase in both EEC cytoplasmic and mitochondrial Ca^2+^ levels (Fig. 5C-C″,G,H). From 5-6 dpf, the EEC cytoplasmic Ca^2+^ levels decreased, whereas mitochondrial Ca^2+^ levels remained high (Fig. 5D-E″,G,H). As a result, from 3 dpf to 6 dpf, the EEC mitochondrial-to-cytoplasmic Ca^2+^ ratio continued to increase (Fig. 5F). When we grouped data of nine zebrafish of the same age together, we also observed that, as the EECs became more mature, the EECs increased their mitochondrial-to-cytoplasmic Ca^2+^ level ratio (Fig. 5I-K).

### Gut microbiota increase resting EEC mitochondrial activity and spontaneous firing

**Fig. 6.**
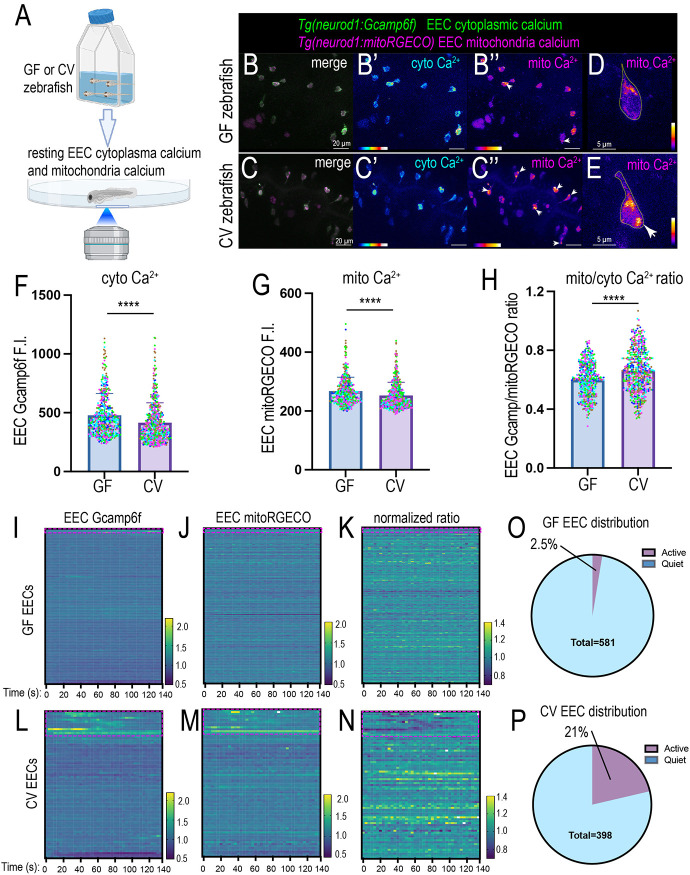
**Commensal microbiota colonization alters the resting EEC cytoplasm and mitochondria Ca^2+^ activity.** (A) Schematic showing *in vivo* imaging of EEC cellular and mitochondrial Ca^2+^ activity in live zebrafish. (B-C″) Confocal projection of 7 dpf GF and CV *Tg(neurod1:Gcamp6f); Tg(neurod1:mitoRGECO)* zebrafish proximal intestine. The white arrowheads in B″ and C″ indicate the EECs with higher mitochondrial activity near the base membrane. (D,E) Representative GF and CV zebrafish EECs in the proximal intestine. The white arrow in E indicates high mitochondrial Ca^2+^ near the base membrane. (F-H) Quantification of absolute Gcamp (F), mitoRGECO (G) and mitoRGECO/Gcamp ratio (H) in 7 dpf GF and CV zebrafish proximal intestinal EECs. Each dot represents an EEC: 523 EECs from four GF zebrafish and 575 EECs from five CV zebrafish were analyzed. EECs from the same GF or CV zebrafish are labeled with the same color. Three independent derivation experiments were performed, and the same trend was observed. (I-N) Analysis of the relative EEC Gcamp (I,L), EEC mitoRGECO (J,M) and EEC mitoRGECO/Gcamp ratio (K,N) in GF (I-K) and CV (L-N) zebrafish on a temporal scale. The EEC Gcamp, EEC mitoRGECO and EEC mitoRGECO/Gcamp ratio at each time point were normalized to t0. Each line represents an individual EEC. The red boxed areas indicate the EECs that exhibit dynamic Gcamp fluorescence fluctuation, referred to as active EECs in O and P. (O,P) Quantification of the percentage of the quiet and active EECs in GF and CV zebrafish: 581 EECs from four GF zebrafish and 398 EECs from five CV zebrafish were used for the analysis in I-P. A second independent derivation experiment with five GF zebrafish and five CV zebrafish was performed, and the same conclusion was reached. Data are mean±s.d. ****P<0.0001 (unpaired, two-tailed Student's *t*-test). Scale bars: 20 µm (B-C″); 5 µm (D,E).

Our new genetic zebrafish model and imaging approaches allowed us to investigate how gut microbiota change EEC cytoplasmic and mitochondrial activity *in vivo*. We generated *Tg(neurod1:Gcamp6f); Tg(neurod1:mitoRGECO)* GF and CV zebrafish and imaged the proximal intestinal EECs at 7 dpf (Fig. 6A). First, we examined the absolute cytoplasmic Ca^2+^ levels and EEC mitochondrial Ca^2+^ levels in GF and CV zebrafish proximal intestinal EECs. Compared with GF EECs, CV EECs exhibited significantly lower cytoplasmic and mitochondrial Ca^2+^ levels (Fig. 6B-G). However, the CV EECs exhibited a significantly higher mitochondrial to cytoplasmic Ca^2+^ ratio (Fig. 6H). Moreover, many of the EECs in the CV but not GF zebrafish exhibited higher mitochondrial Ca^2+^ levels near the basal membrane (Fig. 6B″,C″,D,E). These results suggest that gut microbiota may promote low resting EEC cytoplasmic Ca^2+^ levels but enhance EEC mitochondrial activity, consistent with the results from the RNA-seq analysis above (Fig. 2).

Using a 3D cell tracking approach, we automatically tracked individual EECs and analyzed their Gcamp6f and mitoRGECO fluorescent change on a temporal scale (Fig. S11; Movies 7,8). We analyzed the change in relative EEC cytoplasmic Ca^2+^ and mitochondrial Ca^2+^ levels in GF and CV zebrafish. For each EEC, we normalized the EEC Gcamp6f, EEC mitoRGECO and EEC mitoRGECO/Gcamp6f ratio values to their values at time 0. Our results indicate that some EECs in the CV zebrafish exhibited low amplitude firing as reflected by the temporal fluctuation of the EEC cytoplasmic Ca^2+^ levels (Fig. 6L-N; Movie 9). However, this spontaneous firing was not apparent in the GF zebrafish EECs (Fig. 6I-K; Movie 10). Analysis of EECs across different GF and CV zebrafish samples indicated that ∼21% of CV EECs exhibited low amplitude firing compared with only 2.5% of GF EECs (Fig. 6O,P). Those EECs with spontaneous firing increased the relative mitochondrial Ca^2+^ levels but not the relative mitochondria-to-cytoplasm Ca^2+^ ratio (Fig. 6M,N). These results suggest that at the resting condition, the CV EECs exhibit more dynamic cytoplasmic and mitochondrial Ca^2+^ activity change in the resting state.

### Nutrient-induced EEC mitochondrial Ca^2+^ increase requires gut microbiota

**Fig. 7.**
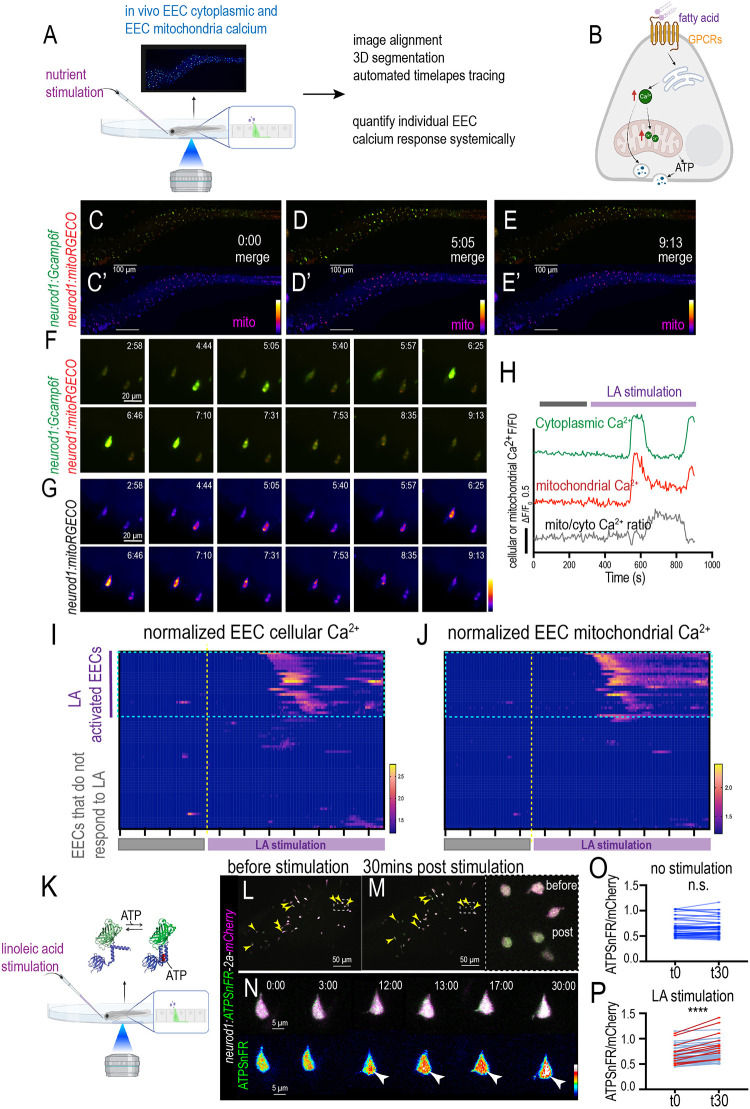
**Analysis of individual EEC cellular and mitochondrial activity in response to nutrient stimulation in live zebrafish.** (A) Schematic showing *in vivo* imaging of EEC cellular and mitochondrial Ca^2+^ activity in response to nutrient stimulation. (B) The hypothesis model figure shows that fatty acid increases both cytoplasmic and mitochondrial Ca^2+^, which powers the hormone vesicle secretion from EECs. (C-E′) Time-lapse images of the whole zebrafish intestinal EEC cytoplasmic and mitochondrial Ca^2+^ change post linoleic acid stimulation. The EEC cytoplasmic Ca^2+^ was labeled by Gcamp6f (green) and the EEC mitochondrial Ca^2+^ was labeled by mitoRGECO (magenta). (F,G) Magnification shows two representative EECs that are activated by linoleic acid. (H) Analysis of fluorescence change of Gcamp, mitoRGECO and mitoRGECO/Gcamp ratio in a representative linoleic acid-activated EEC. (I,J) Analysis of fluorescence change of Gcamp and mitoRGECO of 68 EECs in one zebrafish before and after linoleic acid stimulation. The EECs that increase cytoplasmic Ca^2+^ were defined as ‘LA-activated EECs’. Most of the LA-activated EECs also exhibited increased mitochondrial Ca^2+^. (K) Schematic showing measurement of EEC ATP concentration using zebrafish injected with *neurod1:ATPSnFR-2a-mCherry* plasmid. The ATPSnFR/mCherry ratio is used to measure the ATP concentration within EECs. (L,M) Confocal projection of the zebrafish intestine before and 30 min after linoleic acid stimulation. The yellow arrowheads indicate the EECs that exhibit a significant increase in the ATPSnFR/mCherry ratio upon linoleic acid stimulation. (N) Time-lapse imaging of a representative EEC that increased ATPSnFR/mCherry ratio after linoleic acid stimulation. White arrowheads indicate, at 12, 13, 17 and 30 min post linoleic acid stimulation, a representative EEC displaying an increased ATPSnFR fluorescence level. (O-P) The ATPSnFR/mCherry ratio of the EECs at 0 min (t0) and 30 min (t30) in unstimulated (O) and linoleic acid-stimulated (P) zebrafish. The red lines in P indicate the EECs that increased the ATPSnFR/mCherry ratio by more than 15%. More than 100 EECs from three zebrafish were analyzed for K-P. ****P<0.0001 (paired, two-tailed Student's *t*-test). Scale bars: 100 μm (C-E′); 20 μm (F,G); 50 μm (L,M); 5 μm (N).

As the primary sensory cells, one of the major functions of EECs is to sense the nutrients in the intestinal lumen. To analyze how EECs respond to nutrients at the cellular level in live zebrafish, we developed a method to give stimulants to the zebrafish during confocal imaging. Using *in vivo* EEC Ca^2+^ imaging and 3D automated cell tracing, we measured the individual EEC cytoplasmic and mitochondrial Ca^2+^ response to nutrient stimulation systemically in live zebrafish (Fig. 7A; Movies 11,12). Our results demonstrated that nutrients such as linoleic acid stimulated a subset of EECs and increased the level of cytoplasmic Ca^2+^ (Fig. 7B-J; Fig. S12; Movies 11,12). Along with the increase in EEC cytoplasmic Ca^2+^, there was also a consistent increase in mitochondrial Ca^2+^ following nutrient stimulation (Fig. 7B-J; Fig. S12; Movies 11,12). In the linoleic acid-activated EECs, linoleic stimulation induced a cytoplasmic Ca^2+^ peak in these cells, and the cellular cytoplasmic Ca^2+^ levels returned to their basal activity level (Fig. 7F-H; Fig. S12). The mitochondrial Ca^2+^ levels increased immediately following the cytoplasmic Ca^2+^ peak (Fig. 7F-H; Fig. S12). However, unlike the cytoplasmic Ca^2+^, the mitochondrial Ca^2+^ level remained higher than the basal Ca^2+^ level after the peak (Fig. 7F-H; Fig. S12). As a result, the relative mitochondrial-to-cytoplasmic Ca^2+^ ratio increased post-linoleic acid stimulation (Fig. 7F-H; Fig. S12). In addition to linoleic acid, our results demonstrated that glucose also activated a subset of EECs (Fig. S13; Movie 13). Similarly, glucose activated both cytoplasmic and mitochondrial Ca^2+^ and increased the mitochondrial-to-cytoplasmic Ca^2+^ ratio (Fig. S13). Our results also demonstrated that the nutrient-induced mitochondrial Ca^2+^ increase was more prominent in the mitochondria near the basal membrane (Fig. S14). This suggests that the nutrient-induced mitochondrial Ca^2+^ increase is likely linked with the EEC vesicle secretion process. Fasting zebrafish and zebrafish fed with a normal diet exhibited similar nutrient-induced EEC cytoplasmic and mitochondrial Ca^2+^ activation (Fig. S15). In the conventionally raised zebrafish, the majority of the linoleic acid-activated EECs exhibited elevated mitochondrial Ca^2+^ in response to nutrient stimulation (Fig. 7I-J). Previous studies have demonstrated that increased mitochondrial Ca^2+^ promotes mitochondrial ATP production (Boyman et al., 2020; Jouaville et al., 1999). To confirm that elevated mitochondrial Ca^2+^ increases ATP, we injected the *neurod1:ATPSnFR-2a-mCherry* plasmid (Mandal et al., 2021) into the zebrafish. The ATPSnFR is a genetically encoded ATP sensor, and its green fluorescence intensity increases in an ATP concentration-dependent manner (Lobas et al., 2019). We stimulated the zebrafish that expressed the ATPSnFR-2a-mCherry transgene with linoleic acid and measured the EEC ATP production. Our results demonstrate that upon nutrient stimulation, a subset of EECs significantly increased intracellular ATP levels, which is reflected by the increased ATPSnFR/mCherry ratio (Fig. 7K-N,P). The increased EEC ATP production is not detected in the unstimulated zebrafish (Fig. 7O).

**Fig. 8.**
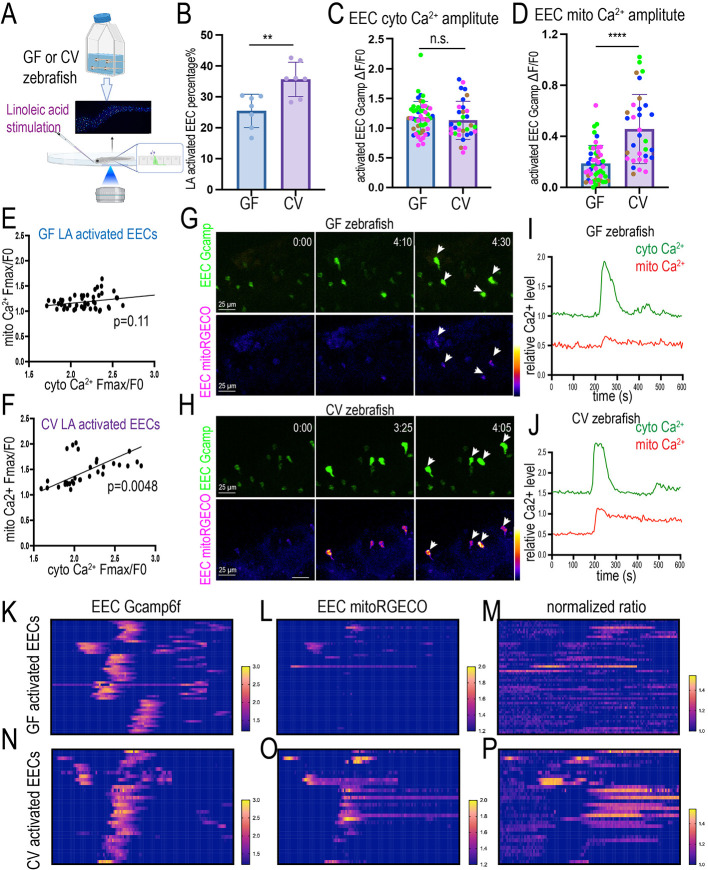
**Nutrient-induced EEC mitochondrial Ca^2+^ increase requires commensal microbiota colonization.** (A) Schematic showing *in vivo* imaging to analyze the 7 dpf GF and CV zebrafish EEC cytoplasm and mitochondrial activity in response to linoleic acid stimulation. (B) Quantification of linoleic acid-activated EEC percentage in GF and CV zebrafish. Each dot represents an individual zebrafish. (C,D) Quantification of the linoleic acid-activated EEC cytoplasmic Ca^2+^ amplitude (C) and the mitochondrial Ca^2+^ amplitude (D). Each dot represents an individual EEC. EECs from four GF and four CV zebrafish were analyzed. The EECs from the same GF or CV zebrafish are labeled with the same color. (E,F) Correlation between cytoplasmic Ca^2+^ amplification and mitochondrial Ca^2+^ amplification in GF (E) and CV (F) zebrafish EECs. (G,H) Time-lapse images of the representative EECs post linoleic acid stimulation in GF (G) and CV (H) zebrafish. The EEC cytoplasmic Ca^2+^ was labeled by Gcamp6f and the EEC mitochondrial Ca^2+^ was labeled by mitoRGECO. (I,J) Analysis of fluorescence change of Gcamp and mitoRGECO in representative linoleic acid-activated GF (I) and CV (J) EECs. (K-P) Analysis of the change of the EEC Gcamp6f fluorescence (K,N), EEC mitoRGECO fluorescence (L,O) and EEC mitoRGECO/Gcamp6f ratio (M,P) in GF and CV zebrafish. Only the linoleic acid-activated EECs were plotted. Analysis of 32 activated EECs from four CV zebrafish and 50 activated EECs from four GF zebrafish. A second independent derivation experiment was performed in which seven GF zebrafish and seven CV zebrafish were analyzed, and the same conclusion was reached for all data presented in this figure. Data are mean±s.d. **P<0.01, ****P<0.0001 (unpaired, two-tailed Student's *t*-test). n.s., not significant. Scale bars: 25 μm.

Finally, we investigated whether and how gut microbiota regulate EEC nutrient response. We generated GF and CV *Tg(neurod1:Gcamp6f); Tg(neurod1:mitoRGECO)* zebrafish (Fig. 8A). We then stimulated the GF and CV zebrafish with linoleic acid and recorded how the GF and CV EECs responded to the stimulation. Our results indicate that, compared with CV zebrafish, the percentage of EECs that can be activated by linoleic acid in GF zebrafish was reduced (Fig. 8B). Within the activated EECs, the cytoplasmic Ca^2+^ amplitude remained the same between GF and CV groups (Fig. 8C). However, within the activated EECs, the mitochondrial Ca^2+^ amplitude significantly increased in the CV EECs (Fig. 8D,G,H,J). The CV, but not GF, linoleic acid-activated EECs displayed a positive correlation between mitochondrial Ca^2+^ activation and cytoplasmic Ca^2+^ activation (Fig. 8E-J). The same result was shown when we traced the temporal EEC cytoplasmic and mitochondrial Ca^2+^ levels in GF and CV zebrafish (Fig. 8K-P). In most CV EECs, nutrient stimulation activated both cytoplasmic and mitochondrial Ca^2+^ and increased the mitochondrial-to-cytoplasmic Ca^2+^ ratio (Fig. 8N-P). However, the nutrient-induced EEC mitochondrial activation was significantly reduced in GF EECs (Fig. 8L,O). The nutrient-induced mitochondrial-to-cytoplasmic Ca^2+^ ratio increase was also impaired in GF EECs (Fig. 8M,P). These results suggest that the nutrient-induced EEC mitochondrial activation requires signals from commensal microbiota colonization.

## DISCUSSION

Using transcriptomics, genetics, *in vivo* imaging and gnotobiotic manipulation, this study revealed that commensal microbiota colonization is crucial in shaping EEC maturation and function during development (Fig. S16). Importantly, our data revealed that commensal microbiota colonization is essential in promoting mitochondrial activity and nutrient-induced mitochondrial activation in EECs. Selectively manipulating gut microbial signals to alter EEC mitochondrial function may open new opportunities to change EEC vesicle secretion and EEC–neuronal communication.

### EECs change mitochondrial activity during development

Mitochondria are the essential organelles that provide ATP to sustain cellular function, emerging as key players in coordinating cellular metabolism and cell differentiation, and regulating intestinal epithelium homeostasis (Khacho and Slack, 2017; Noguchi and Kasahara, 2018; Ludikhuize et al., 2020). Little is known about the physiological roles of mitochondrial function in EECs and how environmental factors regulate EEC mitochondrial activity. Using *in vivo* imaging to track EECs during development in live zebrafish, our results revealed that EEC mitochondrial activity is dynamically regulated during development. Our data showed that, shortly after commensal microbiota colonization, EECs increase both cytoplasmic and mitochondrial Ca^2+^ activity – a phenomenon we referred to as the ‘EEC awakening’. After the EEC awakening, the EECs downregulate their cytoplasmic Ca^2+^ levels but upregulate their mitochondrial-to-cytoplasmic Ca^2+^ ratio. As sensory cells, it is crucial for EECs to maintain low cytoplasmic Ca^2+^ levels to enable a depolarization potential. When EECs sense nutrient stimulants, the Ca^2+^ ion channel on the cell membrane or endoplasmic reticulum (ER) membrane will open (Furness et al., 2013). Ca^2+^ from the extracellular space or the ER lumen will flux into the cytoplasm matrix following the Ca^2+^ gradient. The low cytoplasmic Ca^2+^ levels are, therefore, essential to generating the gradient to produce the Ca^2+^ peak to trigger downstream cellular signaling events (Berridge et al., 2003). Maintaining the membrane potential or the low cytoplasmic Ca^2+^ levels consumes ATP (Berridge et al., 2003). ATP can be generated via glycolysis or through oxidative phosphorylation mediated by mitochondria (Bonora et al., 2012). It is well appreciated that mitochondrial and metabolic remodeling is a central feature of differentiation and reprograming events (Khacho and Slack, 2017). The mitochondrial oxidative metabolism is often suppressed in stem cells (Prigione et al., 2010; Cho et al., 2006). Stem cells, including intestinal stem cells, rely on glycolysis to generate ATP (Folmes et al., 2011; Schell et al., 2017). The mitochondria in stem cells remain functional (Folmes et al., 2011); however, stem cells possess multiple mechanisms to suppress mitochondrial activity (Schell et al., 2017; Zhang et al., 2016; Sanchez-Arago et al., 2013). Upon differentiation, mitochondrial activity increases (Folmes et al., 2011; Schell et al., 2017). On one hand, the increased mitochondrial activity fuels the high metabolic demand of the differentiated cells. On the other hand, the increased mitochondrial activity generates necessary signaling molecules such as reactive oxygen species and biosynthetic metabolites through the TCA cycle to promote the differentiation process (Khacho et al., 2016; Wellen et al., 2009). Our results revealed that immature EECs display low mitochondrial Ca^2+^ activity. When zebrafish develop and EECs start to be functional, mitochondrial activity increases. The increased mitochondrial activity may not only provide energy to fuel the EEC cellular process but also provide the signaling that is necessary for the EECs to mature and function. Interestingly, in addition to the change in mitochondrial activity, we also observed changes in intracellular mitochondrial distribution during development. Specifically, our results revealed that mature EECs display a hotspot mitochondrial distribution pattern, with high mitochondria contents near the base membrane, likely to match the ATP demand of the vesicle secretion process in the basal lateral membrane.

In addition to providing energy, mitochondria also function as an important Ca^2+^ buffer. In response to extracellular stimulation, cytoplasmic Ca^2+^ levels increase. This increase in cytoplasmic Ca^2+^ is quickly dissipated into intracellular organelles, such as the ER or mitochondria. In most cells, mitochondrial Ca^2+^ uptake is mediated by the mitochondrial calcium uniporter (MCU), a Ca^2+^ transporter protein in the mitochondrial inner membrane. The electrochemical potential across the mitochondrial inner membrane, generated by the respiration chain reaction, is the major driving force that enables Ca^2+^ influx into the mitochondrial matrix via MCU. Cytoplasmic and mitochondrial Ca^2+^ coupling have not been studied in EECs. Our studies revealed that, in response to nutrient stimulation, a subset of EECs increases cytoplasmic Ca^2+^ activity. In commensal microbiota colonized zebrafish, following the increase of cytoplasmic Ca^2+^ levels, the mitochondrial Ca^2+^ levels increase in activated EECs. Though the cytoplasmic Ca^2+^ quickly returned to the basal level, EEC mitochondrial Ca^2+^ was continuously maintained at a high level. Basal mitochondrial respiratory function might be the key to mediating Ca^2+^ flux into the mitochondrial matrix. The increase in mitochondrial Ca^2+^ will increase mitochondrial respiration to sustain the ATP production that is required for the EEC vesicle secretion in response to the nutrient stimulants (Devine and Kittler, 2018). Our results show that mitochondria are concentrated near the basal membrane, where vesicle secretion occurs. Nutrient-induced mitochondrial activation is also most prominent in the mitochondria near the basal membrane. This evidence supports the hypothesis that mitochondrial activation assists with vesicle secretion in mature EECs.

### EECs change morphology during development

In addition to the change in EEC mitochondrial activity, another hallmark of EEC maturation revealed by our study is the change in EEC morphology. Our study illustrated for the first time that immature EECs possess dynamic and active actin filaments in the basal membrane. However, the actin filaments disappeared in mature EECs. Instead, some mature EECs formed an elongated basal lateral membrane process, a structure that resembles the ‘neuropod’ reported in previous mammalian studies (Bohorquez et al., 2015). Previous studies demonstrated that the neuropod structure enriches the neurofilaments and mitochondria (Bohorquez et al., 2014). EECs use neuropods to form synaptic connections with the underlying nerve terminals, including the vagal sensory nerve (Bohorquez et al., 2015; Kaelberer et al., 2018). What regulates the EEC neuropod formation and guides the EEC-neuronal synaptic connection remains unknown. In developing neurons, neurites form actin-supported extensions known as growth cones which seek synaptic targets (Tamariz and Varela-Echavarria, 2015). Formation of the pre- and post-synaptic structures disables the filipodium-enriched actin structure at the leading age (Dent et al., 2011). Can the EECs form a growth cone-like structure to find their targets and form synaptic connections with the neurons? Our results revealed that the immature EECs form thin actin-based elements in the basal lateral membrane, a structure that is similar to the filopodia projections found in the developing neuron axon growth cone. In the zebrafish that are colonized with commensal microbiota, these thin actin filaments in some of the EECs are replaced by the ‘neuropod-like’ structure when EECs mature. This morphology evidence supported the hypothesis that the immature EECs may use active actin filaments to find the synaptic targets and form the synaptic connections with the underlying neurons. Establishing the EEC-neuronal connection will facilitate the EECs to transmit the ingested nutrients to the nervous system.

Previous studies have suggested that metabolism and mitochondria are key drivers of cell fate transitions in many systems, such as the developing brain. In the nervous system, the temporal pattern of mitochondria and metabolic development sets the tempo of neuronal maturation (Iwata et al., 2023). Stimulating mitochondrial metabolism and oxidative phosphorylation promotes neuronal maturation (Iwata et al., 2023; Rangaraju et al., 2019). It is, therefore, possible that the increased mitochondrial oxidation is the driver in promoting EEC maturation, which includes the remodeling of the actin filaments. Future studies applying genetic and pharmaceutical manipulation of the mitochondrial metabolism in EECs can be carried out to investigate further the relationship between EEC mitochondrial metabolism and actin remodeling.

### How do gut microbiota regulate EEC maturation and mitochondrial function?

A major finding revealed by our study is that commensal microbiota colonization is crucial in supporting EEC maturation and promoting EEC mitochondrial function. Our results established that microbiota colonization during early development might be essential in establishing the appropriate nutrient-sensing function for the organism via the promotion of EEC maturation. Similar to mammals, the proximal intestinal EECs in zebrafish are crucial for nutrient sensing. Our results revealed that the EECs remain in an immature state and exhibit low mitochondrial activity when commensal microbiota are absent. Disrupting the commensal microbiota colonization or inhibiting the formation of the healthy postnatal microbiome may produce devasting effects on gut nutrient perception and metabolic regulation. The formation of the postnatal gut microbial community is influenced by many factors (maternal microbiome, delivery method, milk-feeding versus formula feeding). Previous research has shown that disrupting the infant microbiome through antibiotic exposure results in many side effects, including obesity and weight gain later in life (Aversa et al., 2021). EECs are crucial in sensing ingested nutrients and maintaining homeostasis (Furness et al., 2013). Our study suggests that disrupting the commensal microbial community early in life will change EEC function and maturity, which may change how the body responds to ingested nutrients and affect energy homeostatic control.

The mitochondrial energetic adaptations encompass a conserved process that maintains fitness of cells and organisms in the changing environment (Bennett et al., 2022). Our studies suggest that, in response to the commensal microbiota colonization, the EECs increase mitochondrial respiration and enhance the mitochondrial Ca^2+^ activity. Our transcriptomic data revealed that microbial-induced EEC energy and mitochondrial adaptation are involved with increased mitochondrial cristae formation and increased mitochondrial respiratory chain assembly via enhancing mitochondrial protein import and facilitating protein translation in the mitochondrial matrix (Fig. 2I). EEC mitochondrial energetic adaptation in response to commensal microbiota colonization may contribute to the systemic host adaptation to microbial colonization that is to compete for the limited nutrients, enhance nutrient utilization efficiency and promote nutrient storage. The microbial and molecular mechanisms by which microbial signals regulate mitochondrial activity and intercede with the nutritional metabolism pathway within the EECs are intriguing questions that require future investigation.

## MATERIALS AND METHODS

### Zebrafish strains and husbandry

All zebrafish experiments conformed to the US Public Health Service Policy on Humane Care and Use of Laboratory Animals, using protocol number 2021A00000091 approved by the Institutional Animal Care and Use Committee of the Ohio State University. Conventionally-reared adult zebrafish were reared and maintained on a recirculating aquaculture system using established methods (Melancon et al., 2017). For experiments involving conventionally-raised zebrafish larvae, adults were bred naturally in system water and fertilized eggs were transferred to 100 mm petri dishes containing ∼25 ml of egg water at ∼6 hpf. The resulting larvae were raised under a 14 h light/10 h dark cycle in an air incubator at 28°C at a density of 2 larvae/ml water. All the experiments performed in this study ended at 7 dpf unless specifically indicated. The zebrafish lines used in this study are listed in Table S2. All lines were maintained on an EKW background.

### Gnotobiotic zebrafish husbandry

For experiments involving gnotobiotic zebrafish, we used our established methods to generate GF zebrafish using natural breeding (Ye et al., 2019) with the following exception: Gnotobiotic Zebrafish Medium (GZM) with antibiotics (AB-GZM) was supplemented with 50 μg/ml gentamycin (Sigma-Aldrich, G1264). GF zebrafish eggs were maintained in cell culture flasks with GZM at a density of 1 larvae/ml. From 3 dpf to 7 dpf, 60% daily media change and 250 µl of 1% newborn fish food (Ultra Fresh) feeding were performed as previously described (Melancon et al., 2017). Nutrient composition for newborn fish food (provided by the manufacturer): crude protein 42%, crude fat 2.6%, crude fiber 4%, moisture 3%, phosphorus 1%, calcium 3%. For each derivation experiment we combined eggs from three different clutches and, for each group, the derived GF zebrafish eggs were divided into three culture flasks. The samples were collected from the free flasks to prevent flask effects.

To generate conventionalized zebrafish, 15 ml filtered system water (5 μm filter, SLSV025LS, Millipore, final concentration of system water ∼30%) was inoculated to flasks containing GF zebrafish in GZM at 3 dpf, when the zebrafish normally hatch from their protective chorions. The same feeding and media change protocol was followed as for GF zebrafish. Microbial colonization density was determined via Colony Forming Unit (CFU) analysis. To analyze the effect of high fat feeding on intestinal bacteria colonization, digestive tracts were dissected and pooled (5 guts/pool) into 1 ml sterile phosphate buffered saline (PBS) and mechanically disassociated using a Tissue-Tearor (BioSpec Products, 985370). Then 100 µl of serially diluted solution was spotted on a Tryptic soy agar (TSA) plate and cultured overnight at 30°C under aerobic conditions.

### Zebrafish EEC RNA-seq analysis

The zebrafish EEC RNA-seq data was generated in our previous study (Ye et al., 2021). This dataset has been deposited in GEO under accession number GSE151711. CV and GF *TgBAC(cldn15la:EGFP); Tg(neurod1:TagRFP)* ZM000 fed zebrafish larvae were derived and reared using the published protocol (Melancon et al., 2017) for FACS to isolate zebrafish EECs and other IECs. The protocol for FACS was adopted from a previous publication (Ye et al., 2021). Replicate pools of 50-100 double transgenic *TgBAC(cldn15la:EGFP); Tg(neurod1:TagRPF)* zebrafish larvae were euthanized with Tricaine and washed with deyolking buffer (55 mM NaCl, 1.8 mM KCl and 1.25 mM NaHCO_3_) before they were transferred to dissociation buffer [HBSS supplemented with 5% heat-inactivated fetal bovine serum (HI-FBS, Sigma-Aldrich, F2442) and 10 mM HEPES (Gibco, 15630-080)]. Larvae were dissociated using a combination of enzymatic disruption using Liberase (Roche, 05 401 119 001, 5 μg/ml final), DNaseI (Sigma-Aldrich, D4513, 2 μg/ml final), Hyaluronidase (Sigma-Aldrich, H3506, 6 U/ml final) and Collagenase XI (Sigma-Aldrich, C7657, 12.5 U/ml final) and mechanical disruption using a gentleMACS dissociator (Miltenyi Biotec, 130-093-235). We then added 400 μl of ice-cold 120 mM EDTA (in 1× PBS) to each sample at the end of the dissociation process to stop the enzymatic digestion. Following addition of 10 ml Buffer 2 (HBSS supplemented with 5% HI-FBS, 10 mM HEPES and 2 mM EDTA), samples were filtered through 30 μm cell strainers (Miltenyi Biotec, 130-098-458). Samples were then centrifuged at 1800 ***g*** for 15 min at room temperature. The supernatant was decanted and cell pellets were resuspended in 500 μl Buffer 2. FACS was performed using a MoFlo XDP cell sorter (Beckman Coulter) at the Duke Cancer Institute Flow Cytometry Shared Resource. Single-color control samples were used for compensation and gating. Viable EECs or IECs were identified as 7-AAD negative.

Samples from three independent experimental replicates were used: 250-580 EECs (*n*=3 for each CV and GF group) and 100 IECs (*n*=3 for each CV and GF group) from each experiment were used for library generation and RNA-seq. Total RNA was extracted from cell pellets using the Argencourt RNAdvance Cell V2 kit (Beckman) following the manufacturer's instructions. RNA amplification was performed before library preparation. The Clontech SMART-Seq v4 Ultra Low Input RNA Kit (Takara) was used to generate full-length cDNA. mRNA transcripts were converted into cDNA using the Clontech oligo(dT)-priming method. Full length cDNA was then converted into an Illumina sequencing library using the Kapa Hyper Prep kit (Roche). In brief, cDNA was sheared using a Covaris instrument to produce fragments of ∼300 bp in length. Illumina sequencing adapters were then ligated to both ends of the 300 bp fragments before final library amplification. Each library was uniquely indexed allowing for multiple samples to be pooled and sequenced on two lanes of an Illumina HiSeq 4000 flow cell. Each HiSeq 4000 lane could generate >330 M 50 bp single end reads per lane. This pooling strategy generated enough sequencing depth (∼55 M reads per sample) for estimating differential expression. Sample preparation and sequencing was performed at the GCB Sequencing and Genomic Technologies Shared Resource.

Zebrafish RNA-seq reads were mapped to the danRer10 genome using HISAT2 (Galaxy Version 2.0.5.1) using default settings. Normalized counts and pairwise differentiation analysis were carried out via DESeq2. The significance threshold of *P*<0.05 was used for comparison.

### Immunofluorescence staining

Whole mount immunofluorescence staining was performed as previously described (Ye et al., 2019). In brief, ice cold 2.5% formalin was used to fix zebrafish larvae overnight at 4°C. The samples were then washed with PT solution (PBS+0.75%Triton X-100). The skin and remaining yolk were removed using forceps under a dissecting microscope. The deyolked samples were then permeabilized with methanol for more than 2 h at −20°C. Samples were then blocked with 4% bovine serum albumin at room temperature for more than 1 h. The primary antibody was diluted in PT solution and incubated at 4°C for more than 24 h. Following primary antibody incubation, the samples were washed with PT solution and incubated overnight with secondary antibody with Hoechst 33342 for DNA staining. Imaging was performed using a Nikon AXR confocal microscope using the 20× or 40× water immersion lens. The primary antibodies are listed in Table S2. The secondary antibodies used in this study were from Alexa Fluor Invitrogen (Life Technologies, A32934, A11036, A32731, A32723) and were used at a dilution of 1:250.

### Live imaging

The zebrafish larvae were anesthetized with Tricaine methanesulfonate (MS222) and were mounted in the 35 mm confocal dish using 1% low-melting-Agar. All the *in vivo* imaging was performed using the Nikon AXR confocal microscope. When imaging the EEC cellular and mitochondrial Ca^2+^ activity using the *Tg(neurod1:Gcamp6f); Tg(neurod1:mitoRGECO)* zebrafish, the zebrafish were not anesthetized due to the effects of Tricaine in activating EECs. In the developmental tracing experiments, after imaging, the zebrafish were dug out of the Agar, placed in a 6-well plate, and returned to the incubator until the next imaging time point. In the experiments when the temporal EEC activity was traced, the images were collected using the resonate scanner. It takes less than 10 s to collect the whole intestinal *z*-stack. The interval of time frames is 10 s. In the experiments when the nutrient stimulants were applied, a small window was cut in front of the zebrafish, which allowed the zebrafish mouth to be exposed. First, the zebrafish intestine was imaged before the stimulants were applied to assess the basal line EEC activity. After collecting the baseline EEC activity, the image acquisition was pulsed, and nutrient stimulants were added. The egg water in the confocal dish was removed and 1 ml nutrient stimulate solution was delivered into the window in front of the zebrafish. After the nutrient stimulation was applied, the image acquisition process resumed. The time-lapse images were collected to assess the nutrient-induced EEC activation.

For live imaging, the confocal setting was optimized for each experiment, and the same confocal setting was used for different groups in an individual experiment. Therefore, for live imaging data, the value from one experiment cannot be compared with another experiment.

### Image analysis

For the image analysis to assess the EEC Ca^2+^ activity, the images were first aligned using the Nikon NLS element software 3D segmentation and 3D tracking function. The EECs are selected and segmented by the Gcamp fluorescence based on the threshold intensity. The threshold was applied to identify more than 95% of EECs, and the threshold was consistent across one experiment. Following threshold identification, image smoothing was applied with the parameter 0.85 µm, objects less than 1 µm were removed, and objects with a distance of more than 0.22 µm were separated. Following segmentation, for each object unit the mean Gcamp6f fluorescence intensity, mean mitoRGECO fluorescence intensity and object volume were measured. The objects with a volume outside 100 µm^3^-1000 µm^3^ were filtered out. Following segmentation, the individual object units in different time frames were traced and tracked via the NLS element 3D-object tracing software. Due to the issues of gut motility, not every EEC in the zebrafish can be successfully traced throughout the time course. The mean fluorescence intensity of the individual EEC in each time frame was calculated. Cluster 3.0 software was used to perform the clustering analysis of the EECs that exhibit different temporal Ca^2+^ dynamics.

To quantify the intracellular EEC mitochondrial distribution, a *z*-projection of an EEC was performed. 3D projections of the EECs were made. The EECs were arranged with the base on the left and apex on the right. A rectangle was drawn to outline the EEC, and the mitoEOS plot profile was analyzed in the EECs.

### Statistical analysis

For each experiment, wild-type or indicated transgenic zebrafish embryos were randomly allocated to test groups before treatment. For the experiments that are involved with fixed samples, ≥8 biological replicates were used for each experimental group. For some experiments involved in EEC Ca^2+^ imaging, <8 zebrafish were analyzed due to the technique limitation involved with live imaging. For each gnotobiotic experiment, at least two independent derivation experiments were performed, and the same conclusion was reached. Individual data points, mean and standard deviation are plotted in each figure. The raw data points in each figure are represented as solid dots. The data was analyzed using GraphPad Prism 7 software. For experiments comparing two differentially treated populations (except for Fig. 7O,P), unpaired, two-tailed Student's *t*-test with equal variance assumptions was used. For data presented in Fig. 7O,P, paired, two-tailed Student's *t*-test was performed. For experiments measuring a single variable with multiple treatment groups, a single factor ANOVA with post hoc means testing (Tukey) was used. Statistical evaluation for each figure was marked: *P<0.05, **P<0.01, ***P<0.001, ****P<0.0001 or n.s. (no significant difference, *P*>0.05).

## Supplementary Material



10.1242/develop.202544_sup1 Supplementary information

Table S1. The change of transcriptomics in GF and CV EECs.

Table S2. The zebrafish lines and primary antibodies used in this manuscript.
