# Supplementary material for: Gut microbiota regulate maturation and mitochondrial function of the nutrient-sensing enteroendocrine cell
Source: Development. 2024 Apr 30;151(8):dev202544. doi: 10.1242/dev.202544 (PMC11112165; doi:10.1242/dev.202544)
Supplement: Supplementary information [file develop-151-202544-s1.pdf]

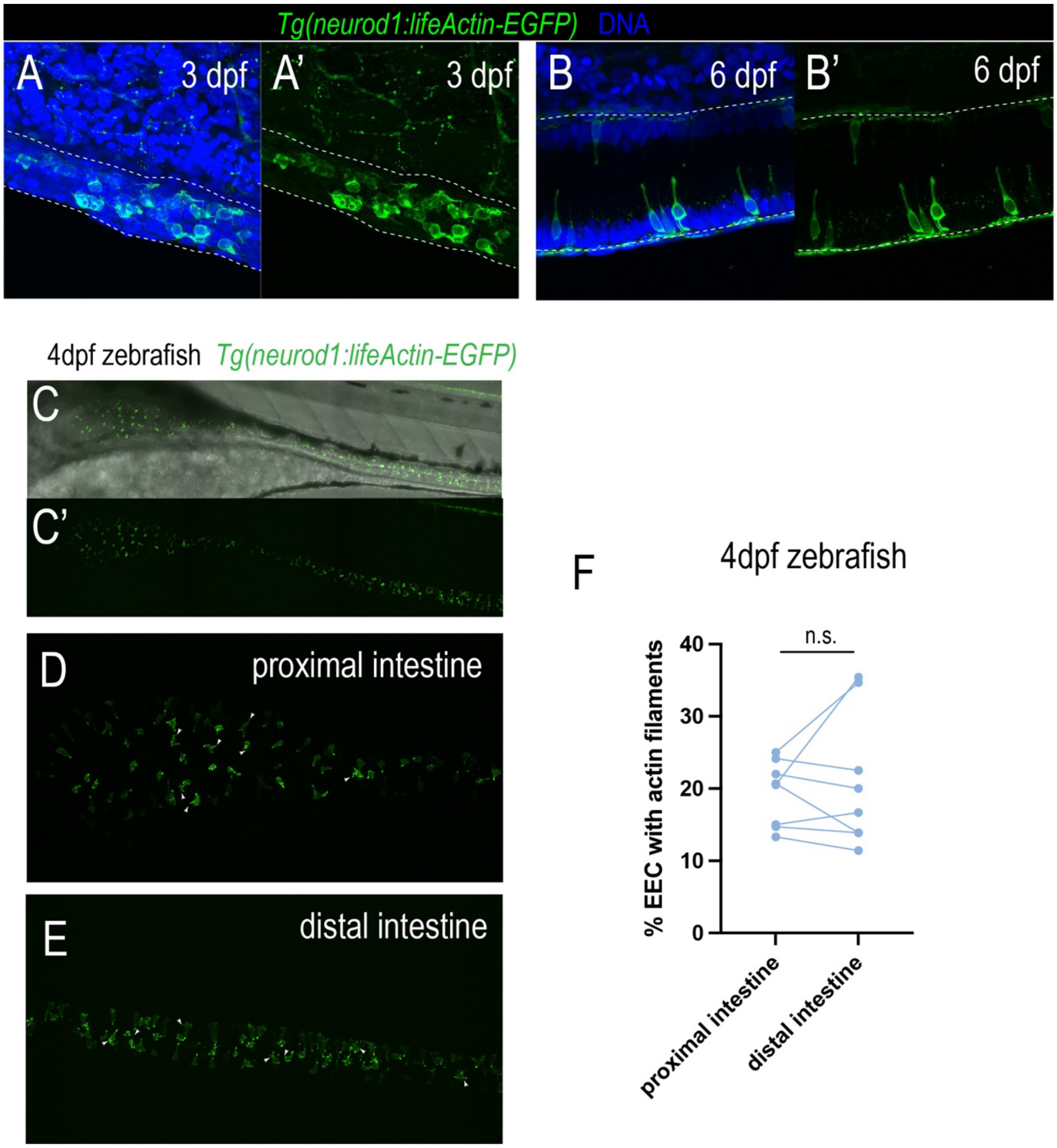

Fig. S1. EEC actin filaments in proximal and distal zebrafish intestine. (A-B') Confocal projection of 3dpf and 6dpf zebrafish distal intestine. Noted the EECs in 3dpf but not 6dpf distal intestine display active actin filaments. (C-C') Confocal projection of a 4dpf zebrafish intestine. The EECs' actin filaments were labeled by lifeActin-EGFP. (D-C) Zoom in view showed the proximal and distal intestine from the same zebrafish. White arrows indicate the EECs that display active actin filaments. (F) Quantification of the percentage of EECs that display active actin filaments in the same zebrafish proximal and distal intestine. Each dot represents an individual zebrafish. Paired, two-tailed Student's t-test were used in F for statistical analysis.

Development • Supplementary information

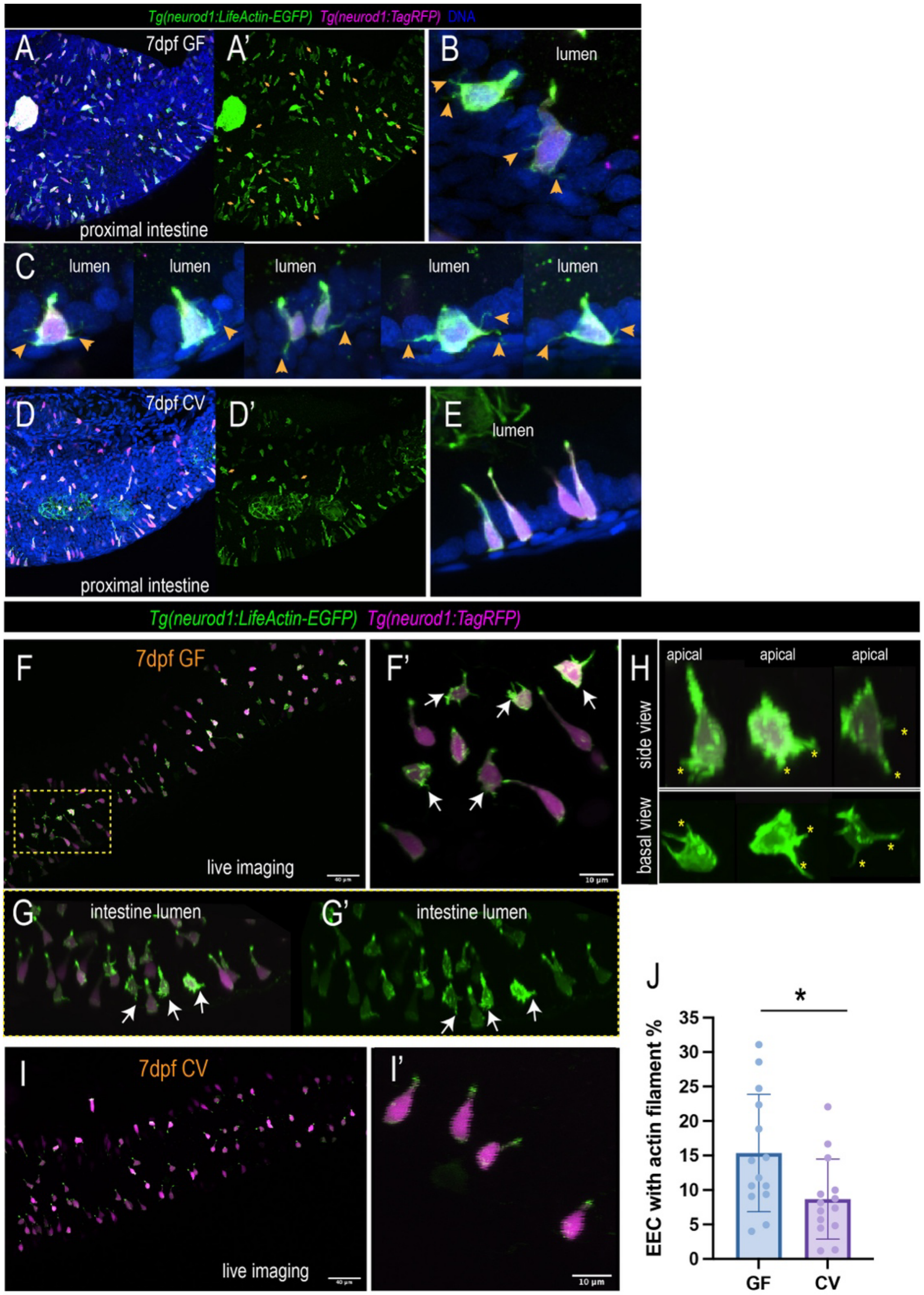

Fig. S2. Gut microbiota promotes EEC actin remodeling in 7dpf zebrafish. (A-A') Confocal projection of 7dpf *Tg(neurod1:lifeActin-EGFP); Tg(neurod1:RFP)* GF zebrafish proximal intestine. Yellow arrows indicate the EECs with actin filaments that are labeled by lifeActin-EGFP. (B-C) Representative EECs in 7dpf GF zebrafish proximal intestine. Yellow arrowheads indicate the actin filaments at the EEC base labeled by lifeActin-EGFP but not by RFP. (D-D') Confocal projection of 7dpf CV zebrafish proximal intestine. Yellow arrows indicate the EECs with actin filaments that are

Development • Supplementary information

labeled by lifeActin-EGFP. (E) Representative EECs in 7dpf CV zebrafish proximal intestine. (F) Live imaging of 7dpf *Tg(neurod1:lifeActin-EGFP); Tg(neurod1:RFP)* GF zebrafish. Confocal projections of the proximal intestine were presented. (F') Zoom in views of representative EECs in 7dpf GF. White arrows indicate the EECs with basal actin filaments. (G-G') 3D reorientation of the projection region circled in F and the intestinal lumen is now facing up. White arrows indicate 3 EECs with actin filaments at the base. (H) Zoom in views of the 3 EECs labeled by white arrows in G. Both side view (apical facing up) and basal view (apical facing inward) were presented for each EEC. Yellow stars indicate the actin filaments at the EEC base. (I) Live imaging of 7dpf *Tg(neurod1:lifeActin-EGFP); Tg(neurod1:RFP)* CV zebrafish. Confocal projections of the proximal intestine were presented. (I') Zoom in views of representative EECs in 7dpf CV zebrafish EECs. (J) Quantification of the percentage of EECs with actin filaments in 7dpf GF and CV zebrafish. Each dot represents an individual zebrafish. Unpaired, two-tailed Student's t-test were used in J for statistical analysis. * $P<0.05$.

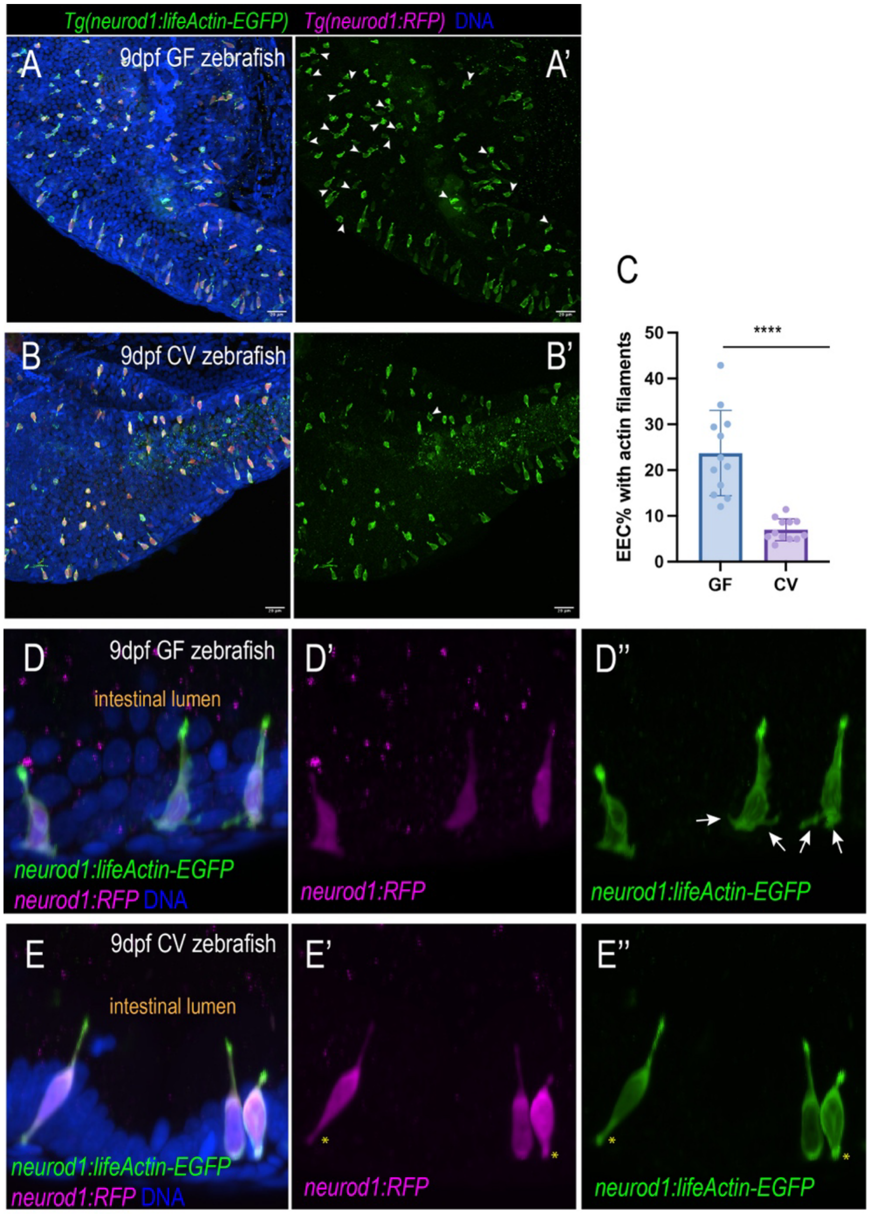

Fig. S3. Gut microbiota promotes EEC actin remodeling in 9dpf zebrafish. (A-B') Confocal projection of 9 dpf *Tg(neurod1:lifeActin-EGFP); Tg(neurod1:RFP)* GF and CV zebrafish proximal intestine. White arrows indicate the EECs with actin filaments that are labeled by lifeActin-EGFP. (C) Quantification of the percentage of EECs with actin filaments in 9 dpf GF and CV zebrafish proximal intestine. (D-E'') Representative EECs in 9dpf GF and CV zebrafish proximal intestine. White arrows indicate the actin filaments at the base of 9dpf GF zebrafish EECs that are labeled by lifeActin-EGFP but not by RFP. Yellow stars in E' and E'' indicate the "neuropod" like structure at the 9dpf CV zebrafish EECs that are labeled by both lifeActin-EGFP and RFP. Each dot in C represents an individual zebrafish. Samples were generated from one derivation experiment. Unpaired, two-tailed Student's t-test was used in J for statistical analysis. **** P<0.0001.

Development • Supplementary information

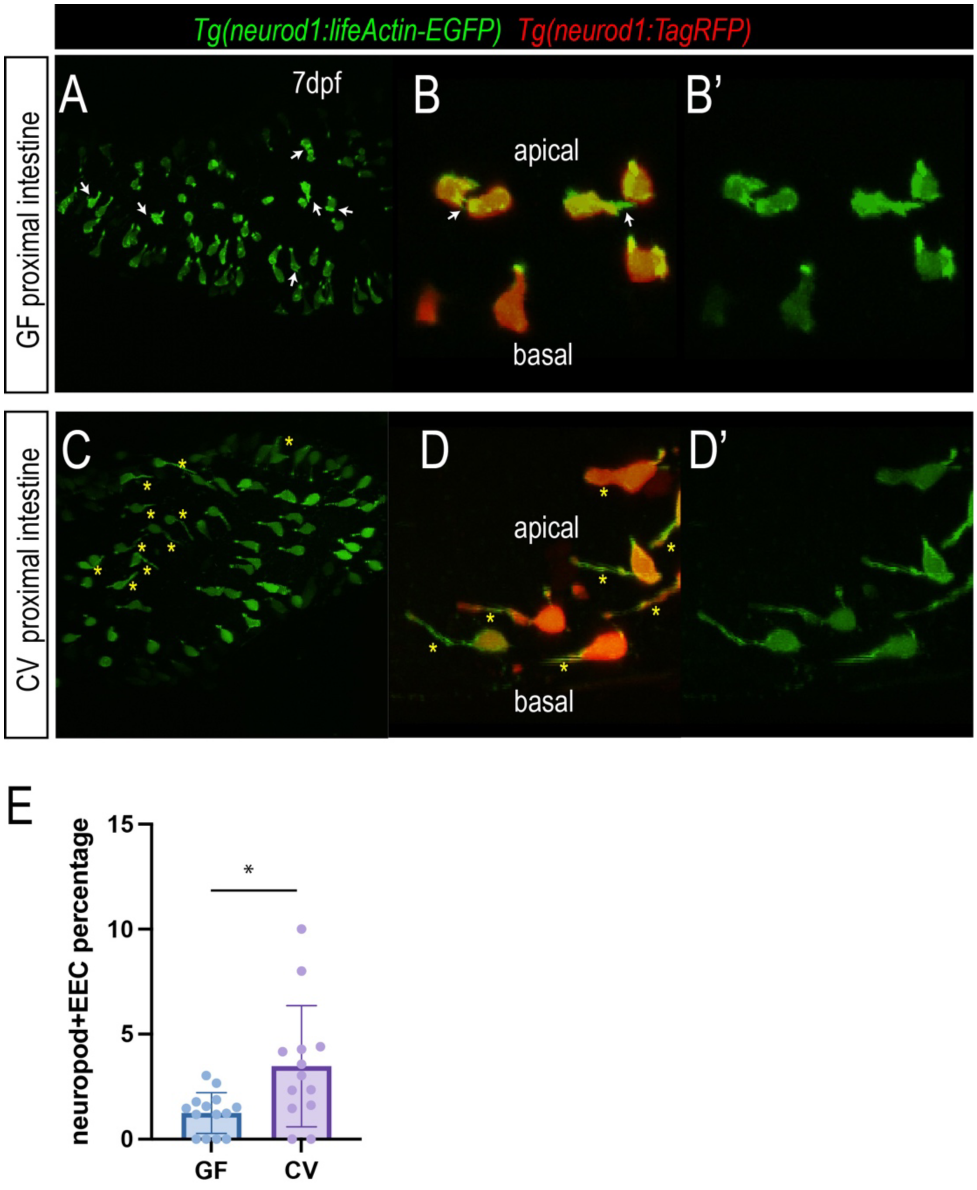

Fig. S4. Commensal microbiota colonization promotes the formation of "neuropod"-like structure in EECs. (A-D') Confocal projections of the 7dpf GF and CV *Tg(neurod1:lifeActin-EGFP) Tg(neurod1:TagRFP)* zebrafish proximal intestine. The white arrows in A and B indicate EECs with thin actin filaments at the 7dpf EECs' basal lateral membrane that are labeled by lifeActin-EGFP but not by RFP. The yellow stars in C and D indicate the "neuropod" like elongated basal lateral membrane protrusions in CV EECs that are labeled by both lifeActin-EGFP and RFP. (E) Quantification of the EEC percentage that has "neuropod" like structure in GF and CV conditions. Each dot represents an individual zebrafish. 14 GF zebrafish and 13 CV zebrafish from one derivation experiments were analyzed. Unpaired, two-tailed Student's t-test was used in E. *P<0.05.

Development • Supplementary information

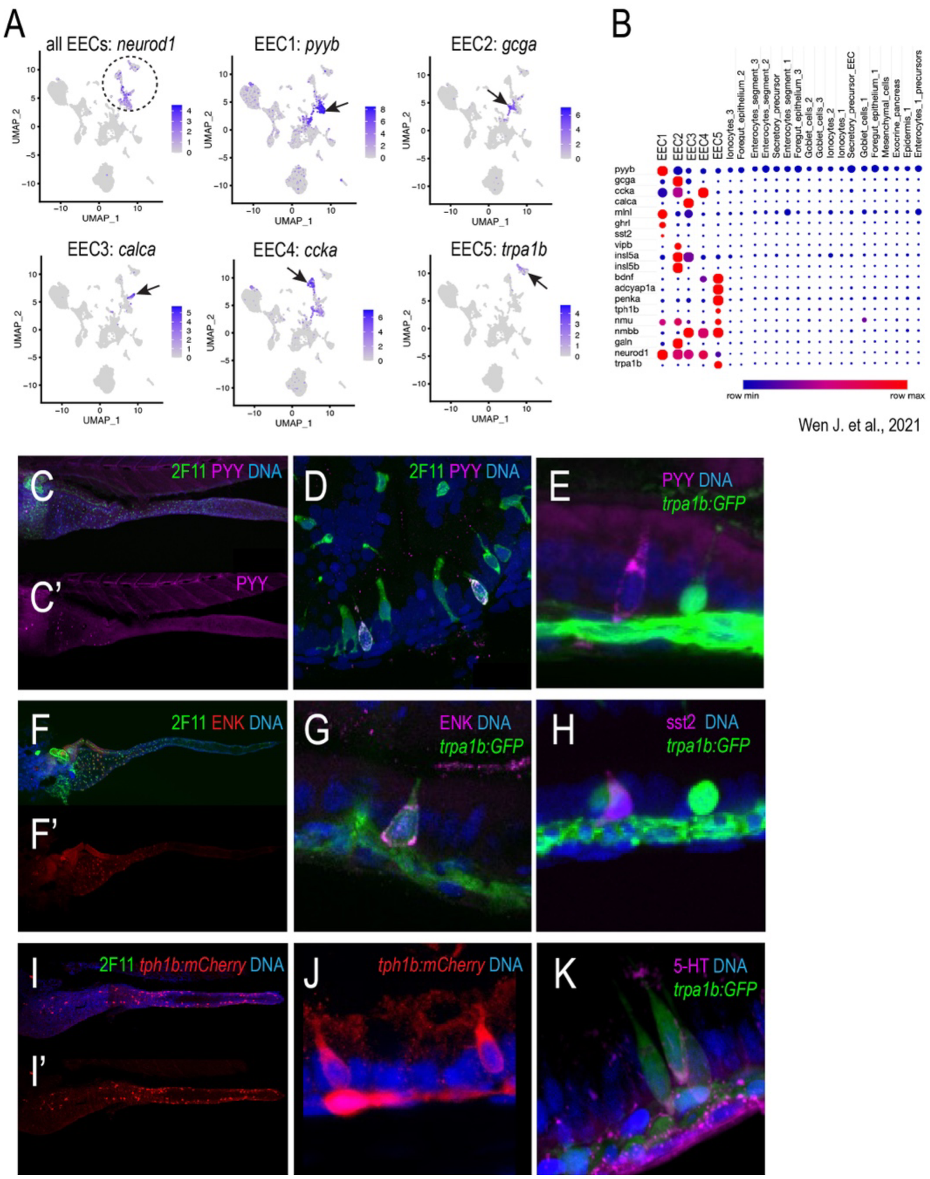

Fig. S5. The EEC subtypes in zebrafish larvae. (A) UMAP plots of the zebrafish intestine single-cell RNA sequencing showing the zebrafish EECs and the five EEC subtypes in zebrafish larvae. The zebrafish scRNA dataset is from Wen J. et al., 2021. (B) The hormone profiles in the five zebrafish EEC subtypes. (C-E) Immunofluorescence staining of the PYY +EEC subtype. Note that the PYY+EECs are distributed in the proximal zebrafish intestine (C-C'). It overlaps with the secretory cell marker 2F11 (D) but does not overlap with the marker for other EEC subtypes, such as trpa1b (E). (F-K) Immunofluorescence staining of the Trpa1+EEC subtype. The single-cell RNA seq data above demonstrate that the Trpa1+EECs (EEC5) express the peptide enkephalin (ENK) and the enzyme that synthesizes serotonin (tph1b). (F-G) Immunofluorescence staining of ENK confirms that only Trpa1+EECs express ENK (G). Interestingly, ENK is only expressed in the Trpa1+EECs in the proximal intestine (F-F'). (H) Trpa1+EECs do not express sst2, a marker for the EEC subtype 1. (I-J) Tph1b is expressed in the EECs. (K) Immunofluorescence staining showing part of the Trpa1+EECs express 5-HT.

Development • Supplementary information

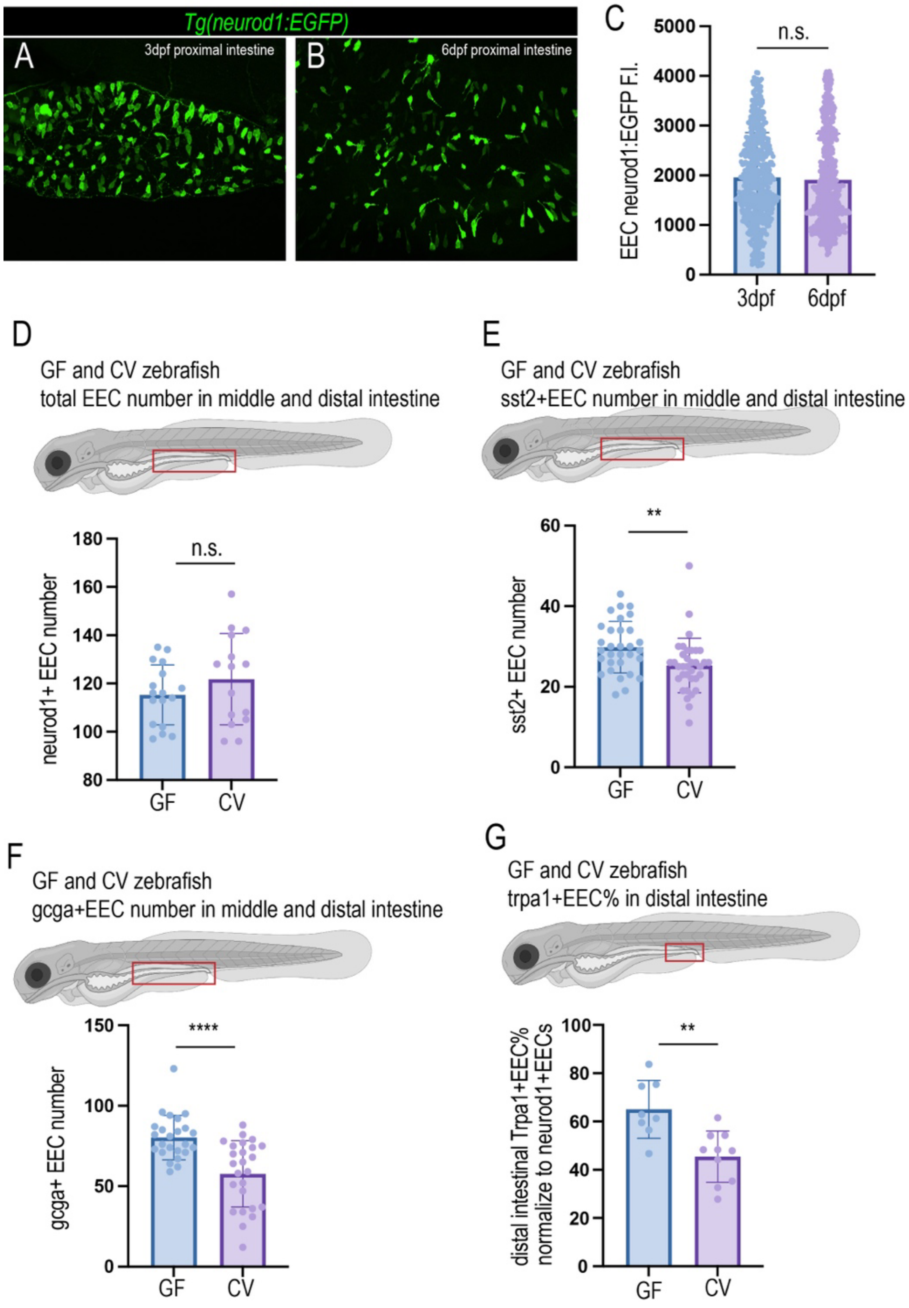

Fig. S6. Commensal gut microbiota colonization regulates EEC subtype specification. (A-B) Confocal projection of 3dpf and 6dpf zebrafish proximal intestine. EECs were labeled by *neurod1:EGFP*. (C) Quantification of EEC neurod1:EGFP fluorescence intensity in 3dpf and 6dpf zebrafish proximal intestinal EECs. Each dot represents an individual EEC. 546 EECs from 6 3dpf zebrafish and 583 EECs from 6 6dpf zebrafish were analyzed. (D-F) Quantification of the total EEC number, sst2+EEC number, and gcga+EEC number in the middle and distal intestine of 7dpf GF and CV zebrafish. The analyzed intestinal region was circled in the red box. *Tg(neurod1:EGFP)* was used to quantify the total EEC number. *Tg(sst2:RFP)* was used to quantify the total sst2+EEC number. *Tg(gcga:EGFP)* was used to quantify the total gcga+EEC number. (G) Quantification of the percentage of Trpa1+EECs in the distal intestine. Trpa1+EEC percentage is normalized to the total EEC number labeled by *neurod1:RFP*. Unpaired, two-tailed Student's t-test was used in C-G. ** $P<0.01$, **** $P<0.0001$.

Development • Supplementary information

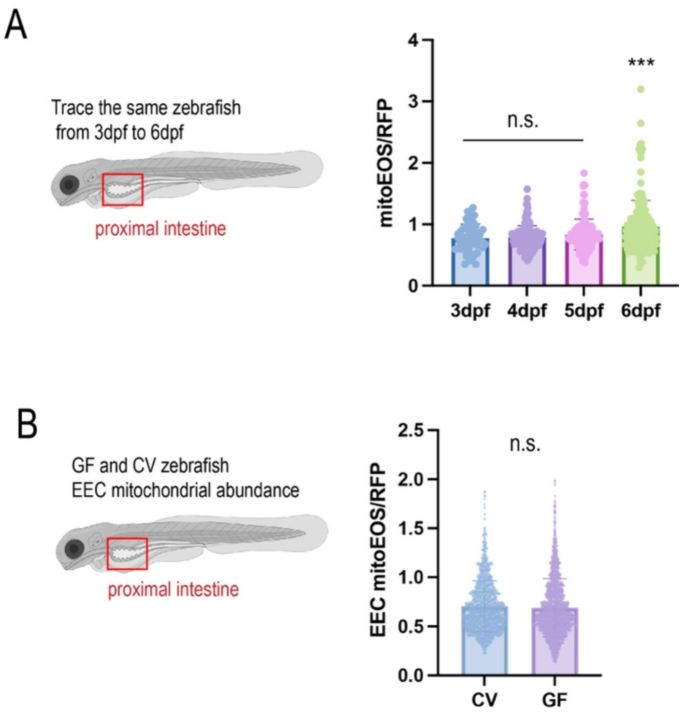

Fig. S7. Gut microbiota did not alter the proximal intestinal EEC mitochondria abundance. (A) Quantification of the intracellular mitochondria abundance in the 3dpf to 6dpf zebrafish proximal intestinal EECs. The mitochondrial abundance is represented by the *neurod1:mitoEOS* and *neurod1:RFP* fluorescence ratio in individual EECs. Each dot represents an EEC. The same zebrafish were traced from 3dpf to 6dpf, and 4 zebrafish were analyzed. (B) Quantification of the intracellular mitochondria abundance in the proximal intestines of the GF and CV zebrafish EECs. The mitochondrial abundance is represented by the *neurod1:mitoEOS* and *neurod1:RFP* fluorescence ratio in individual EECs. More than 7 zebrafish in GF and CV groups were quantified in B. One-Way ANOVA followed by Tukey post-test was used in A. Unpaired, two-tailed Student's t-test was used in A and B. *** P<0.001.

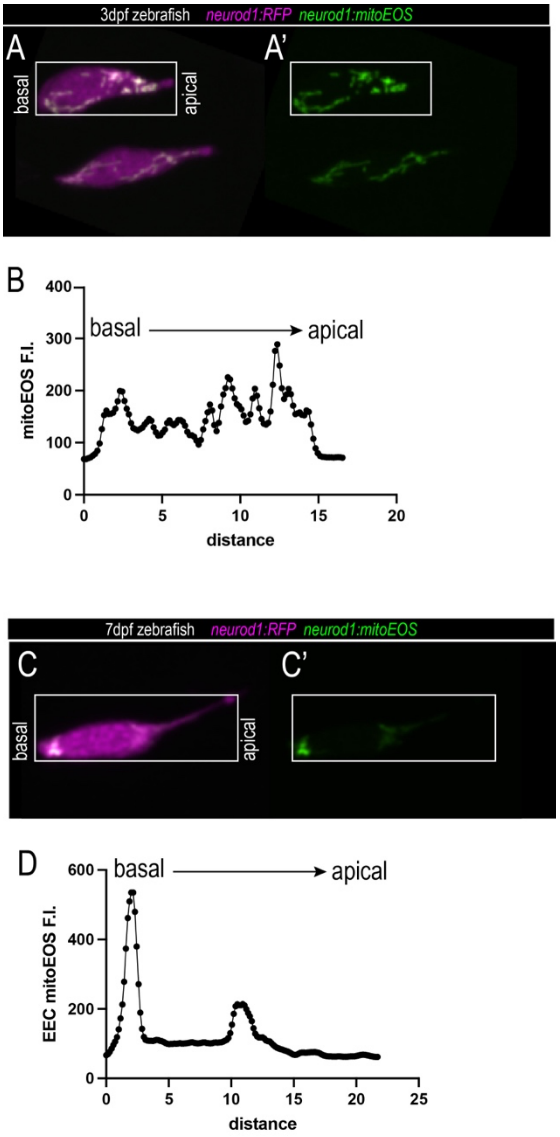

Fig. S8. Mature EECs alter intracellular mitochondrial distribution. (A-A') Two representative EECs in 3dpf zebrafish proximal intestine. The EECs were arranged with the base on the left and apical on the right. A rectangle was drawn to circle the EEC, and the mitoEOS plot profile was analyzed in this EEC. (B) MitoEOS plot profile for the EEC that was circled in A. (C-C') A representative EECs in 7dpf zebrafish proximal intestine. (D) MitoEOS plot profile for the EEC that was circled in C.

Development • Supplementary information

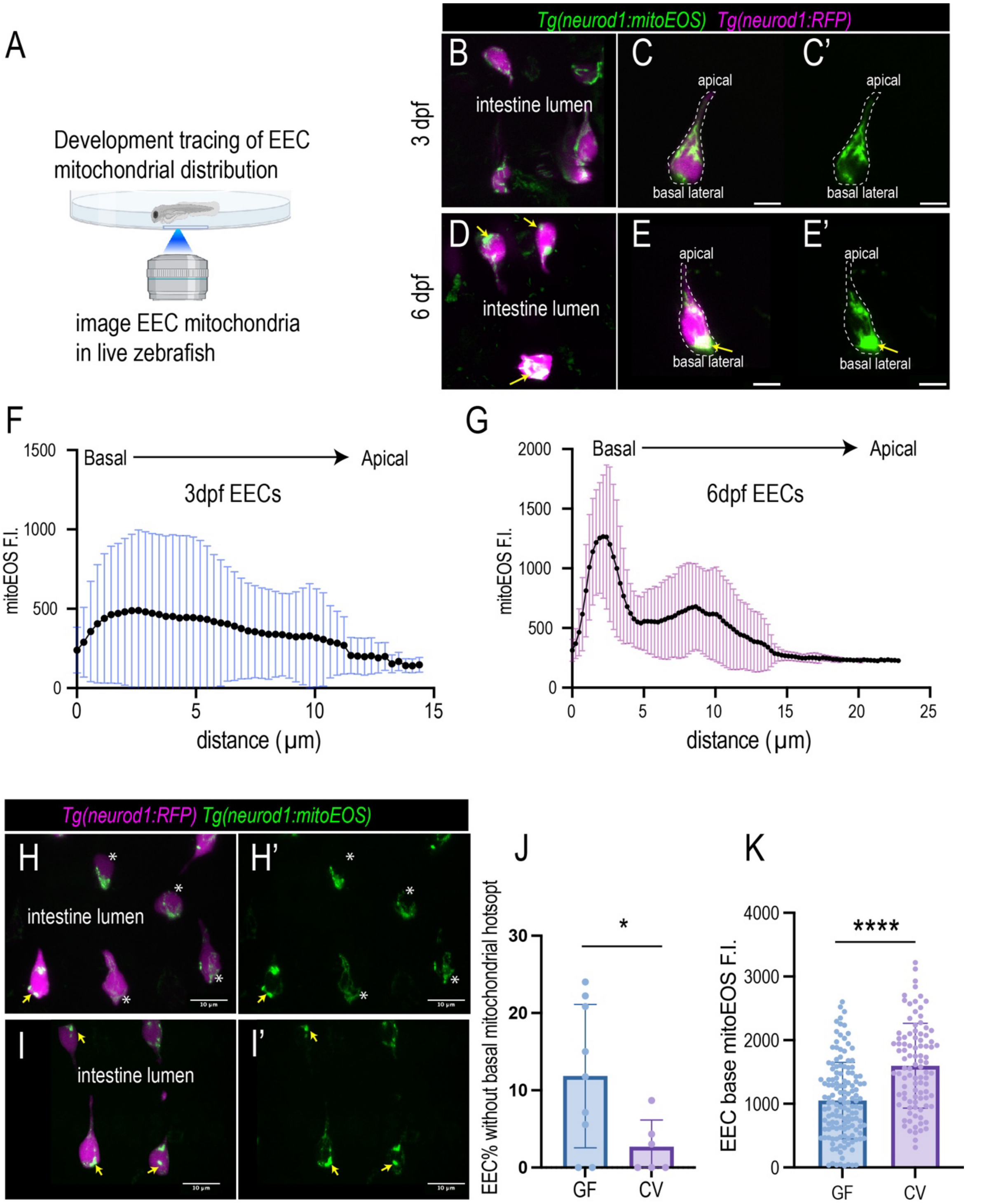

Fig. S9. Commensal microbiota colonization promotes mitochondria accumulation at the EEC base. (A-E') In vivo imaging to trace the EEC mitochondria in the same zebrafish from 3dpf to 6dpf. The mitochondria are labeled via the *Tg(neurod1:mitoEOS)*, and the EECs are labeled via the *Tg(neurod1:RFP)*. (B-C') Confocal projections of the typical EECs at 3dpf zebrafish proximal intestine. (D-E')

Development • Supplementary information

Confocal projections of the typical EECs at 6dpf zebrafish proximal intestine. Note that at 3dpf, the mitochondria are evenly distributed in the EEC cytoplasm, and the mitochondria contents at the basal lateral membrane are low. At 6dpf, the mitochondria distribution exhibits a hot spot pattern. In most EECs, a spot at the basal lateral membrane (yellow arrows in D-E') contains highly abundant mitochondria. (F-G) Quantification of EEC mitochondrial distribution profile from the same zebrafish at 3dpf and 6dpf. 25 representative EECs from 5 3dpf zebrafish and 25 representative EECs from 5 6dpf zebrafish were analyzed. (H-I') Confocal projections of the typical EECs in 7dpf GF and CV zebrafish proximal intestine. Images were captured in live zebrafish. Note that many EECs in GF zebrafish have low mitochondria contents at the basal lateral membrane (white stars in H and H'). Most EECs in CV zebrafish exhibit hot spot basal lateral membrane mitochondrial distribution patterns (yellow arrows in I and I'). (J) Quantification of the percentage of EECs without basal mitochondrial hotspots in 7dpf GF and CV zebrafish. Each dot represents an individual zebrafish. 170 EECs from 9 GF zebrafish and 194 EECs from 6 CV zebrafish from the same derivation experiment were analyzed. (K) Quantification of the mitochondrial fluorescence intensity at the basal membrane in 7dpf GF and CV zebrafish. Each dot represents an individual EEC. 141 EECs from 9 GF zebrafish and 96 EECs from 6 CV zebrafish were analyzed. Unpaired, two-tailed Student's t-test was used in J and K. * $P<0.05$, **** $P<0.0001$.

Development • Supplementary information

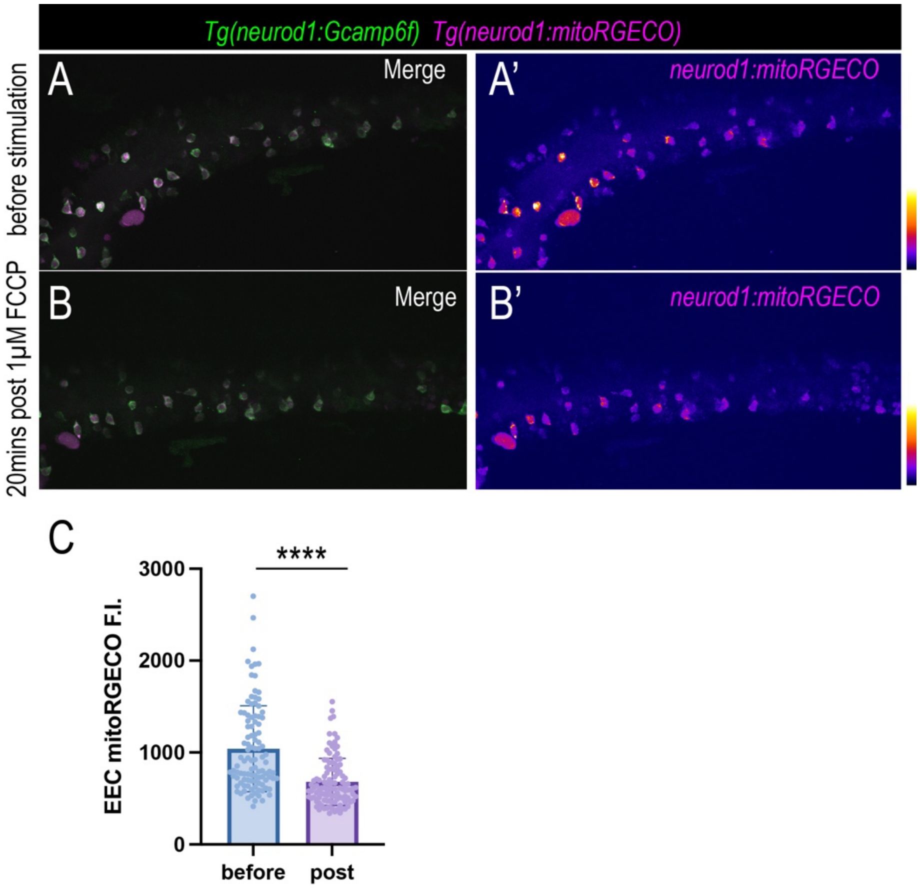

Fig. S10. FCCP decreases EECs' mitochondrial calcium. (A-B') Confocal projection of the same zebrafish proximal intestine before and 20 minutes post 1μM Carbonyl cyanide-p-trifluoromethoxyphenylhydrazone (FCCP) treatment. The same intestinal region at the same zebrafish was imaged. (C) Quantification of EEC mitoRGECO fluorescence intensity before and 20 minutes post FCCP treatment. Each dot represents one EEC. >100 EECs from 3 zebrafish were analyzed. Unpaired, two-tailed Student's t-test was used in C. **** P<0.0001.

Development • Supplementary information

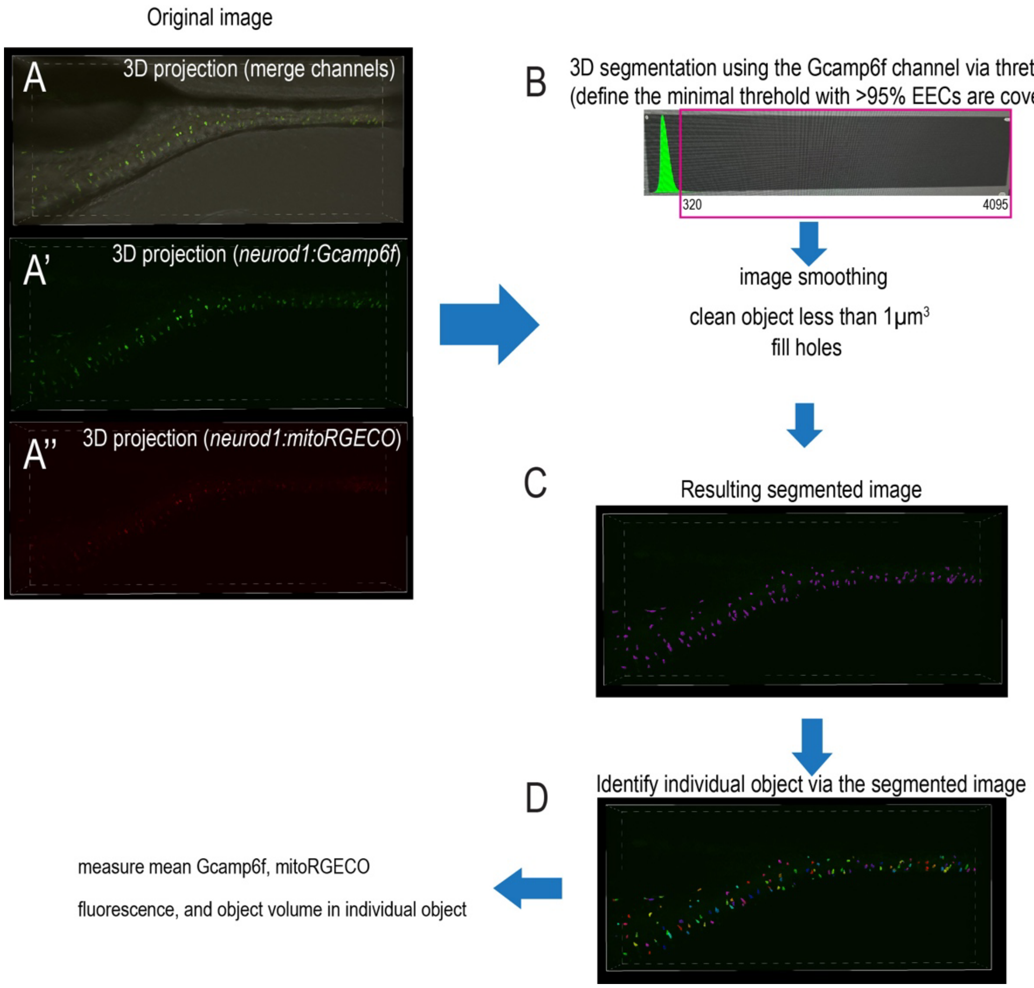

Fig. S11. EEC 3D segmentation. (A-A'') Projection of *Tg(neurod1:Gcamp6f); Tg(neurod1:mitoRGECO)* zebrafish intestine. (B) EECs were identified and segmented via the neurod1:Gcamp6f channel via the threshold. The threthold were applied to allow more than 95% of EECs included. Parameter for image smooth 0.86 µm. Object size less than 1 μm^3 were removed. Fillholes On. Separate object with distance more than 0.22 µm. (C) Resulting image with segmented EECs. (D) Color code shows the EEC objects identified from the segmented image in C. Following object identification, mean Gcamp6f fluorescence, mitoRGECO fluorescence, and object volume ware calculated for each EEC object.

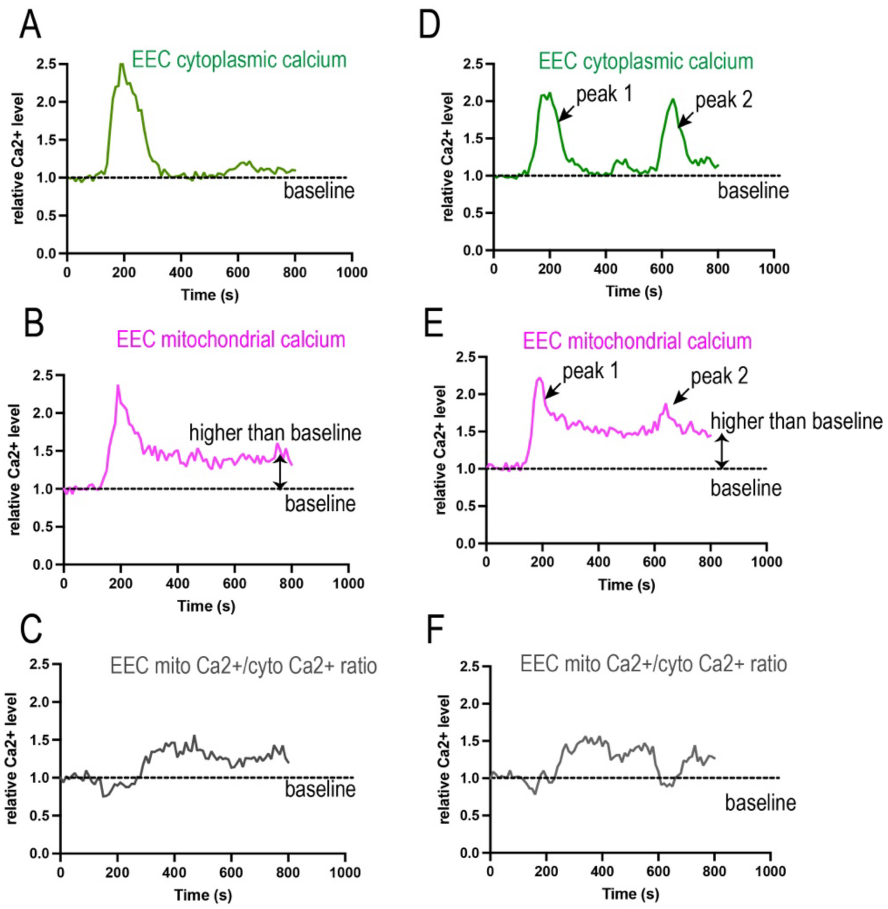

Fig. S12. Representative EEC cytoplasmic Ca^{2+} and mitochondrial Ca^{2+} upon linoleic acid stimulation. (A-C) One representative linoleic acid activated EEC temporal profiles for cytoplasmic Ca^{2+}, mitochondrial Ca^{2+}, and mitochondrial/ cytoplasmic Ca^{2+} ratio. Note that while the EEC's cytoplasmic Ca^{2+} returned to baseline after the peak, EECs' mitochondrial Ca2+ remained higher than baseline after the peak, resulting in an increased mito/cyto Ca^{2+} ratio. (D-F) A second representative linoleic acid activated EEC temporal profiles for cytoplasmic Ca^{2+}, mitochondrial Ca^{2+}, and mitochondrial/cytoplasmic Ca^{2+} ratio. Note that following linoleic acid stimulation, two Ca^{2+} peaks were detected in this EEC. While cytoplasmic Ca^{2+} activation is comparable for these two peaks, the mitochondria Ca^{2+} activation is much reduced for peak 2, resulting in a reduced mito/cyto Ca^{2+} ratio during the second Ca^{2+} peak.

Development • Supplementary information

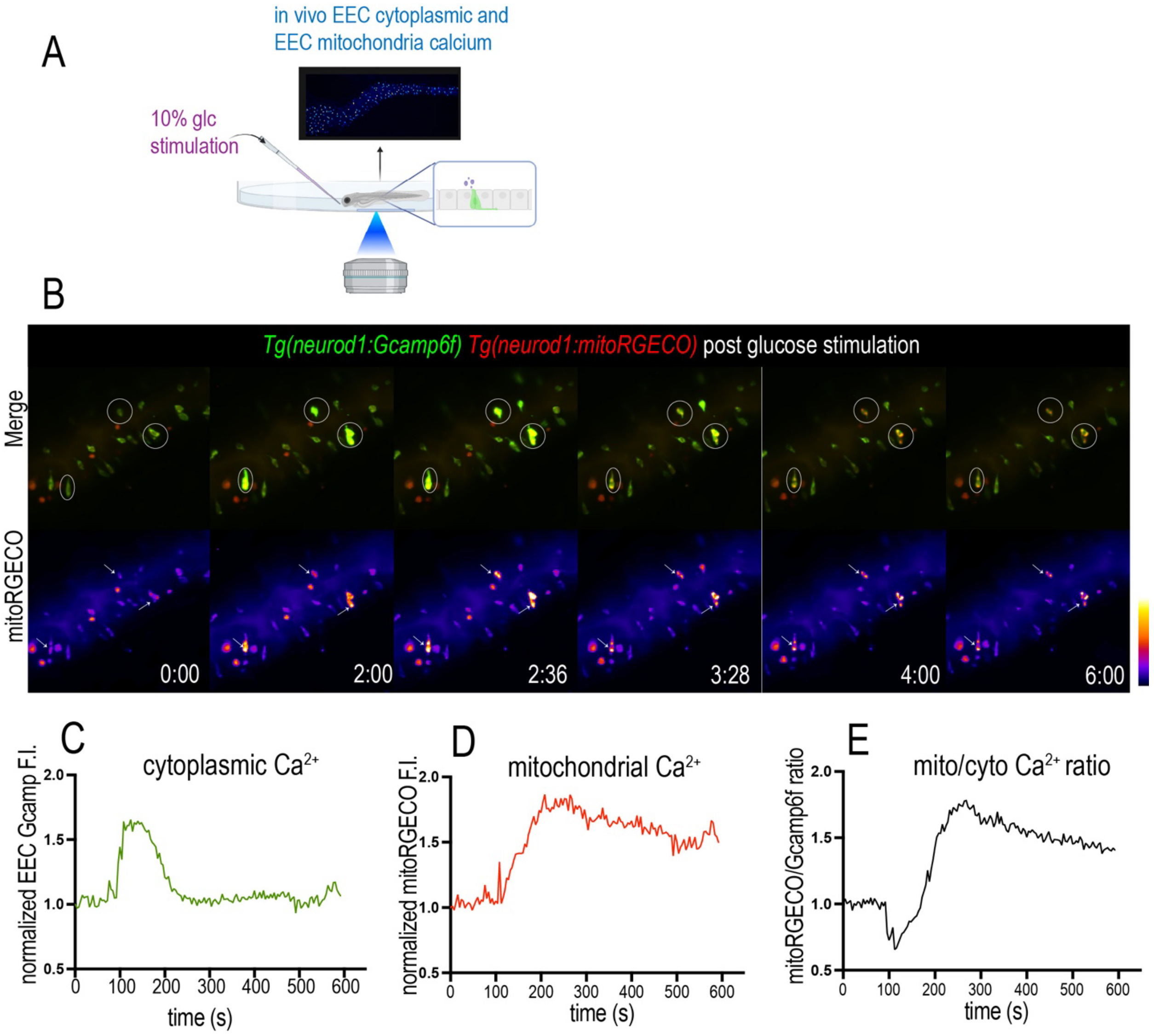

Fig. S13. Glucose stimulates EEC cytoplasmic and mitochondrial Ca^{2+}. (A) *Tg(neurod1:Gcamp6f); Tg(neurod1:mitoRGECO)* zebrafish were stimulated with 10% glucose solution and the zebrafish intestine was imaged. (B) Representative time-lapse images of the proximal intestine following glucose stimulation. Three glucose-activated EECs were circled, and mitochondrial activation was labeled by white arrows. (C-E) A represented glucose activated EEC's profile for cytoplasmic Ca^{2+}, mitochondrial Ca2+, and mito/cyto Ca2+ ratio. Noted that similar to linoleic acid stimulation, following glucose stimulation, EEC's cytoplasmic Ca^{2+} returns back to basal level after peak. However, EEC's mitochondrial Ca^{2+} remains higher than basal level after peak, resulting in an increased mito/cyto Ca^{2+} ratio.

Development • Supplementary information

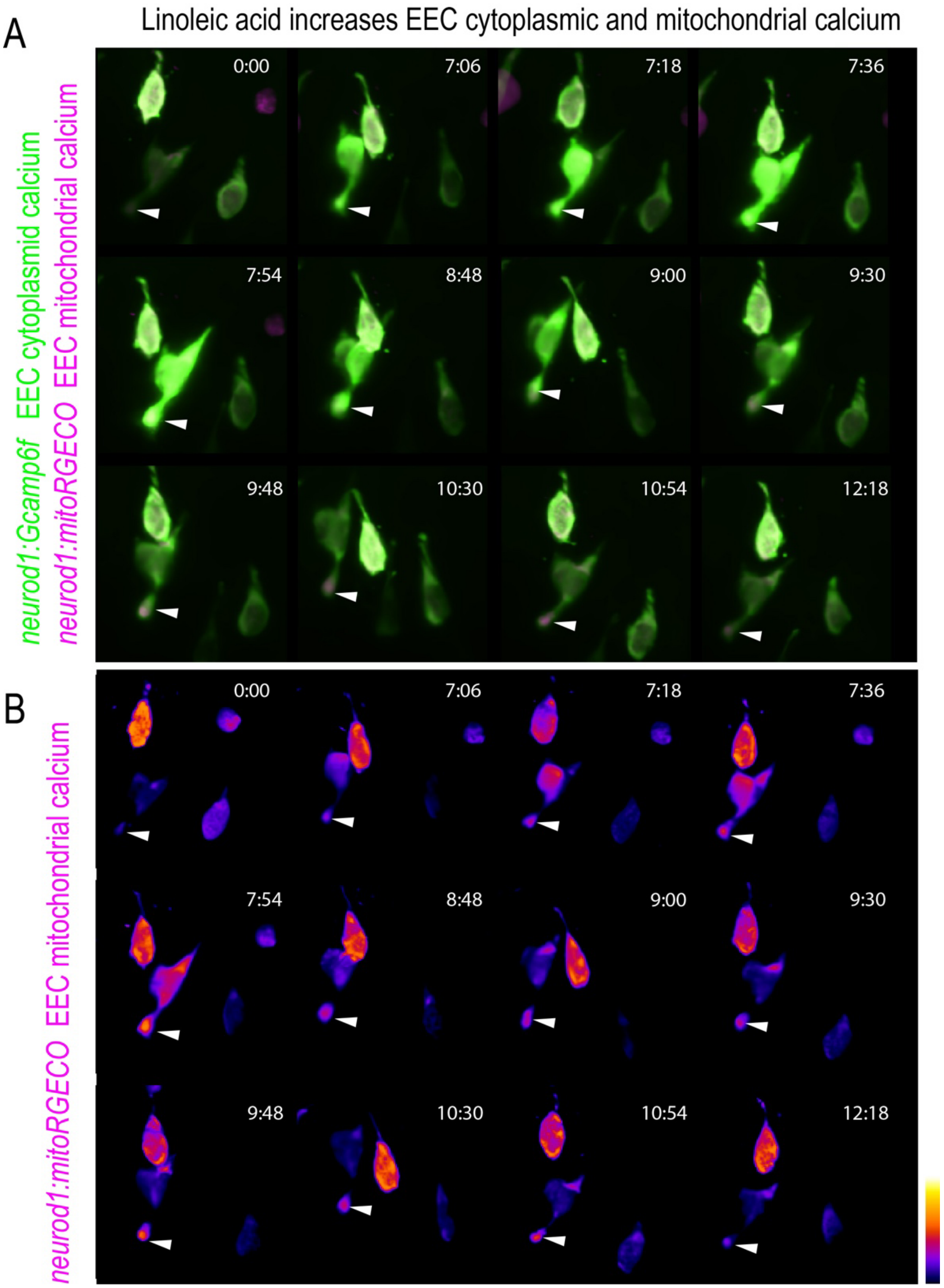

Fig. S14. Nutrient stimulation prominently promotes mitochondrial Ca^{2+} to arise near the basal membrane. (A-B) Zoom in view shows two linoleic acid activated EECs in *Tg(neurod1:Gcamp6f); Tg(neurod1:mitoRGECO)* zebrafish. The cytoplasmic calcium was indicated with Gcamp6f (green fluorescence), and the mitochondrial calcium was indicated with mitoRGECO (magenta fluorescence). White arrows show activated mitochondria near the basal membrane.

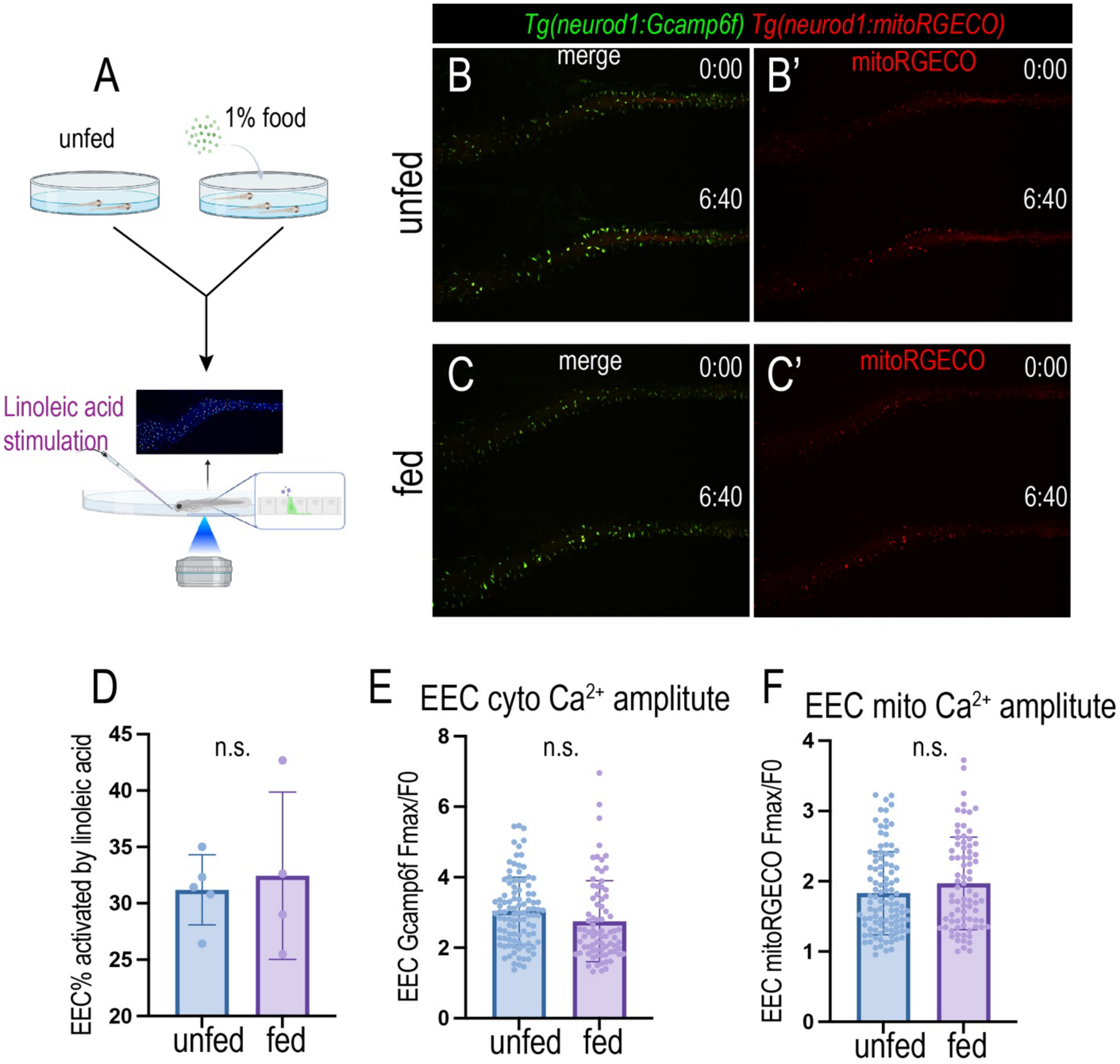

Fig. S15. Normal diet feeding does not affect EEC's cytoplasmic and mitochondrial Ca^{2+} in response to linoleic acid stimulation. (A) Zebrafish that were unfed and fed with normal food were stimulated with linoleic acid, and EECs' cytoplasmic and mitochondrial Ca^{2+} were imaged. For the fed group, 250μl of 1% food was added to the Petri dish from 4dpf to 6dpf. (B-C') Confocal projections of unfed and fed 7dpf *Tg(neurod1:Gcamp6f); Tg(neurod1:mitoRGECO)* zebrafish intestine at 0 min and 6:40 min post linoleic acid stimulation. (D) Quantification of EEC percentage that was activated by linoleic acid in unfed and fed zebrafish. Each dot represents an individual zebrafish. (E-F) The EECs' cytoplasmic Ca^{2+} amplitude and mitochondrial Ca^{2+} amplitude in unfed and fed zebrafish in response to linoleic acid stimulation. Each dot represents a linoleic acid activated EEC. EECs from 5 unfed and 4 fed zebrafish were analyzed. Unpaired, two-tailed Student's t-test was used in D-F.

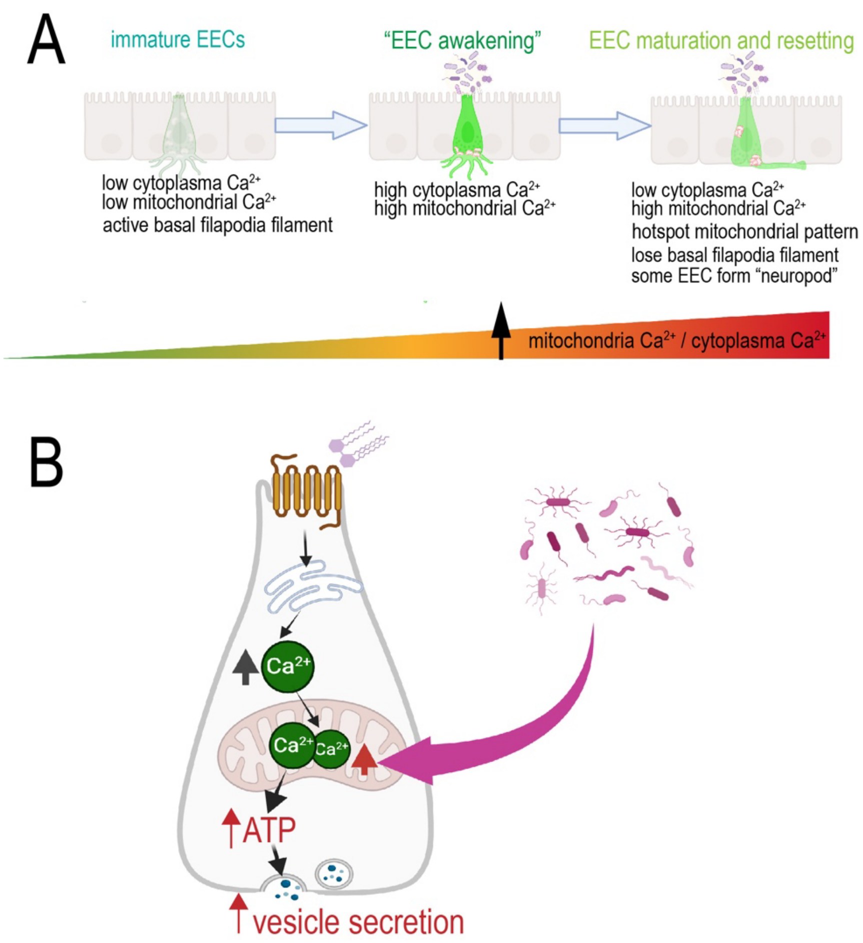

Fig. S16. Model figure showing gut microbiota modulate EEC maturation and mitochondrial function. (A) During early development, the immature EECs exhibit low cytoplasmic Ca^{2+} and low mitochondrial Ca^{2+} levels. These immature EECs have active filapodial filaments at the basal lateral membrane. Mitochondria are evenly distributed within the EECs. After the zebrafish hatched out and commensal microbiota started to colonize the zebrafish intestine, the EECs continued to develop and mature. Shortly after commensal microbiota colonization, the EECs increase both cytoplasmic and mitochondrial Ca^{2+} significantly ("EEC awakening"). After the EEC awakening, the EECs continue to mature and lose the basal lateral filapodial filaments. Some EECs form a neuropod. The mature EECs have low cytoplasmic Ca^{2+} but high mitochondria-to-cytoplasm Ca^{2+} ratio. Mitochondria in the mature EECs exhibit hotspot distribution pattern, and high mitochondria contents were accumulated near the base membrane. (B) Commensal microbiota promotes EEC mitochondrial respiration function and increases mitochondrial inner membrane electronic potential (ΔΨm). When nutrient stimulants, like fatty acids, stimulate the EECs, the EEC cytoplasmic Ca^{2+} rises. The high ΔΨm permits the cytoplasmic Ca^{2+} to flux into the mitochondrial matrix and power mitochondrial ATP production, which then promotes EEC vesicle release.

Development • Supplementary information

Table S1. The change of transcriptomics in GF and CV EECs.

Available for download at
https://journals.biologists.com/dev/article-lookup/doi/10.1242/dev.202544#supplementary-data

Table S2. The zebrafish lines and primary antibodies used in this manuscript.

Available for download at
https://journals.biologists.com/dev/article-lookup/doi/10.1242/dev.202544#supplementary-data

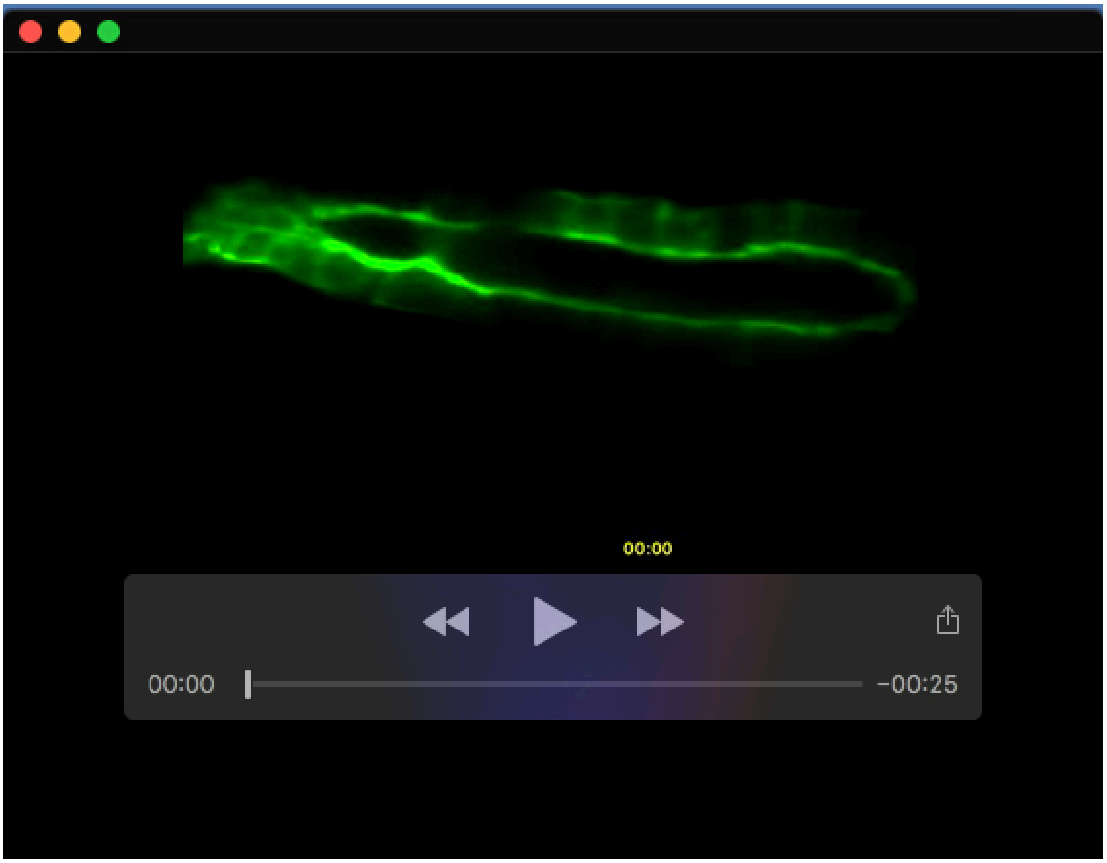

Movie 1. The 3dpf enterocytes do not have active filopodia filaments. The enterocyte actin was visualized via the *Tg(gata5:lifeActin-EGFP)* zebrafish line. The actin filaments were enriched in the brush border at the apical site.

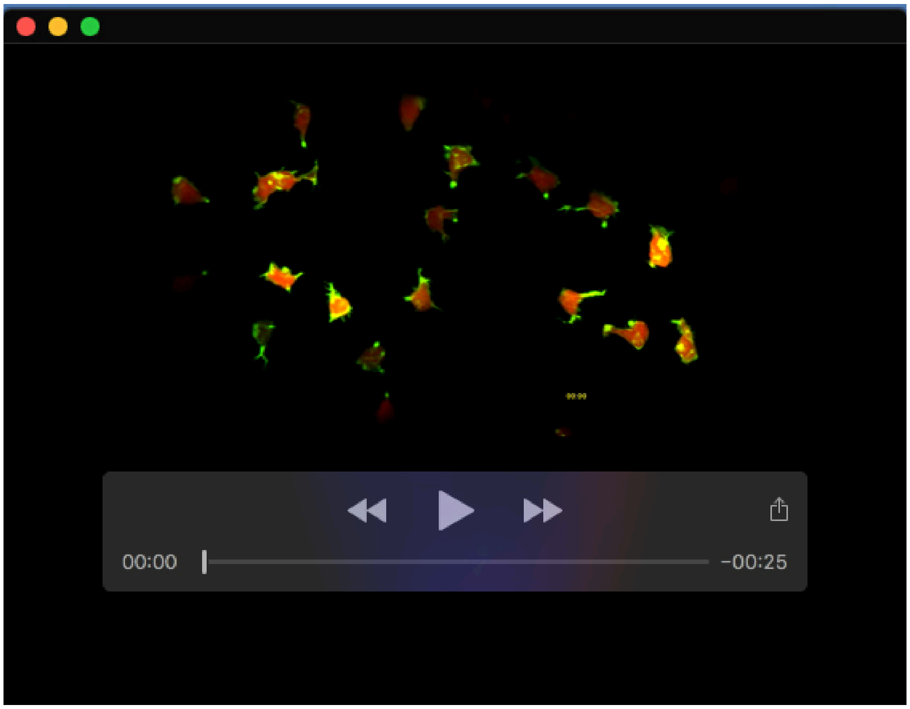

Movie 2. The active EEC filopodia filaments in 3dpf EECs. The EEC filopodia at the EEC base is visualized via the *Tg(neurod1:lifeActin-EGFP)* zebrafish line.

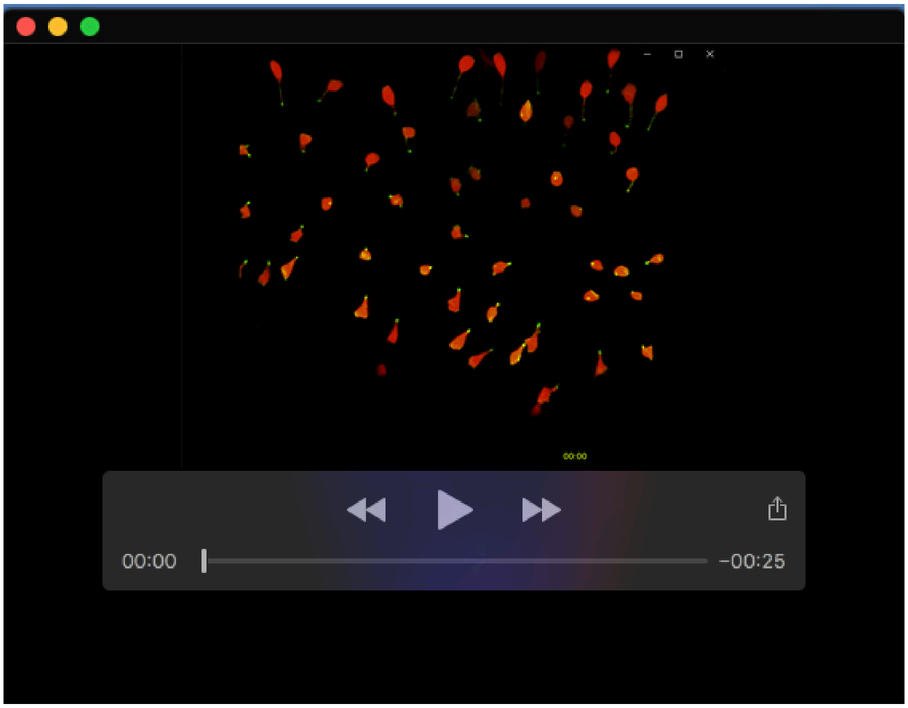

Movie 3. The 6dpf EECs do not display active filopodia structure at the base. The majority of the 6dpf EECs displayed enriched actin filaments at the apical brush border.

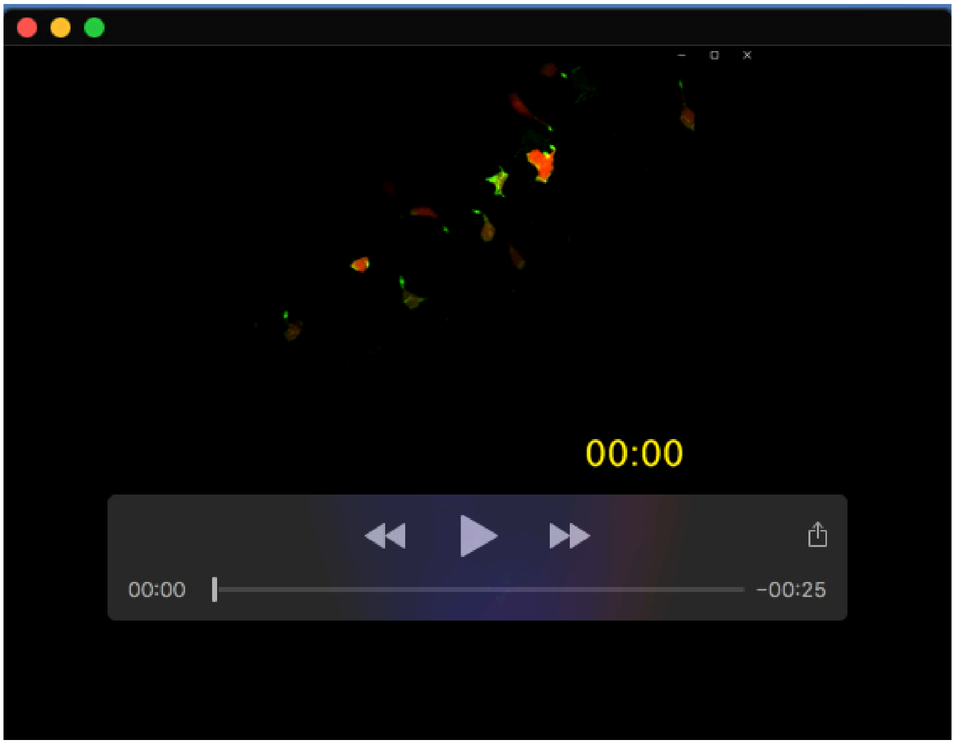

Movie 4. 7dpf GF zebrafish EECs contain active actin filaments.

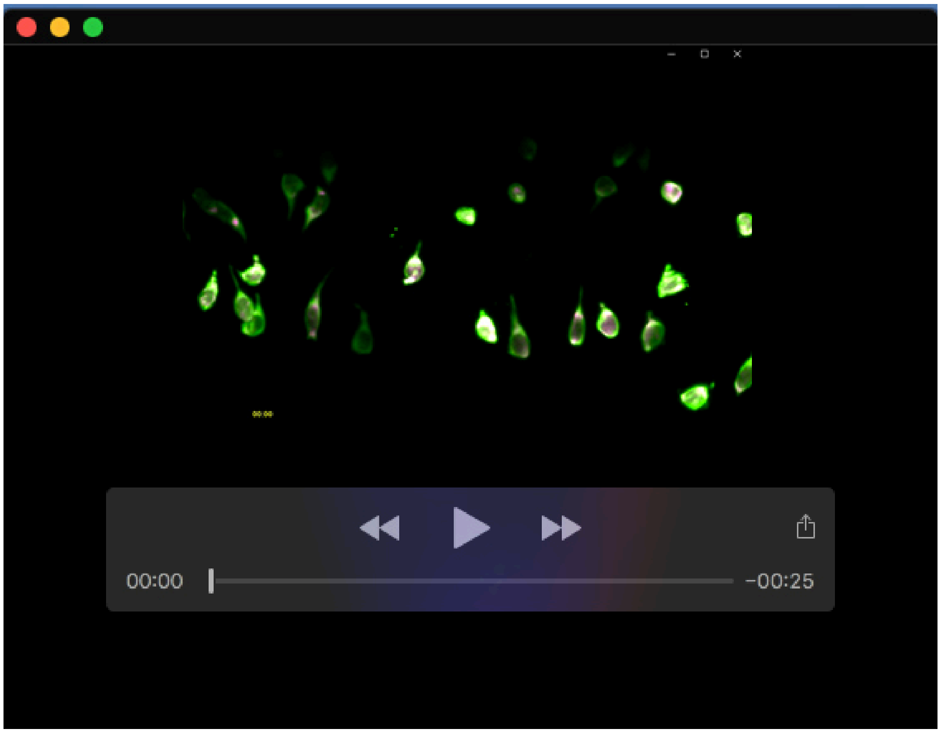

Movie 5. Use of the *Tg(neurod1:Gcamp6f); Tg(neurod1:mitoRGECO)* to simultaneously image the EECs' cytoplasmic and mitochondrial calcium.

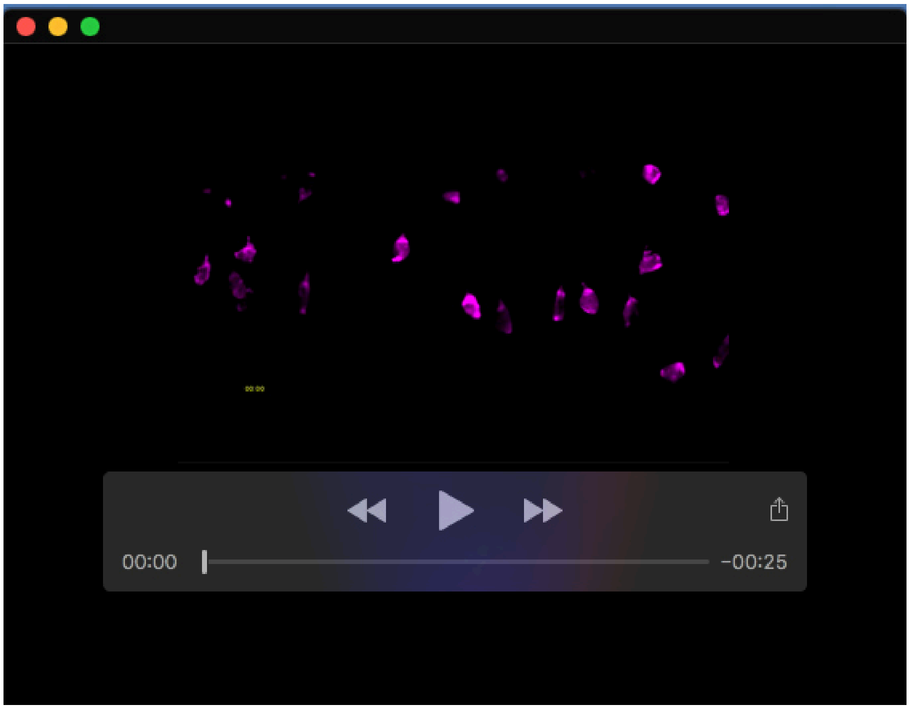

Movie 6. The dynamic of the EEC mitochondrial calcium at the resting conditions in conventionally raised zebrafish.

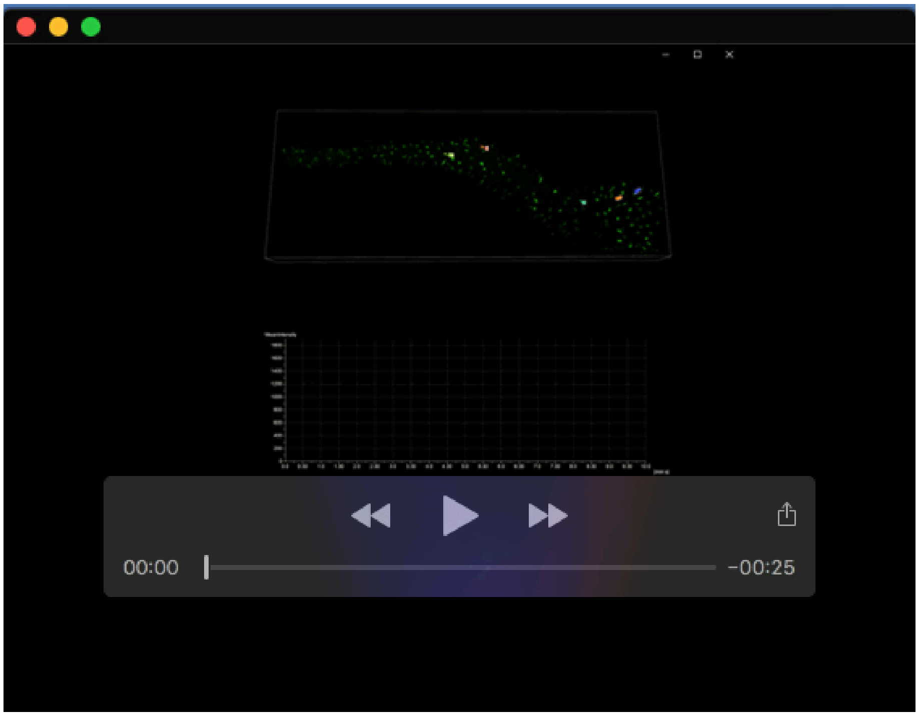

Movie 7. 3D objective tracking for selective EECs in a zebrafish sample that is stimulated with linoleic acid.

Development • Supplementary information

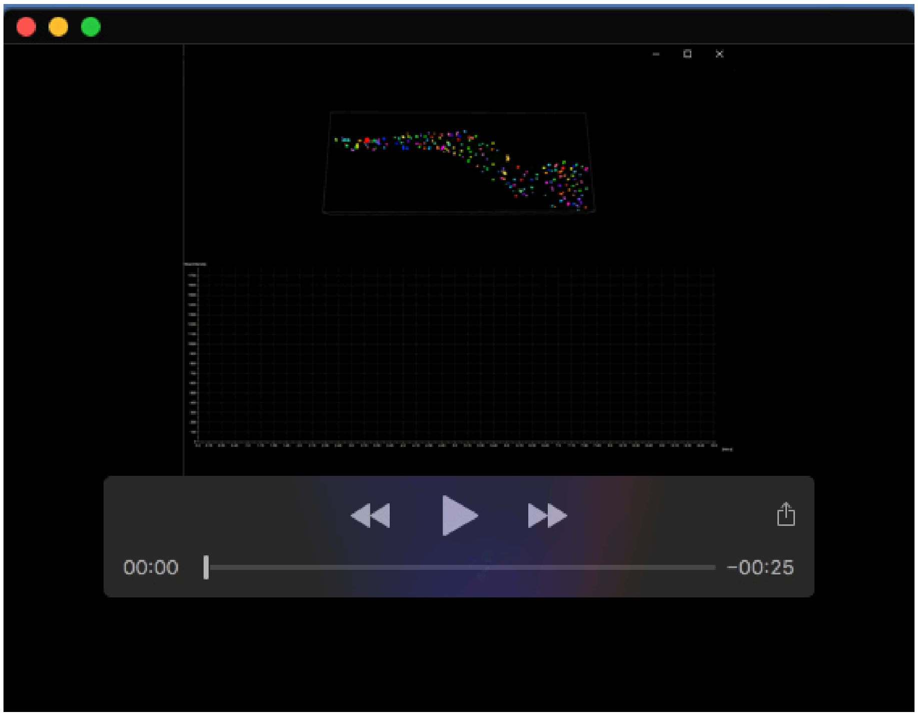

Movie 8. 3D objective tracking for all the EECs in a zebrafish sample that is stimulated with linoleic acid. Plot profiles for two selected EECs were shown.

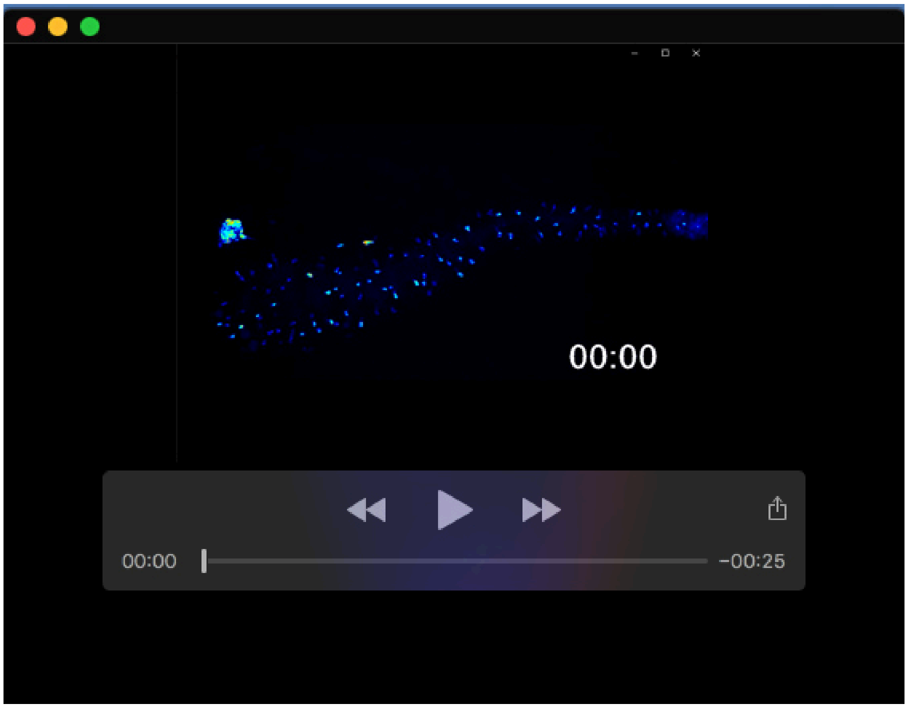

Movie 9. The spontaneous calcium fluctuations in conventionalized (CV) zebrafish EECs. The EEC calcium dynamics were visualized using the *Tg(neurod1:Gcamp6f)* zebrafish.

Development • Supplementary information

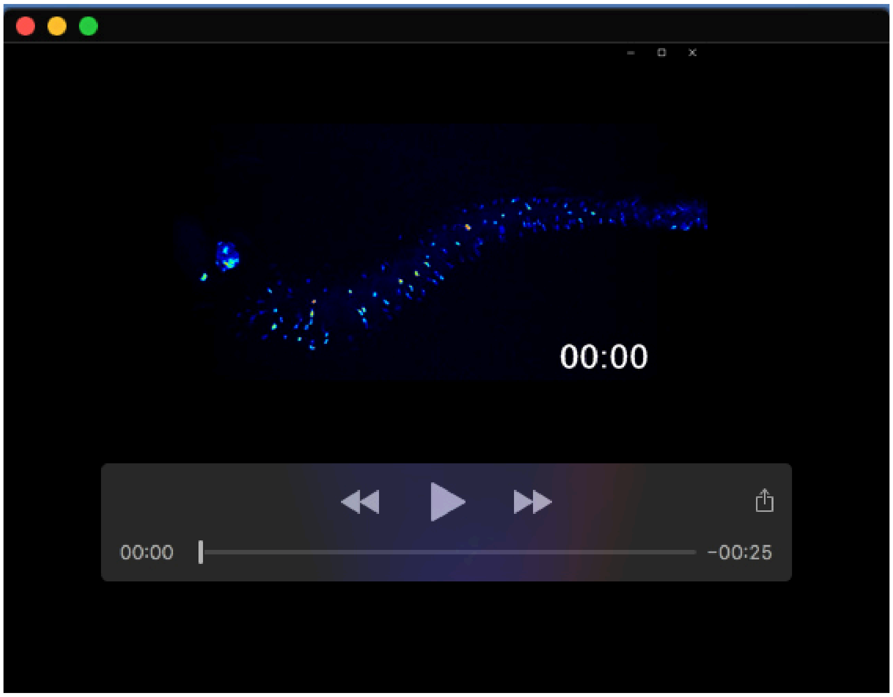

Movie 10. The reduced calcium fluctuations in germ-free zebrafish EECs. The EEC calcium dynamics were visualized using the *Tg(neurod1:Gcamp6f)* zebrafish.

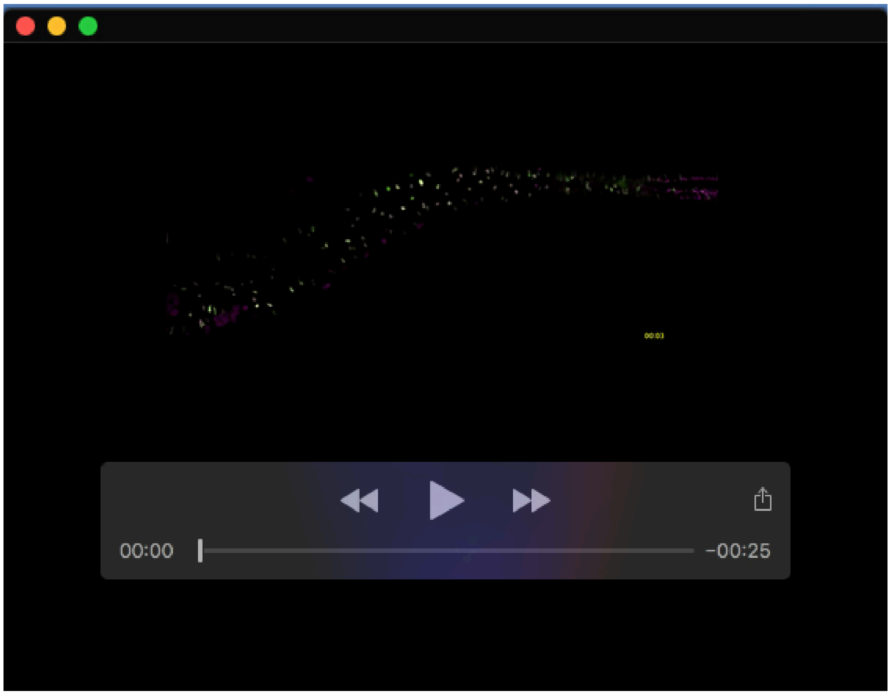

Movie 11. Linoleic acid stimulation increases EEC cytoplasmic and mitochondrial calcium in conventionally raised zebrafish. The cytoplasmic calcium was visualized via the *Tg(neurod1:Gcamp6f)* (green) and the mitochondrial calcium was visualized via the *Tg(neurod1:mitoRGECO)* (magenta).

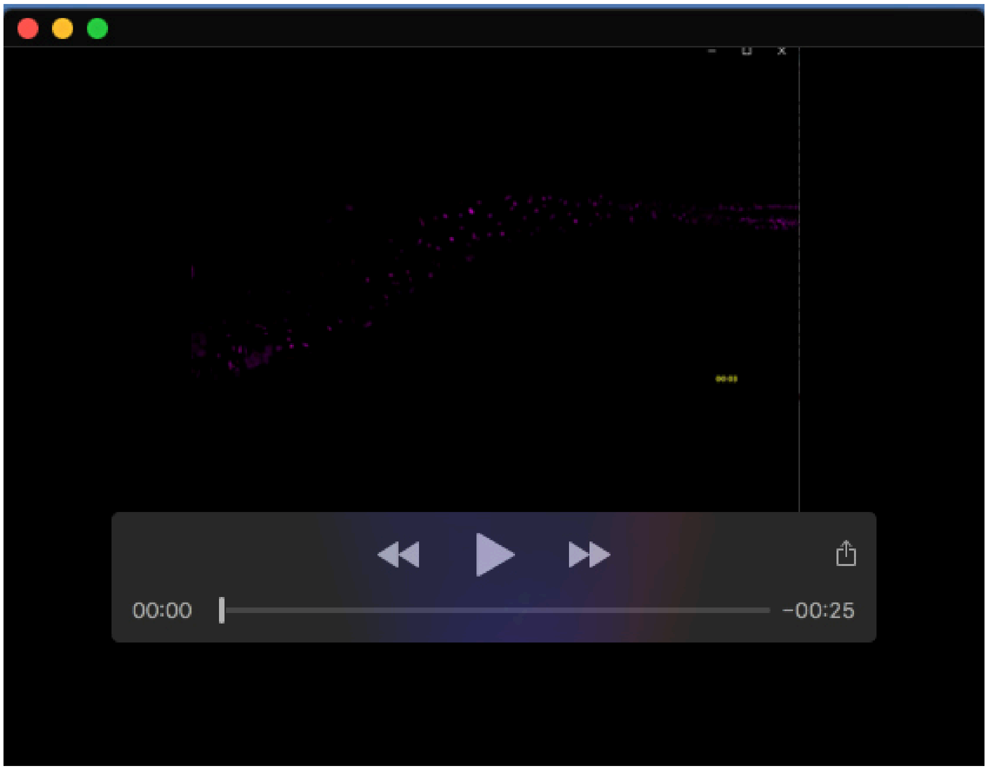

Movie 12. Linoleic acid stimulation increases EEC mitochondrial calcium in conventionally raised zebrafish.

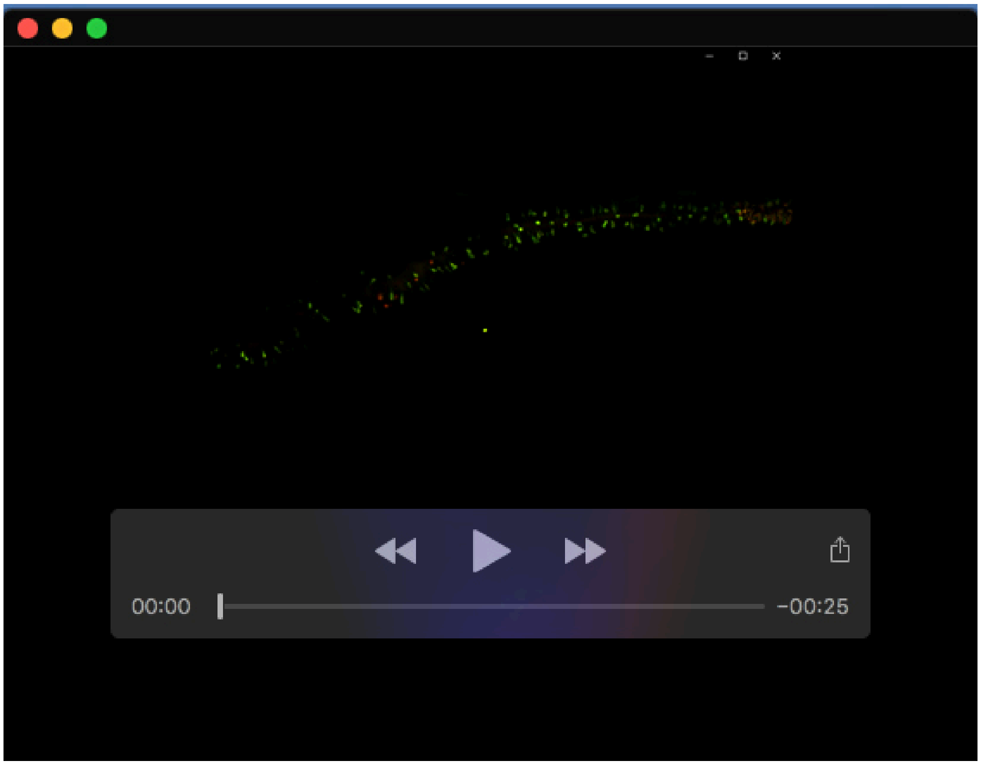

Movie 13. Glucose stimulation increases EECs' cytoplasmic and mitochondrial calcium in conventionally raised zebrafish.
