# Supplementary material for: Gut microbiota regulate maturation and mitochondrial function of the nutrient-sensing enteroendocrine cell
Source: Development. 2024 Apr 30;151(8):dev202544. doi: 10.1242/dev.202544 (PMC11112165; doi:10.1242/dev.202544)
Supplement: Supplementary file 1 [file develop-151-202544_review_history.pdf]

Gut microbiota regulate maturation and mitochondrial function of the nutrient-sensing enteroendocrine cell

Alfahdah Alsudayri, Shane Perelman, Melissa Brewer, Annika Chura, Madelyn McDevitt, Catherine Drerup and Lihua Ye
DOI: 10.1242/dev.202544

Editor: James Wells

Review timeline
Original submission: 14 November 2023
Editorial decision: 27 December 2023
First revision received: 5 March 2024
Accepted: 25 March 2024

Original submission

First decision letter

MS ID#: DEVELOP/2023/202544

MS TITLE: Gut microbiota regulates the nutrient sensing enteroendocrine cell maturation and mitochondrial function

AUTHORS: Alfahdah Alsudayri, Shane Perelman, Annika Chura, Melissa Brewer, Madelyn McDevitt, Catherine Drerup, and Lihua Ye

I have now received all the referees' reports on the above manuscript, and have reached a decision. The referees' comments are appended below, or you can access them online: please go to BenchPress and click on the 'Manuscripts with Decisions' queue in the Author Area.

As you will see, the referees express considerable interest in your work, but have some significant criticisms and recommend a substantial revision of your manuscript before we can consider publication. If you are able to revise the manuscript along the lines suggested, which may involve further experiments, I will be happy receive a revised version of the manuscript. Your revised paper will be re-reviewed by one or more of the original referees, and acceptance of your manuscript will depend on your addressing satisfactorily the reviewers' major concerns. Please also note that Development will normally permit only one round of major revision. If it would be helpful, you are welcome to contact us to discuss your revision in greater detail. Please send us a point-by-point response indicating your plans for addressing the referees' comments, and we will look over this and provide further guidance.

Please attend to all of the reviewers' comments and ensure that you clearly highlight all changes made in the revised manuscript. Please avoid using 'Tracked changes' in Word files as these are lost in PDF conversion. I should be grateful if you would also provide a point-by-point response detailing how you have dealt with the points raised by the reviewers in the 'Response to Reviewers' box. If you do not agree with any of their criticisms or suggestions please explain clearly why this is so.

Reviewer 1

Advance summary and potential significance to field

© 2024. Published by The Company of Biologists under the terms of the Creative Commons Attribution License (https://creativecommons.org/licenses/by/4.0/).

Alsudayri et al suggest the microbiome as a key factor in the early postnatal (first few days of enteral feeding) maturation of EECs. The authors compare markers of EEC maturity such as shape, cytoskeletal rearrangements, and mitochondrial activity in 6-7dpf zebrafish raised in germ-free conditions and those colonized with conventional microbiome at 3dpf, the time point at which zebrafish begin to take in enteral nutrients instead of the yolk. While the genetic tools and imaging are beautiful, my main feeling is that some of the data seems cherry-picked and non-representative, and the conclusions are overstated. Addressing the following comments would greatly increase my enthusiasm for the work, which is otherwise a great fit for Development and significant, timely, and important to the field.

Comments for the author

Big concerns:

1. In Figure 3, the authors present images and quantification of the actin rearrangement from basal filaments at 3dpf to an apical concentration by 7dpf. The authors conclude that in GF animals, this process is disrupted; however, ~85% of counted GF EECs successfully rearranged their cytoskeleton by 7dpf, compared to ~93% in conventionalized fish (Figure 3G). While this finding was statistically significant, I question whether it is biologically significant. Many GF fish have few EECs with few retained basal actin filaments whereas many CV fish display GF-levels of retained actin filaments. The authors do not mention the possibility of a slight developmental delay in GF animals; at later timepoints or adult stages, do a small percentage of EECs persist in an immature state in the absence of microbes (supporting the current conclusion), or do the GF fish eventually catch up? For example, actin-GFP distribution looks very similar between 6dpf CV and 7dpf GF animals (Figure 3).

2. Similar to point 1, the authors are often unclear about the age of the animal (sequencing experiments in 1 and S1), and region of the intestine analyzed. While the markers used to enrich for EECs by FACS are in the methods, they should also be in the main text and figure legend. The main EEC driver in this study is Neurod1.
Is it possible that expression of Neurod1, and therefore fidelity of all downstream experiments, could be affected by the maturation state of EECs – and reflecting a developmental delay? In Figure S3, the authors found no difference in mitochondrial abundance in proximal intestine, only distally. However, the line of text (254) states that “However, at 6dpf, EECs exhibit higher mitochondrial abundance compared with 3dpf-5dpf EECs” without referencing region. Disclosure of which region of the intestine is analyzed in each experiment is essential.

3. Often, the authors ask the readers to trust that they have identified the apical and basal sides of a cell without context. Adding a merged image with the lumen demarcated, DAPI, or an epithelial marker would greatly improve our ability to trust the conclusions of these studies – especially as polarity is a major conclusion. In the zoomed-out images, like 3E, the bright hotspot of actin GFP expression is pointing in different directions in each EEC, not all pointing to an apical/luminal surface. A counterstain would also assist in visualizing the neuropods.

4. I suggest restructuring the manuscript such that Figure 3 is presented first. This section of text (around line 200) is essential information that would have been useful in the introduction or first part of the Results section. Moreover, starting with the observation that over the first few days of enteral feeding EECs rearrange their cytoskeleton such that the actin bundle points apically gives rationale for looking at lumenal factors influencing their maturation, such as nutrients or microbes.

5. Have the authors considered the sequence of events in postnatal EEC maturation? Does actin rearrangement and, presumably, the concentration of nutrient and microbial receptors on the apical surface come first? Or, do mitochondrial dynamics drive the rearranged distribution of actin and of mitochondrial-rich regions? Looking more closely at 4dpf may be informative.

6. What is the composition of food the fish are eating between 3dpf and 7dpf? Are they exposed to any LCFAs prior to the linoleic acid challenge? Would you see a more robust response if the animals were fasted?
Do they have a similar response to a glucose challenge?

© 2024. Published by The Company of Biologists under the terms of the Creative Commons Attribution License (https://creativecommons.org/licenses/by/4.0/).

7. Are the immature EECs in the GF fish mapping back to any of the subtypes identified in S1? Is there a particular population that requires microbial stimulation, and are there any candidates for required receptors that mediate microbial signals in the GF EECs that fail to mature?

Smaller concerns

1. The introduction does not adequately detail the intestinal development of organisms that feed off yolk – like the animals used in this study.
2. The pyyb gene encodes peptide YY; however, in the text (116) the authors say it is pancreatic polypeptide B. I think this is just a typo in the text, but it is interesting that PYY expression was found enriched proximally, when it is enriched distally in mammals. Pancreatic polypeptide (PP) should be enriched proximally, hence my confusion.
3. Quantifications in Figure 1 are not consistent – why are some hormones counted as a percent, and others as cell number? How were these counts normalized (ie, length of intestine, total cell number, etc)?
4. In Figure 4, the distribution of mitochondrial location should also be counted in the GF animals. As it is only 5 CV EECs were counted for Figure 4E. How did the authors choose which cells to count, as there is even a difference in CV EEC mitochondrial localization shown in 4C and 4G. Since ~85% (Figure 3) to ~89% (Figure 4H) of GF EECs appear to mature normally, how will the authors ensure that any GF EEC mitochondria counted will be representative?
5. After stimulation (Figure 7), how long is the increased mitochondrial Ca++ signal maintained? Figure 7H suggests a quick drop, but does not extend the time long enough to see if it ever returns to baseline. What happens after a second stimulation?

<u>Reviewer 2</u>

Advance summary and potential significance to field

The manuscript by Ye and colleagues entitled: “Gut microbiota regulates the nutrient sensing enteroendocrine cell maturation and mitochondrial function” reports exciting new insights into the calcium dynamics of enteroendocrine cells (EECs) as they mature, respond to gut microbes, and respond to dietary stimuli. This study is the first to describe calcium dynamics, including changes in mitochondrial calcium dynamics, in the EECs of living, developing animals.

Comments for the author

Major comments:

1. Last statement of abstract: “Selectively manipulating gut microbial signals to alter EEC mitochondrial function will provide new opportunities to change gut-brain nutrient sensing efficiency and feeding behavior” would better belong in the discussion. The manuscript does not discuss gut-brain signaling or feeding behavior.

2. The authors report cutting edge in vivo analysis of enteroendocrine cells but a limitation of the study is the lack of clarity about the extent to which the reported data are representative across independently performed experiments.

This is important because the maturation of the EECs is evidently dependent on the microbiota, which will establish somewhat differently between independently reared clutches of zebrafish. The authors are describing new cell biological phenomena and it is important for them to document the data in such a way that the reader can evaluate sources of biological variance. For example: are EECs from the same individual fish more similar than EECs from different fish from the same clutch? From different clutches? Are there rostral to caudal differences in EECs maturation (as for example is seen with the maturation of enteric neurons)? In the graphs, it would be helpful to use color or symbol designations to indicate EECs from the same versus different individuals from the same or different clutches. Specific comments about the figures:

Figure 3G: Clarify how many total EECs were quantified per individual fish.

© 2024. Published by The Company of Biologists under the terms of the Creative Commons Attribution License (https://creativecommons.org/licenses/by/4.0/).

Clarify how many independently derived clutches of zebrafish were tested and whether there was clutch to clutch variation.

Figure 4E: It is not obvious how the quantification for the 3 dpf mitochondrial distribution matches the images. Shouldn't the mitochondrial intensity diminish through the nucleus? The quantification was done for 5 EEC cells from one zebrafish at the two time points. Was this one fish representative of other individuals, especially considering different individuals from different clutches with different microbiotas?

Figure 4H: Clarify how many total EECs were quantified per individual fish.

Figure 4 H & I: Clarify how many independently derived clutches of zebrafish were tested and whether there was clutch to clutch variation.

Figure 5F-H: Provide quantification of a single fish for each time point. Are these measurements representative of other individuals? Is there rostral to caudal variation in the EEC values quantified?

Figure 5I-K: Clarify the providence of the 10 EECs quantified. Are they from one individual? Multiple individuals? Multiple clutches?

Figure 6D-F: states that more than 5 zebrafish were quantified for each condition, but does not clarify whether they were fish from the same or different clutches.

Figure 6G-M: clarify whether these are EECs from a single or multiple zebrafish.

Figure 7: The end of the figure legend states that more than 100 EECs from at least 3 zebrafish were analyzed, but it is not clear to which parts of the figure this statement applies.

Figure 8: Clarify which EECS correspond to which individual zebrafish and whether zebrafish from multiple independent clutches were analyzed.

3. The image analysis method by which EECs' shapes and signal intensities were quantified are crucial for evaluating the data reported, but insufficient information is provided. The methods refer to the Nikon NLS element software.

How does this software segment the elements? To what extent is this user-specified? A supplemental figure providing more details about the cellular segmentation approach would be valuable.

4. As a proof of concept that the calcium distributions reported in EECs are accurately measured, the authors should include some pharmacological manipulations that alter these distributions, for example acute treatment with a mitochondrial membrane depolarizing agent that would be expected to disrupt mitochondrial calcium uptake.

Minor comment:

Line 36: jarring switch from "all organisms" to "during development, the fetus…": need to clarify that the second statement is about placental mammal development, not all organisms. It is confusing to introduce the EECs in the context of mammals when the paper will study them in non-mammals.

<u>Reviewer 3</u>

Advance summary and potential significance to field

This manuscript analyzes how mitochondrial activity in EECs changes from day 3-day 6 in zebrafish, and examine how this compares to germfree zebrafish. Most of the figures (aside from figure 1 as questioned below) seems to support that colonization broadly induces EEC maturation and mitochondrial response in the presence of the long-chain fatty acid linoleic acid. Overall, the

© 2024. Published by The Company of Biologists under the terms of the Creative Commons Attribution License (https://creativecommons.org/licenses/by/4.0/).

general examination of EEC mitochondrial responses seem sound and consistent with EEC maturation over time. While the studies then testing dependence of these changes on colonization also generally align with the author's interpretation, addressing points below would help strengthen conclusions and biologic depth.

Comments for the author

1. Figure 1 suggests that some subsets of EECs are decreased following commensal microbiota colonization -Gcg/GLP-1+EECs in the proximal intestine and Trpa1+EECs in the distal intestine (Fig. 1F-I, L-N), whereas others are unchanged. However the transcriptional analyses in Figure 2 support instead an increase in EEC specific gene expression profiles (Fig.2C- 74.5% of upregulated genes are enriched in EECs). How are these differences reconciled? It would seem that promoting EEC maturation would be reflected as an increase in EEC numbers?

2. Can the localization of microbiota along the length of the intestine be demonstrated in relation to changes in observed in Figure 1, during the 3-4 days between colonization to analyses?

3. The study thoroughly examines mitochondrial activity from day 3-6 dpf in zebrafish (Fig. 5). The assumption is that these data are representative of colonized zebrafish, although not clearly designated as CV. The comparison between GF and CV in the next figure lacks the same level of depth in evaluation. Its not clear why the y axes values differ between Fig. 6 and Fig.

5 for CV. Also the differences shown in Fig 6D-F are very small despite the stars for stats indicating significance in EEC cytoplasm and mitochondria calcium activity. The issues are similar comparing Fig. 7 and Fig. 8. These points should be addressed to assess technical and biological significance of these studies in relation to the microbial colonization.

First revision

Author response to reviewers' comments

Reviewer 1 Advance Summary and Potential Significance to Field:

Alsudayri et al suggest the microbiome as a key factor in the early postnatal (first few days of enteral feeding) maturation of EECs. The authors compare markers of EEC maturity such as shape, cytoskeletal rearrangements, and mitochondrial activity in 6-7dpf zebrafish raised in germ-free conditions and those colonized with conventional microbiome at 3dpf, the time point at which zebrafish begin to take in enteral nutrients instead of the yolk. While the genetic tools and imaging are beautiful, my main feeling is that some of the data seems cherry-picked and non-representative, and the conclusions are overstated. Addressing the following comments would greatly increase my enthusiasm for the work, which is otherwise a great fit for Development and significant, timely, and important to the field.

We thank the reviewer for constructive comments and feedback. During this revision, we have conducted extensive experiments and text revision to address the reviewer's concern. Below please find our detailed response to each point.

Reviewer 1 Comments for the Author:

Big concerns:

1. In Figure 3, the authors present images and quantification of the actin rearrangement from basal filaments at 3dpf to an apical concentration by 7dpf. The authors conclude that in GF animals, this process is disrupted; however, ~85% of counted GF EECs successfully rearranged their cytoskeleton by 7dpf, compared to ~93% in conventionalized fish (Figure 3G). While this finding was statistically significant, I question whether it is biologically significant. Many GF fish have few EECs with few retained basal actin filaments, whereas many CV fish display GF-levels of retained actin filaments.

© 2024. Published by The Company of Biologists under the terms of the Creative Commons Attribution License (https://creativecommons.org/licenses/by/4.0/).

The authors do not mention the possibility of a slight developmental delay in GF animals; at later timepoints or adult stages, do a small percentage of EECs persist in an immature state in the absence of microbes (supporting the current conclusion), or do the GF fish eventually catch up? For example, actin-GFP distribution looks very similar between 6dpf CV and 7dpf GF animals (Figure 3).

We thank the reviewer for the constructive comments. As the reviewer points out, our data suggest that actin rearrangement is one of the cellular processes involved in EEC maturation. This cellular process is likely regulated by both intrinsic signals and extrinsic environmental factors. Our data suggest that gut microbiota colonization is one of the factors (but not the only factor) that promote this process. This conclusion is supported by RNAseq data in **Figure 3G** (Figure 2G in the previous submission). It is also supported by our examination using the *Tg(neurod1:lifeActin-EGFP)* model. Experiments using live imaging and fixed tissue staining support the conclusion that the proximal intestinal EECs display higher active actin filaments in the absence of commensal microbiota (See new data in **Figure 1 G-K, Figure S2, and video 4**). In each of these independently repeated experiments, we observe a higher percentage of EECs in GF zebrafish proximal intestine display active actin filaments at the base. Consistently, our data supports the conclusion that commensal microbiota colonization promotes EECs' actin remodeling. How EECs' actin remodeling affects physiology remains an important question that can be addressed by future studies.

It is known that the development process differs in different organ systems in GF animals, including zebrafish. However, we could not find literature to support the conclusion that GF animals/zebrafish display a slight development delay. To address the reviewer's concern regarding the possibility of a slight developmental delay in GF animals, during this revision, we followed the reviewer's suggestion and conducted new gnotobiotic experiments in a later stage (it is currently technically impossible to create GF zebrafish beyond 1 month and GF zebrafish maintaining beyond 10 days will require a completely different feeding and caring procedure). EECs' actin filaments were analyzed in 9dpf GF and CV zebrafish (see **new data in Figure S3**, result section Line 145-147). Our data suggest that consistent with our observation for 7dpf GF and CV zebrafish, the EECs in 9dpf GF zebrafish proximal intestine displayed higher actin filaments at the base compared with CV EECs (see **new data in Figure S3**). This data supports our current conclusion that gut microbiota promotes EEC actin remodeling and maturation. In the absence of commensal microbiota, a small percentage of EECs may persist in an immature state.

2. Similar to point 1, the authors are often unclear about the age of the animal (sequencing experiments in 1 and S1), and region of the intestine analyzed. While the markers used to enrich for EECs by FACS are in the methods, they should also be in the main text and figure legend. The main EEC driver in this study is Neurod1. Is it possible that expression of Neurod1, and therefore fidelity of all downstream experiments, could be affected by the maturation state of EECs - and reflecting a developmental delay? In Figure S3, the authors found no difference in mitochondrial abundance in proximal intestine, only distally. However, the line of text (254) states that "However, at 6dpf, EECs exhibit higher mitochondrial abundance compared with 3dpf-5dpf EECs" without referencing region. Disclosure of which region of the intestine is analyzed in each experiment is essential.

We thank the reviewer's comment. We have now added the age of the animal and the region of the intestine analyzed in every figure legend and the main text. Majority of the data in the article focuses on the proximal intestine, a conserved intestinal region important for nutrient processing and EECs' nutrient sensing. We have also updated the marker used for enriching EECs by FACS in the main text (Result section line 207-211 and figure 3 legend).

The neurod1 is a marker used by many previous studies to label both immature and mature EECs (Gehart et al., 2019). To address the reviewers' concerns about whether neurod1 expression is affected by the EEC maturation status, we analyzed the neurod1:EGFP green fluorescence intensity in EECs at 3dpf and 6dpf zebrafish. Our data suggested that 3dpf and 6dpf EECs display the same EGFP fluorescence level (**see new data in Figure S6 and in the result section line 192-195**). Our data suggests that the *neurod1* expression is not affected by the EECs' maturation state.

Regarding the reviewers' concern regarding Figure S3 (now Figure S7), we apologize for the confusion. The text reviewer points out, "However, at 6dpf, EECs exhibit higher mitochondrial abundance compared with 3dpf-5dpf EECs," referring to the proximal intestine EECs. The data in

© 2024. Published by The Company of Biologists under the terms of the Creative Commons Attribution License (https://creativecommons.org/licenses/by/4.0/).

Figure S3B (now Figure S7B) indicate that the proximal intestinal EEC mitochondrial abundance is not affected by gut microbiota colonization. Collectively, this data suggests that gut microbiota do not regulate mitochondria biogenesis in the proximal intestinal EECs but affect mitochondrial distribution in EECs. We have now updated figure legend for Figure S7 and the text in the results section to clarify (Line 278-279).

3. Often, the authors ask the readers to trust that they have identified the apical and basal sides of a cell without context. Adding a merged image with the lumen demarcated, DAPI, or an epithelial marker would greatly improve our ability to trust the conclusions of these studies – especially as polarity is a major conclusion. In the zoomed-out images, like 3E, the bright hotspot of actin GFP expression is pointing in different directions in each EEC, not all pointing to an apical/luminal surface. A counterstain would also assist in visualizing the neuropods.

We thank the reviewer's comments. In our previous submission, the images presented in Figure 3E-F are from live imaging and therefore, we could not add DAPI or another immunofluorescence marker. To address the reviewer's concern, during this revision, we conducted three independent new gnotobiotic experiments in 7dpf GF and CV zebrafish, and the samples were fixed and stained with DAPI (**New Figure 1 G-K, New figure S2 A-E**). The intestinal lumen was now labeled in the new figure. Zoom-out views of the proximal intestine of representative GF and CV zebrafish are presented (**New Figure 1 G, I and Figure S2 A, D**). In addition, zoom-in views of the representative EECs in GF and CV zebrafish proximal intestine are also presented (**New Figure 1 H, J and Figure S2 B-C, E**) showing the EEC Actin filaments in the GF zebrafish EECs.

The data presented in the original Figure 3E-F were now moved to **Figure S2 F-J**. In this data, the whole intestine was imaged, and the data were presented as z-projections. Due to the shape of the intestinal tube, the EECs in the intestinal wall were facing different directions. For some EECs (like the EECs in old Figure 3E-F), the apical side was facing inward, and the basal side was displayed for those EECs, which showed the actin filaments go in different directions. In the **new Figure S2F-J**, we generated a 3D annotation of the same intestine region and re-arranged the orientation to let the apical side of those EECs face up, which enables better visualization of the actin filaments at the EECs' base in GF zebrafish EECs (**New Figure S2G, G', H**).

4. I suggest restructuring the manuscript such that Figure 3 is presented first. This section of text (around line 200) is essential information that would have been useful in the introduction or first part of the Results section. Moreover, starting with the observation that over the first few days of enteral feeding EECs rearrange their cytoskeleton such that the actin bundle points apically gives rationale for looking at lumenal factors influencing their maturation, such as nutrients or microbes.

We thank the reviewer's comments and suggestions. We have now arranged our figures and text. The figure 3 and associated text are now presented first.

5. Have the authors considered the sequence of events in postnatal EEC maturation? Does actin rearrangement and, presumably, the concentration of nutrient and microbial receptors on the apical surface, come first? Or, do mitochondrial dynamics drive the rearranged distribution of actin and of mitochondrial-rich regions? Looking more closely at 4dpf may be informative.

NOTE: We have removed unpublished data that had been provided for the referees in confidence.

Figure 1. Relationship between EECs' actin filaments and mitochondrial calcium in 4dpf zebrafish. (A-A') Confocal projection of 4dpf *Tg(neurod1:lifeActin-EGFP); Tg(neurod1:mitoRGECO)* zebrafish proximal intestine. Live zebrafish were imaged. (B-B') Zoom-in views showing two EECs with active actin filaments that exhibit high mitoRGECO fluorescence signal. (C) Quantification of mitoRGECO fluorescence intensity for EECs with and without active actin filaments. 35 EECs with actin filaments and 82 EECs without actin filaments from 7 zebrafish proximal intestine were analyzed. Student T-test was used in C. **** P<0.0001.

We thank the reviewer's comments and suggestions. We followed the reviewer's suggestion and examined 4dpf *Tg(neurod1:lifeActin- EGFP);Tg(neurod1:mitoRGECO)* zebrafish EECs. We reasoned that if EECs' actin filament rearrangement happens before mitochondrial activation,

© 2024. Published by The Company of Biologists under the terms of the Creative Commons Attribution License (https://creativecommons.org/licenses/by/4.0/).

EECs with active actin filaments will be expected to display lower mitochondrial calcium activity. However, if mitochondrial activation happens before EECs' actin filament rearrangement, EECs with active actin filaments may display high mitochondrial calcium activity. The new results on the left support the latter hypothesis.

This result, however, is not included in our revised manuscript because: To rigorously test the sequence of events in postnatal EEC maturation and the relationship between EECs' actin rearrangement and mitochondrial activity, it requires extensive fine temporal tracing between 3dpf and 4dpf. In addition, pharmaceutical and genetic methods are required to manipulate EECs' actin dynamic and mitochondrial metabolism to determine the sequential event and relationship between EECs' actin dynamic and mitochondrial function. The genetic tools to manipulate EECs' actin dynamics and mitochondrial function are currently unavailable. Therefore, such extensive investigation to determine the relationship between EECs' actin rearrangement and mitochondrial activity is beyond the scope of the current manuscript. The questions raised by the reviewer can be rigorously tested and answered in future studies.

In this revised manuscript, we added new text in the Discussion section (Line 493- 502) to discuss the relationship between EECs' actin rearrangement and mitochondrial function and the literature evidence which supports the hypothesis that increased mitochondrial metabolism promotes cellular maturation.

6. What is the composition of food the fish are eating between 3dpf and 7dpf? Are they exposed to any LCFAs prior to the linoleic acid challenge? Would you see a more robust response if the animals were fasted? Do they have a similar response to a glucose challenge?

The gnotobiotic zebrafish (housed in 50ml water) were fed 250 µl 1% newborn fish food. This newborn fish food was mainly composed of protein, and the fat only contributed to 2.6%. We have added this information to our method section (Line 575-580). To address the reviewers' question of whether fasting affects EECs' response to LA, we compared EECs' response toward Linoleic acid in fed and unfed zebrafish. Our data suggested that the unfed zebrafish and fish fed with a normal diet display comparable EEC cytoplasm and mitochondrial calcium activation (**New data in Figure S15, Result section Line 362-364**). This new result is consistent with our previous finding that a normal diet did not alter EECs' cytoplasm calcium response toward linoleic acid stimulation (Ye et al., 2019).

To address the reviewers' question regarding EECs' glucose response, we stimulated zebrafish with 10% glucose. Our new results suggest that similar to linoleic acid stimulation, a subset of EECs is activated by glucose. Glucose stimulates EECs' cytoplasm and mitochondrial Ca^{2+} (**New data in Figure S13, Video 13, and Text Line 357-359**).

NOTE: We have removed unpublished data that had been provided for the referees in confidence.

Figure 2. Expression of microbially regulated genes associated with mitochondria and actin function in different EEC subtypes.

7. Are the immature EECs in the GF fish mapping back to any of the subtypes identified in S1? Is there a particular population that requires microbial stimulation, and are there any candidates for required receptors that mediate microbial signals in the GF EECs that fail to mature?

To address the reviewer's comments, we examined the expression of the genes that significantly changed between GF and CV zebrafish EECs in different EEC subtypes. We reasoned if commensal microbiota colonization affects a specific EEC subtype (like the reviewer suggested), the mitochondrial-associated genes that are upregulated in CV EECs will be expected to show enrichment in a specific EEC subtype. However, that hypothesis is not supported by our analysis result on the left.

Our results suggest the possibility that commensal microbiota colonization may promote mitochondrial function and actin rearrangement in all the EEC subtypes. To rigorously test whether and how each specific EEC subtype is affected by commensal microbiota colonization,

© 2024. Published by The Company of Biologists under the terms of the Creative Commons Attribution License (https://creativecommons.org/licenses/by/4.0/).

developing new genetic models to measure EECs' function in different EEC subtypes is needed. We currently do not have those tools to test specific EEC subtype functions.

We have not yet identified the receptor(s) that mediate microbial signals to promote EEC maturation. We think the microbial mechanisms underlying EECs' mitochondrial and actin regulation are beyond the scope of the current manuscript. Extensive experiments will be needed in future investigations to address this question.

EECs express different receptors that may sense microbial information. For example, microbially derived short chain fatty acid regulates EECs via free fatty acid receptor 2 (FFAR2). Tryptophan metabolites generated by gut bacteria may also affect EECs through Aryl Hydrocarbon Receptor (AhR), Pregnane X receptor (PxR), or Transient receptor potential ankyrin 1 (Trpa1). In addition, EECs were also known to interact with microbially modified bile acids through G protein-coupled bile acid receptor 1 (GPBAR1). Whether and how these receptor signaling are involved in the microbially regulated EEC mitochondrial function and maturation requires extensive studies that beyond the scope of current manuscript.

Smaller concerns

1. The introduction does not adequately detail the intestinal development of organisms that feed off yolk – like the animals used in this study.

We thank the reviewer's comments and have updated the introduction and result sections and added more information about zebrafish intestine development at the beginning of the result section.\

2. The pyyb gene encodes peptide YY; however, in the text (116) the authors say it is pancreatic polypeptide B. I think this is just a typo in the text, but it is interesting that PYY expression was found enriched proximally, when it is enriched distally in mammals. Pancreatic polypeptide (PP) should be enriched proximally, hence my confusion.

We thank the reviewer's comments. The reviewer is correct, the gene pyyb encodes peptide YY. We apologize for the typo error. We have now corrected the text.

Several other studies using In Situ Hybridization and immunofluorescence staining also indicate that in zebrafish, PYY is enriched in the proximal intestine (Lavergne et al., 2020, Ye et al., 2019). It is unclear why the PYY hormone distribution patterns differ between zebrafish and mammals.

3. Quantifications in Figure 1 are not consistent – why are some hormones counted as a percent, and others as cell number? How were these counts normalized (ie, length of intestine, total cell number, etc)?

We thank the reviewer's comments. We have updated the presented data in Figure 1 (New Figure 2). In our original submission, the number of the intestinal gcga+EEC and sst2+EEC were obtained via live imaging of the *Tg(gcga:EGFP), Tg(sst2:RFP) GF,* and CV zebrafish. To address the reviewer's concern and more rigorously test how commensal microbiota colonization affects gcga+EEC and sst2+EEC subtype, new gnotobiotic experiments in *Tg(gcga:EGFP);Tg(neurod1:RFP)* and *Tg(sst2:RFP); Tg(neurod1:EGFP)* were performed (**New figure 2 F-I', M-N**). The proximal intestinal gcga+EECs and sst2+EECs were now normalized to the total neurod1+ EEC number, and the percentage of gcga+EEC and sst2+EEC in the proximal intestine was presented (**New figure 2 F-I', M-N**).

4. In Figure 4, the distribution of mitochondrial location should also be counted in the GF animals. As it is, only 5 CV EECs were counted for Figure 4E. How did the authors choose which cells to count, as there is even a difference in CV EEC mitochondrial localization shown in 4C and 4G. Since ~85% (Figure 3) to ~89% (Figure 4H) of GF EECs appear to mature normally, how will the authors ensure that any GF EEC mitochondria counted will be representative?

We apologize for the confusion. In our original submission, we performed live imaging of GF

© 2024. Published by The Company of Biologists under the terms of the Creative Commons Attribution License (https://creativecommons.org/licenses/by/4.0/).

and CV zebrafish and analyzed mitochondrial hotspot formation in 170 EECs from 9 GF zebrafish and 194 EECs from 6 CV zebrafish (Fig. S9 J-K) (Fig. 4H-I in original submission). To further confirm that commensal microbiota affects EECs' mitochondrial distribution, three more independent experiments were performed, and EECs' mitochondria were analyzed in fixed zebrafish samples. 3338 EECs from 36 CV zebrafish and 2697 EECs from 34 GF zebrafish were examined. All our experiments repeatedly support the conclusion that commensal microbiota colonization promote the mitochondrial hotspot formation in EECs and enhance mitochondria accumulation at the EECs' base (New data Figure 4 I-N).

We agree with the reviewer that in the original submission, the data presented in Fig. 4E represents a small number. In our original manuscript, mitochondrial live imaging was performed (original Figure 4). To better reveal the mitochondrial distribution pattern within EECs, we repeated the experiments with fixed samples in this revised manuscript (**New Figure 4A-E'**). An increased number of EEC and zebrafish samples (36 EECs from 5 3dpf zebrafish and 36 EECs from 5 7dpf zebrafish) were analyzed to reveal the mitochondrial distribution pattern during the development (**New Figure 4G-H**). Figure 4 is now arranged as supplemental Figure 9. In addition, we have also repeated the experiments with live imaging and increased the number of EECs (25 EECs from 5 3dpf zebrafish and 25 EECs from 5 6dpf zebrafish) used to analyze the mitochondrial distribution pattern (**New Figure S9 A-G**).

5. After stimulation (Figure 7), how long is the increased mitochondrial Ca++ signal maintained? Figure 7H suggests a quick drop, but does not extend the time long enough to see if it ever returns to baseline. What happens after a second stimulation?

Mitochondrial Ca^{2+} was maintained throughout our imaging periods (15 mins) (**New data in Figure S12**). The reason why in Figure 7H, the mitochondrial/cytoplasm Ca^{2+} ratio seems to have a quick drop is because there is a second Ca^{2+} peak. When there is a second Ca2+ peak, cytoplasm Ca2+ amplification is comparable between the first and second peak (**Figure 7H and Figure S12D-E**). However, mitochondrial Ca^{2+} peak is much smaller compared with the first Ca^{2+} peak. This will result in a decreased mito/cyto Ca^{2+} ratio (**Figure 7H and Figure S12D-E**). Neither cytoplasm nor mitochondria will increase Ca^{2+} with an immediate second linoleic acid stimulation. Our previous study also demonstrated that fatty acid pretreatment silences EECs' cytoplasm Ca^{2+} response toward fatty acid stimulation (Ye et al., 2019).

***** Reviewer 2 Advance Summary and Potential Significance to Field:

The manuscript by Ye and colleagues entitled: "Gut microbiota regulates the nutrient sensing enteroendocrine cell maturation and mitochondrial function" reports exciting new insights into the calcium dynamics of enteroendocrine cells (EECs) as they mature, respond to gut microbes, and respond to dietary stimuli. This study is the first to describe calcium dynamics, including changes in mitochondrial calcium dynamics, in the EECs of living, developing animals.

Reviewer 2 Comments for the Author:

Major comments:

1. Last statement of abstract: "Selectively manipulating gut microbial signals to alter EEC mitochondrial function will provide new opportunities to change gut-brain nutrient sensing efficiency and feeding behavior" would better belong in the discussion. The manuscript does not discuss gut-brain signaling or feeding behavior.

We thank the reviewer's comment. We agree with the reviewer that our results did not include data regarding gut-brain communication or feeding behavior. We have now removed this sentence from the abstract and modified our discussion accordingly.

2. The authors report cutting edge in vivo analysis of enteroendocrine cells, but a limitation of the study is the lack of clarity about the extent to which the reported data are representative across independently performed experiments. This is important because the maturation of the EECs is evidently dependent on the microbiota, which will establish somewhat differently between

© 2024. Published by The Company of Biologists under the terms of the Creative Commons Attribution License (https://creativecommons.org/licenses/by/4.0/).

independently reared clutches of zebrafish. The authors are describing new cell biological phenomena and it is important for them to document the data in such a way that the reader can evaluate sources of biological variance. For example: are EECs from the same individual fish more similar than EECs from different fish from the same clutch? From different clutches? Are there rostral to caudal differences in EECs maturation (as for example is seen with the maturation of enteric neurons)? In the graphs, it would be helpful to use color or symbol designations to indicate EECs from the same versus different individuals from the same or different clutches. Specific comments about the figures:

We thank the reviewer's comments. We have now specified in detail in the figure legend regarding the derivation replications, and the zebrafish samples from different derivation experiments were colored differently. Below are the detailed responses to the specific concerns of each figure. For each gnotobiotic experiment, at least two independent derivation experiments were performed, and the same conclusion was reached. For each derivation experiment, we combined eggs from three different clutches, and for each experimental group, the derived germ-free zebrafish eggs were divided into three culture flasks. The samples were collected from the three flasks to prevent flask effects. For live imaging that is involved with gnotobiotic, due to the technique difficulties (such as time required for sterility testing, fluorescence sorting, embryo mount, and confocal imaging) and the fact that different experimental groups are needed to finish imaging on the same day, the zebrafish sample number that we were able to achieve for each gnotobiotic experiment can be limited.

The enteric neurons are derived from neuron crest cells, which migrate and mature from rostral to caudal. Unlike the enteric neurons, EECs were generated from the intestinal epithelium. To answer the reviewer's concern regarding the rostral to caudal difference in EECs maturation, we conducted new experiments to analyze the distal intestinal EECs' actin filaments (**New data in Figure S1, Text Line 122-125**). Our new data revealed that EECs in the distal intestine re-arrangement their actin filaments during maturation (**Figure S1A-B**). However, at 4dpf, a similar percentage of EECs display active actin filaments between the proximal and distal intestine (**Fig.S1C-F**), suggesting that the proximal and distal intestinal EECs may mature simultaneously.

This manuscript is focused on the nutrient-sensing EECs. Similar to mammals, the nutrient-sensing EECs primarily resided in the proximal intestine. We primarily analyzed EECs from the proximal intestine. We have specified the analyzed intestine region in each figure legend and main text in this revised article.

Figure 3G: Clarify how many total EECs were quantified per individual fish. Clarify how many independently derived clutches of zebrafish were tested and whether there was clutch to clutch variation.

Following the reviewer 1's comments above (comment 4), we rearranged the paper figures. Figure 3 is now updated as Figure 1. Figure 3G is now Figure 1K. Pooled samples from three independently derived clutches of zebrafish were analyzed. The samples from different derivation experiments were colored with different colors. Each dot represents one zebrafish larvae. 2597 EECs were analyzed in CV, and 2043 EECs were analyzed in GF. The same trend was observed in all three different derivations experiments. This information is also provided in Figure 1's figure legend.

Figure 4E: It is not obvious how the quantification for the 3 dpf mitochondrial distribution matches the images. Shouldn't the mitochondrial intensity diminish through the nucleus? The quantification was done for 5 EEC cells from one zebrafish at the two time points. Was this one fish representative of other individuals, especially considering different individuals from different clutches with different microbiotas?

We apologize for the confusion regarding the quantification of Figure 4E (Figure 4G, H in the current revision). We added a supplemental **Figure S8** to explain the method we use to quantify mitochondrial distribution patterns within EECs. To quantify the intracellular mitochondrial distribution, we first create z-stack projection of an EEC. Instead of drawing a line from apical to basal to obtain the mitoEOS fluorescence intensity plot profile, we circle the EEC with a rectangle and obtain the mean mitoEOS fluorescence intensity plot profile in the basal to apical direction. Even

© 2024. Published by The Company of Biologists under the terms of the Creative Commons Attribution License (https://creativecommons.org/licenses/by/4.0/).

though mitochondria are not located inside the nucleus, they are located in the cytoplasm around nucleus in 3 dpf EECs. Therefore, for 3 pdf EECs, there is an evenly mitochondrial distribution pattern in the basal to the apical axis.

To confirm our findings, in this revised article, we performed new live imaging tracing and expanded the sample size. 25 EECs from 5 3dpf zebrafish and 25 EECs from the same 5 6dpf zebrafish are now quantified. This data is not presented in **Figure S9A-G**. The 5 zebrafish were from two different clutches.

In addition to the live imaging tracing, during this revision, we also performed new experiments and examined intracellular mitochondria distribution in 3dpf and 7dpf fixed samples (Figure 4 A-H). Because we can use slower scanning speed in fixed tissues, fixed tissue imaging allows us to obtain higher-resolution images to visualize the intracellular mitochondrial distribution better. The even mitochondria distribution pattern in 3dpf EECs and punctate or hotspot mitochondria distribution pattern in 7dpf EECs is also presented and quantified in these fixed samples in Figure 4A-H. For Figure 4A, 573 EECs from 8 3dpf zebrafish and 1161 EECs from 12 7dpf zebrafish were analyzed. For Figure 4G-H, 36 EECs from 5 3dpf zebrafish and 36 EECs from 5 7dpf zebrafish were analyzed.

Figure 4H: Clarify how many total EECs were quantified per individual fish.

Figure 4H is now updated as Figure 4M. 3338 EECs from 36 CV zebrafish and 2697 EECs from 34 GF zebrafish were analyzed. Each dot represents an individual zebrafish. Zebrafish samples pooled from three independent derivation experiments were analyzed (samples from one derivation experiment were labeled by the same color).

Figure 4 H & I: Clarify how many independently derived clutches of zebrafish were tested and whether there was clutch to clutch variation.

Samples pooled from three independent derivation experiments were analyzed.
The same trend was observed in the three independent experiments.

Figure 5F-H: Provide quantification of a single fish for each time point. Are these measurements representative of other individuals? Is there rostral to caudal variation in the EEC values quantified?

For the experiment performed in Figure 5, we traced 10 zebrafish from 3dpf to 6dpf. Figure 5F-H quantifies one representative zebrafish Ca^{2+} dynamics from 3dpf to 6dpf. Such Ca^{2+} dynamic change is consistent in other zebrafish. The collective data is presented in Figure 5I-K. We did not observe a variation between the rostral and caudal sections in the proximal intestinal region that we imaged.

Figure 5I-K: Clarify the providence of the 10 EECs quantified. Are they from one individual? Multiple individuals? Multiple clutches?

For the experiment performed in Figure 5, we traced **10 zebrafish** (not 10 EECs) and performed live imaging from 3dpf to 6dpf. The zebrafish were from three different clutches. During our revision, we realized that one of the zebrafish died in the midst of the experiment, and data was missed for later time points for that zebrafish. We removed that fish from our dataset. The data in 5I-K now includes 277 EECs for 9 3dpf zebrafish, 437 EECs from 9 4dpf zebrafish, 684 EECs from 9 5dpf zebrafish, and 727 EECs from 9 6dpf zebrafish.

Figure 6D-F: states that more than 5 zebrafish were quantified for each condition, but does not clarify whether they were fish from the same or different clutches.

Figure 6D-F is updated. 523 EECs from 4 GF zebrafish and 575 EECs from 5 CV zebrafish were analyzed. The GF and CV zebrafish were from the same derivation. EECs from the same GF or CV zebrafish were labeled with the same color. Three independent derivation experiments were performed, and the same trend was observed in all three experiments. Information is provided in the figure legend. For our live imaging data, we need to optimize the imaging conditions for each experiment, and imaging settings differ; therefore, data from different experiments were not

© 2024. Published by The Company of Biologists under the terms of the Creative Commons Attribution License (https://creativecommons.org/licenses/by/4.0/).

pooled together.

Figure 6G-M: clarify whether these are EECs from a single or multiple zebrafish.

581 EECs from 4 GF zebrafish and 398 EECs from 5 CV zebrafish were analyzed. A second independent derivation experiment with 5 GF zebrafish and 5 CV zebrafish was performed, and the same conclusion was reached. Information is provided in the figure legend.

Figure 7: The end of the figure legend states that more than 100 EECs from at least 3 zebrafish were analyzed, but it is not clear to which parts of the figure this statement applies.

We apologize for the confusion. This refers to Figure 7K-P. We have now updated the figure legend.

Figure 8: Clarify which EECS correspond to which individual zebrafish and whether zebrafish from multiple independent clutches were analyzed.

We have now color code the EECs in Figure 8 C-D. The EECs from the same zebrafish were labeled with the same color. A second independent derivation experiment with 7 GF zebrafish and 7 CV zebrafish was performed. The same conclusion is reached.

3. The image analysis method by which EECs' shapes and signal intensities were quantified are crucial for evaluating the data reported, but insufficient information is provided. The methods refer to the Nikon NLS element software. How does this software segment the elements? To what extent is this user-specified? A supplemental figure providing more details about the cellular segmentation approach would be valuable.

We thank the reviewer's comments. We have now added more detail in the method section and included a supplemental figure and two videos (Figure S11, video 7-8) to illustrate how we perform segmentation using the NLS element software.

4. As a proof of concept that the calcium distributions reported in EECs are accurately measured, the authors should include some pharmacological manipulations that alter these distributions, for example acute treatment with a mitochondrial membrane depolarizing agent that would be expected to disrupt mitochondrial calcium uptake.

We thank the reviewer's comments. The *Tg(neurod1:mitoRGECO)* used in our paper was reported and used in previous studies (Mandal et al., 2021, Mandal et al., 2018). We have also followed the reviewer's suggestion and performed new experiments. Our new data showed that when disrupting mitochondrial inner membrane potential using Carbonyl cyanide-p-trifluoromethoxyphenylhydrazone (FCCP), a decreased mitoRGECO fluorescence in EECs was recorded (**New data in Fig. S10, Text in Line 297-301**).

Minor comment:

Line 36: jarring switch from "all organisms" to "during development, the fetus…": need to clarify that the second statement is about placental mammal development, not all organisms. It is confusing to introduce the EECs in the context of mammals when the paper will study them in non-mammals.

We thank the reviewer's comments. We have now modified our introduction to match with the zebrafish organism that we used in our study.

***** Reviewer 3 Advance Summary and Potential Significance to Field:

This manuscript analyzes how mitochondrial activity in EECs changes from day 3-day 6 in zebrafish, and examine how this compares to germfree zebrafish. Most of the figures (aside from figure 1 as questioned below) seems to support that colonization broadly induces EEC maturation and mitochondrial response in the presence of the long-chain fatty acid linoleic acid. Overall, the

© 2024. Published by The Company of Biologists under the terms of the Creative Commons Attribution License (https://creativecommons.org/licenses/by/4.0/).

general examination of EEC mitochondrial responses seem sound and consistent with EEC maturation over time. While the studies then testing dependence of these changes on colonization also generally align with the author's interpretation, addressing points below would help strengthen conclusions and biologic depth.

Reviewer 3 Comments for the Author:

1. Figure 1 suggests that some subsets of EECs are decreased following commensal microbiota colonization -Gcg/GLP-1+EECs in the proximal intestine and Trpa1+EECs in the distal intestine (Fig. 1F-I, L-N), whereas others are unchanged. However, the transcriptional analyses in Figure 2 support instead an increase in EEC specific gene expression profiles (Fig.2C- 74.5% of upregulated genes are enriched in EECs). How are these differences reconciled? It would seem that promoting EEC maturation would be reflected as an increase in EEC numbers?

We thank the reviewer's comments. We apologize for the confusion. Our data suggest that gut microbiota colonization promotes EEC maturation but not EEC formation. When the EECs mature, it is expected that the genes involved with specialized EEC functions (such as vesicle secretion, membrane potential regulation, and so on) will increase. However, when more EECs are formed from IECs (if the EEC maturation process remains the same), when sorting the EECs and analyzing the gene expression, the genes that are involved in the specialized EEC function may not increase.

This is indeed consistent with our RNAseq data from FACS sorted EECs. The majority of the 74.5% of CV upregulated genes that are enriched in EECs are genes that are involved in membrane potential regulation, hormone vesicle packing, and vesicle secretion, see Figure 3E (Figure 2E in the original submission). Our RNAseq data, together with other data provided in this manuscript, lead us to the conclusion that gut microbiota colonization promotes EEC maturation.

The *neurod1* marker used in our study labels both immature and mature EECs, as demonstrated by previous studies (Gehart et al., 2019). We have now also provided new data showing that immature EECs in 3dpf zebrafish display the same *neurod1:EGFP* fluorescence intensity as EECs in 6dpf zebrafish (**see new data in Figure S6A-C and in the result section line 192-195**). Previous mammalian studies demonstrate that both immature and mature EECs express hormone markers (Gehart et al., 2019, Beumer et al., 2018). It is also shown that when EECs migrate from crypt to villi (transition from a relatively immature to mature state), EECs switch hormone profiles (Beumer et al., 2018). For example, for L-cells (the EECs that express GLP-1, *Gcg*+EECs) in the crypt express high GLP-1, but the GLP-1 level decreases when L-cells move to the villus, suggesting high GLP-1 expression may represent a relatively immature state for L-cells (Beumer et al., 2018). In our experiments, we found that a higher percentage of EECs express *gcga:EGFP* and *gcga:EGFP* expression level is higher in GF zebrafish (New Figure 2H- I', and Figure 2N). This is consistent with other data in our manuscript that EECs in GF zebrafish fail to mature appropriately.

Unfortunately, we currently do not have gene markers that allow us to label immature and mature EECs. The markers that were used in Figure 2 (Figure 1 in the original submission) can examine EEC subtypes but not the EEC maturation states. To more rigorously characterize how gut microbiota affects EEC subtypes, in this revised submission, we perform new experiments to examine the different EEC subtypes in an intestinal region-specific manner (**Figure 2 and Figure S6D-G, Text Line 189-204**). Our updated results indicate that commensal microbiota did not alter the total neurod1+EEC number in the proximal intestine (Fig.2 J). However, commensal microbiota colonization increases the percentage of *sst2*+EECs in the proximal intestine but decreases the percentage of *gcga*+EECs (Fig.2 M-N). In the middle and distal intestine, the total EEC number labeled by *Tg(neurod1:EGFP)* is not changed between GF and CV zebrafish (Fig. S6D). The number of *sst2*+EEC, *gcga*+EEC, and *trpa1*+EEC increases in the middle and distal intestine of the GF zebrafish (Fig. S6E-G). It is possible that the favored specification of these EEC subtypes in GF zebrafish's middle and distal intestines may come at the cost of other EEC subtypes, such as Calca+EEC (EEC3). However, we currently do not have immunofluorescence or genetic markers to label the Calca+EEC subtype. Whether GF zebrafish exhibit a reduced number Calca+EECs in middle and distal intestine remains to be investigated in future studies. <u>These immunofluorescence data are consistent with the RNAseq data.</u> For example, some EEC hormone genes are increased in GF zebrafish EECs (Figure 3E). Trpa1+EEC-associated hormones *adcyap1a* and *tph1b* (the enzyme that

© 2024. Published by The Company of Biologists under the terms of the Creative Commons Attribution License (https://creativecommons.org/licenses/by/4.0/).

leads to Serotonin production) are increased in GF EECs (Figure 3E). In addition, insl1b, a hormone that expressed in *gcga*+EEC subtype, is also increased in GF EECs (Figure 3E). This RNA seq data is consistent with our observation that GF zebrafish displayed an increased Trpa1+EECs in the distal intestine and an increased gcga+EECs in the proximal and distal intestine. Together, our data suggests that commensal microbiota colonization may affect the specification of different EEC subtypes.

2. Can the localization of microbiota along the length of the intestine be demonstrated in relation to changes in observed in Figure 1, during the 3-4 days between colonization to analyses?

We thank the reviewer's comments. In general, it is thought that the proximal intestinal region in zebrafish has a bigger luminal space to hold more microbial species than the distal region. However, the regional microbial abundance and composition along the intestine length have not been clearly mapped in the zebrafish larvae (to our knowledge). The small size of the zebrafish intestine hinders the usage of 16S or metagenomic tools from studying the zebrafish larvae's gut microbial localization in an intestinal region-specific manner, and our lab does not have the microbial tools to study the microbial localization. Therefore, even though gut microbiota localization may affect EEC subtype specification in different intestinal regions, we do not have the methods and tools to test the hypothesis experimentally. The microbial mechanisms (including microbiota localization) underlying microbial regulation of the EEC subtypes require future studies and are beyond the scope of this manuscript.

3. The study thoroughly examines mitochondrial activity from day 3-6 dpf in zebrafish (Fig. 5). The assumption is that these data are representative of colonized zebrafish, although not clearly designated as CV. The comparison between GF and CV in the next figure lacks the same level of depth in evaluation. Its not clear why the y axes values differ between Fig. 6 and Fig. 5 for CV. Also the differences shown in Fig 6D-F are very small despite the stars for stats indicating significance in EEC cytoplasm and mitochondria calcium activity. The issues are similar comparing Fig. 7 and Fig. 8. These points should be addressed to assess technical and biological significance of these studies in relation to the microbial colonization.

We thank the reviewer's comments. For Figure 5, we used conventionally raised zebrafish that are normally raised and have commensal microbiota colonization. We updated the Figure 5 legend to clarify it. The y-axis values differ between Figure. 6 and Figure 5 because they are two independent experiments. We used the same confocal settings (such as the same zoom, laser power, and gain) in all the samples in Figure 5.
When we performed the experiments in Figure 6, we optimized the image capture for those experiments. We used the same imaging setting for all the samples in Figure 6. However, the 488nm and 568nm laser power and gain in Figure 6 are different from Figure 5. Therefore, the GF group and CV group in Figure 6 can be compared to each other. However, the values in Figure 6 and Figure 5 cannot be compared. We have also updated the method section (Line 671-674) to clarify.

Our data in Figure 6 revealed that gut microbiota colonization induces small but significant effects on the absolute level of the basal cytoplasmic and mitochondrial Ca^{2+}. This small but significant difference was repeatedly observed in three independently performed gnotobiotic experiments. Our data showed that gut microbiota has **significant effects on EECs' cytoplasmic and mitochondrial Ca^{2+} dynamic** (Figure 6 G-M). The presence of gut microbiota induces EECs' cytoplasmic and mitochondrial Ca^{2+} fluctuation. As reflected by Figure 6J and N, only 2.5% of GF EECs are active, while 21% of EECs are active in CV zebrafish (8.4 fold increase). Therefore, we believe the phenomenon described in our manuscript is biologically significant.

For Figure 7, we assume the reviewer refers to Figures 7O and P since these are the only data in this figure that used statistical analysis. The fluorescence sensor iATPSnFR measures the ATP level in the EEC cytoplasm. The abundance of the cytoplasmic ATP level is determined by both ATP generation as well as ATP consumption. When we stimulate EECs with linoleic acid, the activated EECs will increase ATP consumption due to the need for vesicle release, vesicle recycling, and membrane potential maintenance. Our data suggested that those activated EECs also exhibit increased mitochondrial Ca^{2+}, suggesting an increase in ATP production. Indeed, using our ATP sensor, we observed a small but significant increase in intracellular ATP levels in the stimulated conditions (Figure 7P). The reason that the increased amplitude is relatively small is likely because

© 2024. Published by The Company of Biologists under the terms of the Creative Commons Attribution License (https://creativecommons.org/licenses/by/4.0/).

we are measuring the net effects of both ATP consumption and ATP generation.

For Figure 8, we disagree with the reviewer's comments that the difference observed in GF and CV zebrafish is small. Figure 8B shows that the percentage of linoleic acid activated EECs increased from 25% in GF zebrafish to 35% in CV zebrafish (40% difference). The Ca^{2+} amplitude in activated cells is measured via the difference between Fmax and F0, in other words, Fmax-F0. To better illustrated the difference between GF and CV zebrafish, we replotted Figure 8C and D and changed the Y-axis to ΔF/F0. As illustrated by our figure, there are no differences between cytoplasmic Ca^{2+} amplitudes comparing GF and CV zebrafish. However, the mitochondrial Ca2+ amplitude increased 2 folds in CV EECs. We also added the representative linoleic acid activated EEC Ca2+ profile for GF and CV EECs in this revised manuscript (Figure 8G-J). We have also included new plots showing the correlation between cytoplasm Ca^{2+} activation and mitochondrial Ca^{2+} activation in GF and CV zebrafish (**New data in Figure 8E-H**). A positive correlation was observed in Linoleic acid-activated EECs in CV but not in GF zebrafish (**Figure 8E, F,** P=0.11 for GF EECs and P=0.0048 for CV EECs). Together, our data strongly demonstrate that in the absence of commensal microbiota, EECs' mitochondria fail to respond to nutrient stimulation.

We think our studies provided both conceptual and technique advances to the field to increases our understanding of the development and plasticity of the EECs.

Conceptual advances provided by our work

(1) The EECs are different during development. Using the development tracing in the zebrafish model, our study reveals for the first time the EECs' at the embryonic stage exhibit striking differences in morphology, cellular activity, and mitochondrial activity. Moreover, our study demonstrates the development and maturation process of the EECs are plastic. Environmental factors such as gut microbiota are important in guiding this development process. The development and maturation of the EEC system will be part of the critical events that lay the foundation of the intestinal sensory system. Understanding the formation of this system during development will provide vital insights that will guide research in postnatal nutrient sensing, nutrient metabolism, and gut homeostasis.

(2) The increased mitochondrial function is a hallmark of mature EECs. Mitochondria is a key organelle that regulates many cellular processes. The function of mitochondria has been extensively studied in the nervous system, pancreatic endocrine cells, and cancer biology. The interaction between mitochondria and the development gained extensive interest in recent years. However, little is known about the function and regulation of the mitochondria in EECs. Our study provides the first evidence that (a) the mitochondrial function is tightly linked with the EECs' nutrient sensing and vesicle secretion function; (b) the mitochondrial function is associated with the EECs' maturation events during development; and (c) the commensal microbiota colonization is indispensable in EECs' maturation and mitochondrial function.

(3) Microbiota colonization is important for the EECs' maturation and mitochondrial function. It is well known that gut microbiota is critical in regulating energy metabolism and feeding behavior (*Cani P. et al., Nature Metabolism, 2019*). Whether the microbial-mediated metabolic and feeding behavioral change is associated with the EECs remains unclear. Our studies reveal for the first time that gut microbiota regulates EECs' maturation and mitochondrial function during development, providing new mechanisms to understand microbial-mediated metabolic and feeding behavior change.

Technique advances provided by our work

(1) Trace individual EECs' cellular activity and perform the spatiotemporal EEC activity analysis systemically. The EECs are rare sensory cells in the intestine epithelium and comprise less than 1% of all intestinal epithelial cells (*Sanchez JG et al., 2022; Furness JB. et al., 2013*). Moreover, the EECs are sparsed and distributed along the whole digestive tract (*Sanchez JG et al., 2022; Furness JB. et al., 2013*). These features make the EECs extremely difficult to study in vivo. Historically, the in vivo EECs' activity was measured through hormonal abundance in the blood circulation. However, the blood hormonal profile does not reflect the spatiotemporal EECs' activity in real-time. EECs have also been studied intensively using the cell or organoid culture system. However, the in vitro cell or organoid culture can not recapitulate the process during development. Here, using the genetic models in the zebrafish model and novel imaging analysis, our study is the first that is able to image the EECs' activity in the entire live animal and track and analyze the

© 2024. Published by The Company of Biologists under the terms of the Creative Commons Attribution License (https://creativecommons.org/licenses/by/4.0/).

individual EECs' activity in the systemic setting.
Therefore, our study provides novel methods to understand the EECs' function in vivo in a cellular, spatial, and temporal resolution.

(2) New techniques to image and analyze the EECs' mitochondrial dynamic, activity, and function in live zebrafish. The mitochondrial function has been routinely studied using chemical dyes and sea horse measurements (*Connolly N. et al., 2017*). However, those methods can not be used to analyze the mitochondrial function in a cellular-specific manner in live animals. Using the newly developed genetic tools, we developed new methods to express the genetically encoded mitochondrial sensor in EECs. Through these methods, we were able to image and analyze the EECs' mitochondrial function in vivo for the first time. Our study is the first that reveals the EECs' mitochondrial dynamics and activity in live animals. Considering little is known about the mitochondrial function in EECs, the novel methods presented in our paper will be impactful in mitochondrial biology in EECs and nutrient sensing.

References

BEUMER, J., ARTEGIANI, B., POST, Y., REIMANN, F., GRIBBLE, F., NGUYEN, T. N., ZENG, H., VAN DEN BORN, M., VAN ES, J. H. & CLEVERS, H. 2018. Enteroendocrine cells switch hormone expression along the crypt-to-villus BMP signalling gradient. *Nat Cell Biol*, 20, 909-916.
GEHART, H., VAN ES, J. H., HAMER, K., BEUMER, J., KRETZSCHMAR, K., DEKKERS, J. F., RIOS, A. & CLEVERS, H. 2019. IdenWficaWon of Enteroendocrine Regulators by Real-Time Single-Cell DifferenWaWon Mapping. *Cell*, 176, 1158-1173 e16.
LAVERGNE, A., TARIFENO-SALDIVIA, E., PIRSON, J., REUTER, A. S., FLASSE, L., MANFROID, I., VOZ, M. L. & PEERS, B. 2020. PancreaWc and intesWnal endocrine cells in zebrafish share common transcriptomic signatures and regulatory programmes. *BMC Biol*, 18, 109.
MANDAL, A., PINTER, K. & DRERUP, C. M. 2018. Analyzing Neuronal Mitochondria in vivo Using Fluorescent Reporters in Zebrafish. *Front Cell Dev Biol*, 6, 144.
MANDAL, A., WONG, H. C., PINTER, K., MOSQUEDA, N., BEIRL, A., LOMASH, R. M., WON, S., KINDT, K. S. & DRERUP, C. M. 2021. Retrograde Mitochondrial Transport Is EssenWal for Organelle DistribuWon and Health in Zebrafish Neurons. *J Neurosci*, 41, 1371-1392.
YE, L., MUELLER, O., BAGWELL, J., BAGNAT, M., LIDDLE, R. A. & RAWLS, J. F. 2019. High fat diet induces microbiota-dependent silencing of enteroendocrine cells. *Elife*, 8.

Second decision letter

MS ID#: DEVELOP/2023/202544

MS TITLE: Gut microbiota regulates the nutrient sensing enteroendocrine cell maturation and mitochondrial function

AUTHORS: Alfahdah Alsudayri, Shane Perelman, Melissa Brewer, Annika Chura, Madelyn McDevitt, Catherine Drerup, and Lihua Ye
ARTICLE TYPE: Research Article

I am happy to tell you that your manuscript has been accepted for publication in Development, pending our standard ethics checks.

Reviewer 1

Advance summary and potential significance to field

Alsudayri and colleagues have submitted a fantastic revision of the original manuscript. This revised version is excellent and all my previous concerns have been addressed.

Comments for the author

© 2024. Published by The Company of Biologists under the terms of the Creative Commons Attribution License (https://creativecommons.org/licenses/by/4.0/).

Prior to publication, I encourage the authors to screen for typos. Here are a few:

Line 165, GLP-1 should be Glucagon-like peptide
Figure 8 C-D, should be amplitude
Line 775, should be evenly
Line 858, should be unstimulated
Figure S15 E-F, should be amplitude
Figure S16 Legend, change exabit to exhibit

Reviewer 2

Advance summary and potential significance to field

The manuscript by Ye and colleagues entitled: “Gut microbiota regulates the nutrient sensing enteroendocrine cell maturation and mitochondrial function” reports exciting new insights into the calcium dynamics of enteroendocrine cells as they mature, respond to gut microbes, and respond to dietary stimuli. This study is the first to describe calcium dynamics, including changes in mitochondrial calcium dynamics in the EECs in living, developing animals.

Comments for the author

The revised manuscript contains extensive additional experimental evidence that provides strong support for findings reported in the original submission. The authors have provided thoughtful responses to all of the issues raised in the review of the original manuscript. The revised and reorganized manuscript is much improved.

Reviewer 3

Advance summary and potential significance to field

Examination of microbial impact on nutrient regulation of EEC mitochondrial responses and maturation

Comments for the author

No further comments. The authors have adequately addressed my concerns.

© 2024. Published by The Company of Biologists under the terms of the Creative Commons Attribution License (https://creativecommons.org/licenses/by/4.0/).
